# A newly bat-borne hantavirus detected in Seba’s short-tailed bats (*Carollia perspicillata*) in the Brazilian Atlantic Rainforest

**DOI:** 10.1590/0074-02760240132

**Published:** 2024-12-16

**Authors:** Patrick Jesus de Souza, Jorlan Fernandes, Thayssa Alves Coelho, Matheus Cosentino, Mirela D’arc, Patrícia Dias Galvão Alves, Alexandro Guterres, Emmanuel Messias Vilar, Elba Regina Sampaio de Lemos, Pedro Cordeiro-Estrela, André Felipe Andrade Santos, Renata Carvalho de Oliveira

**Affiliations:** 1 Fundação Oswaldo Cruz-Fiocruz, Instituto Oswaldo Cruz, Laboratório de Hantaviroses e Rickettsioses, Rio de Janeiro, RJ, Brasil; 2 Universidade Federal do Rio de Janeiro, Departamento de Genética, Laboratório de Diversidade e Doenças Virais, Rio de Janeiro, RJ, Brasil; 3 Universidade Federal da Paraíba, Departamento de Sistemática e Filogenia, Laboratório de Mamíferos, João Pessoa, PB, Brasil

**Keywords:** bat-borne hantavirus, Brazilian Atlantic Rainforest, hantavirus, Carollia perspicillata, chiroptera

## Abstract

**BACKGROUND:**

Bat-borne hantaviruses have been identified worldwide but little is known about neotropical bats in the megadiverse biomes of the American continent. Although serological evidence has hinted at hantavirus circulation in Brazil, the scarce number of genomic detection represents a gap to understand viral diversity, prevalence, and ecology of bat-borne hantaviruses.

**OBJECTIVE:**

We aim to investigate and evaluate the presence and prevalence of bat-borne hantavirus in the Brazilian Atlantic Forest.

**METHODS:**

Here in, 97 lung and kidney tissue samples from bats captured in the Brazilian Atlantic Rainforest were submitted to hantavirus-specific nested reverse transcription-polymerase chain reaction (RT-PCR) targeted the hantaviral L segment and metagenomic analysis.

**FINDINGS:**

Hantavirus RNA was detected in five tissue fragments of 20 Seba’s short-tailed bats (*Carollia perspicillata*). Phylogenetic analysis, based on partial L-segment sequence using maximum likelihood method, demonstrated that the identified virus formed a monophyletic clade and a highly divergent bat-borne lineage comprising other recent strains found in the genus *Carollia* from South America.

**MAIN CONCLUSIONS:**

Our findings suggest the presence of a novel bat-borne hantavirus in Brazil, tentatively named Mamanguape virus (MGPV). Additional genomic data will help to extend our knowledge about the classification of MGPV within the *Hantaviridae* family and the evolution origins of new world bat-borne hantaviruses.

Most of our knowledge about hantavirology is based on rodent-borne hantaviruses within the genus *Orthohantavirus.* These include species such as Andes virus (*Orthohantavirus andesense*), Hantaan virus (*Orthohantavirus hantanense*), and Seoul virus (*Orthohantavirus seoulense*), which are associated with human diseases worldwide. Some orthohantaviruses cause varying severities of haemorrhagic fever with renal syndrome in Europe, Asia, and Africa, while others are linked to hantavirus pulmonary syndrome occurring in the Americas.^1^ ^,^ ^2^ Despite most hantaviruses falling into the *Orthohantavirus* genus (including all rodent-borne hantaviruses), the discovery of distinct hantaviruses from various animal taxa (such as bats, shrews, moles, reptiles, and fishes) led to the proposal of new genera and subfamilies for their classification.^3^ Currently, the *Hantaviridae* family comprises four subfamilies: *Mammantavirinae* (pathogenic and non-pathogenic hantaviruses detected in mammals), *Actantavirinae* and *Agantavirinae* (hantaviruses detected in fish), and *Repantavirinae* (including a hantavirus detected in squamate reptiles).^4^ ^,^ ^5^

The discovery of highly divergent lineages of hantaviruses in bats of different species has provided unlimited opportunities to search for bat-associated hantaviruses, due to the vast geographic distribution and diversity of bats worldwide. Since the first report of Magboi virus in Africa,^6^ novel bat-borne hantaviruses have been described in Africa, Europe, mostly Asia, and very recently in the Americas.^7^ ^,^ ^8^ ^,^ ^9^ ^,^ ^10^

The American continent, despite harbouring one-third of the world’s bat diversity (approximately 350 species across nine families),^11^ remains little explored in terms of bat-borne hantaviruses. Although serological evidence has hinted at hantavirus infections in different Brazilian bat species,^12^ ^,^ ^13^ the detection of a bat-borne hantavirus RNA was only accomplished by 2024, through tissue fragments of heart, lung and kidney in *C. perspicillata* captured in the Cerrado biome.^10^

Brazil, a megadiverse country with continental proportions, is divided into various biomes. Two of these biomes-the Cerrado and the Atlantic Forest-are biodiversity hotspots because of their diversity, endemism, and high proportion of species at risk of extinction. The Atlantic Forest sustains over 64% of Brazil’s total bat population.^14^ ^,^ ^15^ Brazil hosts 15% of the world’s bat diversity and is home to bats associated with zoonotic pathogens, including the widespread *C. perspicillata*.^16^ Our study aims to investigate and evaluate the presence and prevalence of bat-borne hantavirus in the northeastern Brazilian Atlantic Forest. We identified a presumably novel hantavirus provisionally named Mamanguape virus (MGPV) in Seba’s short-tailed bat (*C. perspicillata*, Phyllostomidae). A complete genome characterisation is essential for the accurate taxonomic classification of this new bat-borne hantavirus in Brazil.

## MATERIALS AND METHODS

*Ethics statement* - All trapping and experimental procedures involving bats were conducted in accordance with approved capture licenses from the Instituto Chico Mendes de Conservação da Biodiversidade (SISBIO n 41683) and the Ethics Committee of the Oswaldo Cruz Foundation (FIOCRUZ) (CEUA LW-81/12, P-42/12.1).

**Fig. 1:**
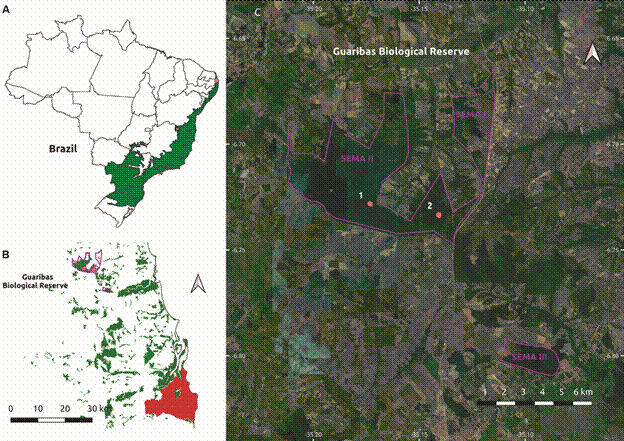
(A) Location of the Guaribas Biological Reserve in Paraíba State, Brazil, positioned at the northern edge of the Atlantic Forest Biome (highlighted in green). (B) Landscape context of the Guaribas Biological Reserve within the limited (~ 11%) Atlantic Forest remnants (depicted in green) of Paraíba State, juxtaposed with its capital, João Pessoa (highlighted in red). (C) Sampling sites of Hantavirus-positive Seba’s short-tailed bat *Carollia perspicillata* within the Guaribas Biological Reserve area SEMA II. 1. Cabeça de Boi; 2. Trilha do Inhão.

*Trap sites and bat sampling* - The bats were trapped in the Guaribas Biological Reserve (GBR), located in the municipality of Mamanguape, in the State of Paraíba, Brazil. The GBR spans 4.051.62 hectares and is a conservation unit of the Atlantic Forest Biome, the second largest tropical rainforest after the Amazon Rainforest (Fig. 1). Situated at the northern limits of the Atlantic Forest in the Northeastern Brazilian region, this portion of the biome is the most deforested area, with approximately 11% of remnants, mostly surrounded by sugarcane monoculture that began in the 16th century. The Guaribas Biological Reserve’s mammalian fauna comprises about 70 species, 60 genera, and eight orders, with 33 species belonging to the order Chiroptera.^17^ Between March 31st and April 09th, 2015, a total of 199 individuals representing seven genera and eight species were collected using ten mist nets with the following specifications: five nets of 12 x 2.5 m, two nets of 7 x 2.5 m, and three canopy nets of 3 x 2.5 m, all with a 20-millimetre mesh. The nets were set up at five sampling points, with a two-night sampling at each point. The animals were identified based on the works of Gardner and reviewed based on Díaz et al.^18^ ^,^ ^19^ The animals collected in the four areas are deposited in the Mammal Collection of the Federal University of Paraíba. After identification, a total of 97 individuals were euthanised and had their organ tissues extracted, such as the lung, liver, and kidneys.^20^ The tissue samples were stored in TRIzol^TM^ reagent (Invitrogen, San Diego, USA) at -20ºC for future molecular analyses.

*RNA extraction, reverse transcription-polymerase chain reaction (RT-PCR) and sequencing* - Total RNA was extracted from the lung and kidney of each bat sample using the TissueLyser LT^®^ (Qiagen, Hilden, Germany) and PureLink RNA^®^ Mini Kit (Invitrogen, San Diego, USA) following the manufacturer’s instructions. Hantavirus-specific RT-PCR was conducted to amplify a fragment of the RNA-dependent RNA polymerase-encoding L segment, utilising published primers.^21^ Amplicons were directly nucleotide sequenced using the BigDye^®^Terminator v3.1 Cycle Sequencing Kit (Applied Biosystems, Foster City, USA) as per the manufacturer’s recommendations, with the reaction run in an ABI Prism 3130x (Applied Biosystems).

*Metagenomic preparation cDNA and sequencing* - One animal with a positive RT-PCR diagnosis in both kidney and lung tissues underwent a viral metagenomics protocol to obtain additional *Hantaviridae* genome information. The previously extracted RNA from lung and kidney tissues was equimolarly mixed into a pool and utilised as a template for cDNA synthesis using the Superscript IV First Strand Synthesis Kit (Invitrogen, United States), followed by dsDNA synthesis with the Klenow fragment 3′-5′ exo (New England Biolabs Inc., United States). A sequence library was constructed using the Nextera XT DNA Library Preparation Kit (Illumina, United States) following the standard protocol. Library molarity was determined through quantification with the QuBit dsDNA HS Assay Kit and fragment size inference with the NEBNext^®^ Library Quant Kit (New England Biolabs, United States). Subsequently, 11 pM of the library was loaded into a MiSeq V2 Nano 300-cycle cartridge (Illumina), and massive sequencing was conducted on the Illumina MiSeq platform at the Department of Genetics of Rio de Janeiro Federal University.

The sequencing data underwent processing through an in-house pipeline. Raw reads shorter than 50 bp and low-quality reads with Phred scores under 30 were removed using Fastp v.0.20.1.^22^ Reads were then mapped against the host genome (*C. perspicillata*, Accession: GCA_004027735-1) with BWA v 0.7.17,^23^ and unaligned reads were de novo assembled with Meta-spades software v.3.15.3.^24^ ^,^ ^25^ Taxonomic assignment of unaligned reads and assembled contigs was performed using Diamond v.2.0.14 with an e-value cut-off of 10-5 and parameters “--more-sensitive” and “--max-target 1”.^26^ A similarity search was conducted against the complete non-redundant protein database from NCBI (as of July 27, 2021). Interactive visualisation plots to identify viral families of interest were generated using Krona v.2.7.1.^27^

*Genetic and phylogenetic analysis* - To contextualise the identified viruses, a viral family database for the RNA-dependent RNA polymerase-encoding L segment was constructed using representative sequences from the *Orthohantavirus*, *Mobatvirus*, *Loanvirus*, and *Thottimvirus* genera available in GenBank (as of January 16th, 2024). Nucleotide sequences were aligned based on amino acid information using MUSCLE v.3.8.425 within Aliview v.2019.^28^ ^,^ ^29^ Regions of the alignment containing 10% or more gaps were removed with TrimAl v1.4.rev15.^30^ Maximum likelihood phylogeny (MLP) was inferred using IQ-TREE 2.1.4-beta, with the best-fit model determined by ModelFinder.^31^ ^,^ ^32^ Node support was calculated using the SH-like approximate likelihood ratio test (SH-aLRT) and Ultrafast Bootstrap (UFBoot).^33^ ^,^ ^34^ The phylogenetic tree was visualised using ggtree v.3.10 in R v.4.3.2.^35^ Identity matrices of nucleotide and amino acid sequences were computed using Bio3D v.2.4-4.^36^ A comprehensive list of the utilised sequences, along with the described alignment and tree, is available in Supplementary data (Table A-B). Identity matrices of nucleotides and amino acids can be found in the mentioned Supplementary data. Accession numbers are pending and will be provided during the review process prior to publication.

## RESULTS

*Hantavirus detection* - Screening of 97 samples resulted in the amplification of a partial hantaviral L-segment (412 bp) in lung samples from five frugivorous bats, *C. perspicillata* (GenBank accession number PP258966, PP258967, PP258968, PP258969 and PP258970), comprising 20 individuals (25%). Only one kidney sample yielded successful amplification. Among the samples, three animals were male (60%) and two were female (40%). No positive RT-PCR results were obtained for the other species investigated, including abundant species such as *Artibeus cinereus*, *A. planirostris*, and *A. lituratus* (Table I).

**TABLE I:** Species abundance of bats collected in the Guaribas Biological Reserve, Brazil

Family	Species	Abundance	Individuals tested by RT-PCR
Phyllostomidae	*Artibeus cinereus*	39	19
	*Artibeus lituratus*	19	17
	*Artibeus planirostris*	26	22
	*Carollia perspicillata*	90	20
	*Desmodus rotundus*	02	02
	*Glossophaga soricina*	12	11
	*Platyrrhinus lineatus*	08	03
Vespertilionidae	*Myotis* sp.	03	03
	Total	199	97

RT-PCR: reverse transcription-polymerase chain reaction.

Viral metagenomics yielded a total of 2.212.312 reads. After size and quality filtering, 1,948,462 reads were aligned against the host genome, leaving 453,483 reads for de novo assembly and taxonomic identification. Among the 106,977 reads identified, 855 were recognised as viruses (0.8%), including 10 reads belonging to the *Hantaviridae* family. De novo assembly generated 73,769 contigs (ranging from 55 to 16,060 nucleotides), with three contigs identified as *Hantaviridae*. Partial L segment fragment (260nt; PQ389507.1) and two M segment fragments (255 nt and 237 nt; PQ412688) showing 87.40% and 83% of identity with Buritiense virus RNA-dependent RNA polymerase gene (OR684449) and Xuan Son virus glycoprotein gene (KU976427.2), respectively. However, retrieval of the complete genome was not achievable via metagenomic analysis, likely due to various factors such as low virus titres in the examined organs, virus RNA degradation caused by the TRIzol^TM^ reagent (Invitrogen, San Diego, USA), tissue preservation methods, or the absence of viral enrichment steps in the sample processing during the viral metagenomics protocol.

*Genetic and phylogenetic analysis* - Pairwise alignment and comparison of a 412-nucleotide (138 amino acid) region of the L-segment revealed that the five *C. perspicillata* sequences analysed were highly similar, with nucleotide identity ranging from 94.9 to 99% and amino acid identity ranging from 91.7 to 99.2%. Comparative analyses of the L-partial sequences with other representative hantaviruses available in GenBank showed 55.6%-75% nucleotide sequence identity with other bat-borne hantaviruses, and most Soricomorpha (Table II). Sequences from Seba’s short-tailed bats exhibited < 76% (nt) and < 81.7% (aa) sequence similarity in the L-partial segment compared to all known hantaviruses, showing the highest degree of identity (76% nt and 81.7% aa) with Kiwira virus. However, these newly discovered strains are closely related to HantaV-1/hantavirus and Buritiense strains (84.2% - 89.4% nt and 91.5 - 100% aa identities) obtained from *Carollia* spp. collected in Bolivia and Brazil, based on partial L-segment sequences available in GenBank (Table II). These findings reveal a potentially novel bat-borne hantavirus, tentatively named MGPV.

**TABLE II:** Pairwise identity between amino acid and nucleotide partial sequences from Mamanguape virus and their closest relatives’ hantaviruses

Virus/%*	MAMANGUAPE PP258969.1	MAMANGUAPE PP258967.1	MAMANGUAPE PP258970.1	BURITIENSE	MOUYASSUÉ	KIWIRA	MAMANGUAPE PP258966.1	MAMANGUAPE PP258968.1	HNTV1_BOL	HNTV1_BRA	HNTV1_BRA	HNTV2_MEX	HNTV2_MEX	HNTV3_MEX	BRNO	LONGQUAN	QUEZON	ROBINA
MAMANGUAPE PP258969.1	-	99,3%	96,0%	87,3%	70,2%	76,0%	98,9%	98,9%	89,4%	87,8%	87,8%	84,0%	84,4%	72,2%	73,5%	73,5%	70,2%	64,7%
MAMANGUAPE PP258967.1	98,9%	-	94,9%	87,1%	69,4%	74,4%	99,2%	98,9%	88,0%	87,5%	87,5%	82,6%	82,3%	71,2%	74,4%	73,3%	71,9%	64,5%
MAMANGUAPE PP258970.1	95,6%	92,7%	-	84,2%	66,9%	72,8%	95,6%	95,3%	86,0%	84,3%	84,3%	79,5%	79,2%	68,9%	71,1%	71,1%	68,6%	63,6%
BURITIENSE	98,9%	99,2%	92,5%	-	70,5%	73,8%	87,5%	87,1%	85,8%	92,9%	93,4%	81,5%	80,6%	71,8%	73,0%	74,4%	68,9%	66,4%
MOUYASSUÉ	82,6%	80,2%	75,8%	80,2%	-	71,3%	70,3%	70,0%	68,4%	68,7%	69,2%	67,8%	68,1%	65,8%	71,6%	70,8%	67,5%	66,7%
KIWIRA	80,4%	81,0%	76,7%	81,8%	76,0%	-	75,0%	74,7%	72,9%	72,1%	71,8%	70,9%	70,9%	68,7%	71,9%	70,5%	71,1%	69,7%
MAMANGUAPE PP258966.1	98,9%	99,2%	92,5%	100,0%	80,0%	81,7%	-	99,7%	88,3%	87,7%	87,7%	82,6%	82,3%	71,8%	74,4%	72,8%	72,0%	64,4%
MAMANGUAPE PP258968.1	98,9%	98,3%	91,7%	99,2%	80,2%	81,0%	99,2%	-	88,0%	87,5%	87,5%	82,3%	82,1%	71,8%	74,1%	72,5%	71,7%	64,2%
HNTV1_BOL	98,9%	100,0%	93,2%	99,1%	79,5%	80,3%	99,1%	98,3%	-	87,5%	86,9%	82,6%	82,3%	73,5%	71,2%	74,4%	67,2%	67,5%
HNTV1_BRA	97,7%	98,3%	91,5%	99,1%	80,3%	82,1%	99,1%	98,3%	98,3%	-	98,9%	80,6%	79,8%	73,2%	73,8%	72,4%	69,0%	65,5%
HNTV1_BRA	97,7%	98,3%	91,5%	99,1%	80,3%	82,1%	99,1%	98,3%	98,3%	100,0%	-	79,8%	79,5%	72,9%	73,8%	72,9%	68,7%	65,5%
HNTV2_MEX	96,6%	96,6%	90,6%	97,4%	81,2%	80,3%	97,4%	96,6%	96,6%	96,6%	96,6%	-	98,9%	72,4%	71,2%	70,9%	65,5%	65,2%
HNTV2_MEX	96,6%	94,9%	89,7%	95,7%	80,3%	79,5%	95,7%	94,9%	94,9%	94,9%	94,9%	98,3%	-	72,4%	70,9%	70,7%	65,2%	64,7%
HNTV3_MEX	81,8%	81,2%	76,9%	81,2%	76,1%	75,2%	81,2%	81,2%	81,2%	82,1%	82,1%	81,2%	80,3%	-	67,8%	68,9%	71,0%	66,4%
BRNO	78,3%	77,7%	73,3%	78,5%	79,3%	76,0%	78,3%	78,5%	76,9%	78,6%	78,6%	77,8%	76,9%	76,1%	-	77,1%	70,8%	67,8%
LONGQUAN	78,3%	77,7%	73,3%	76,9%	79,3%	71,9%	76,7%	76,9%	76,9%	76,9%	76,9%	75,2%	74,4%	76,9%	81,0%	-	66,9%	70,2%
QUEZON	75,8%	77,5%	72,3%	78,3%	78,3%	78,3%	78,2%	77,5%	76,7%	78,4%	78,4%	77,6%	77,6%	76,7%	76,7%	75,0%	-	67,8%
ROBINA	71,7%	73,6%	70,8%	72,7%	75,2%	76,9%	72,5%	71,9%	72,6%	72,6%	72,6%	71,8%	70,9%	74,4%	73,6%	71,9%	80,0%	-

*Nucleotide (above the diagonal) and amino acid (below the diagonal) sequences are presented with their respective percentage values. BOL: Bolivia; BRA: Brazil; MEX: Mexico.

**Fig. 2:**
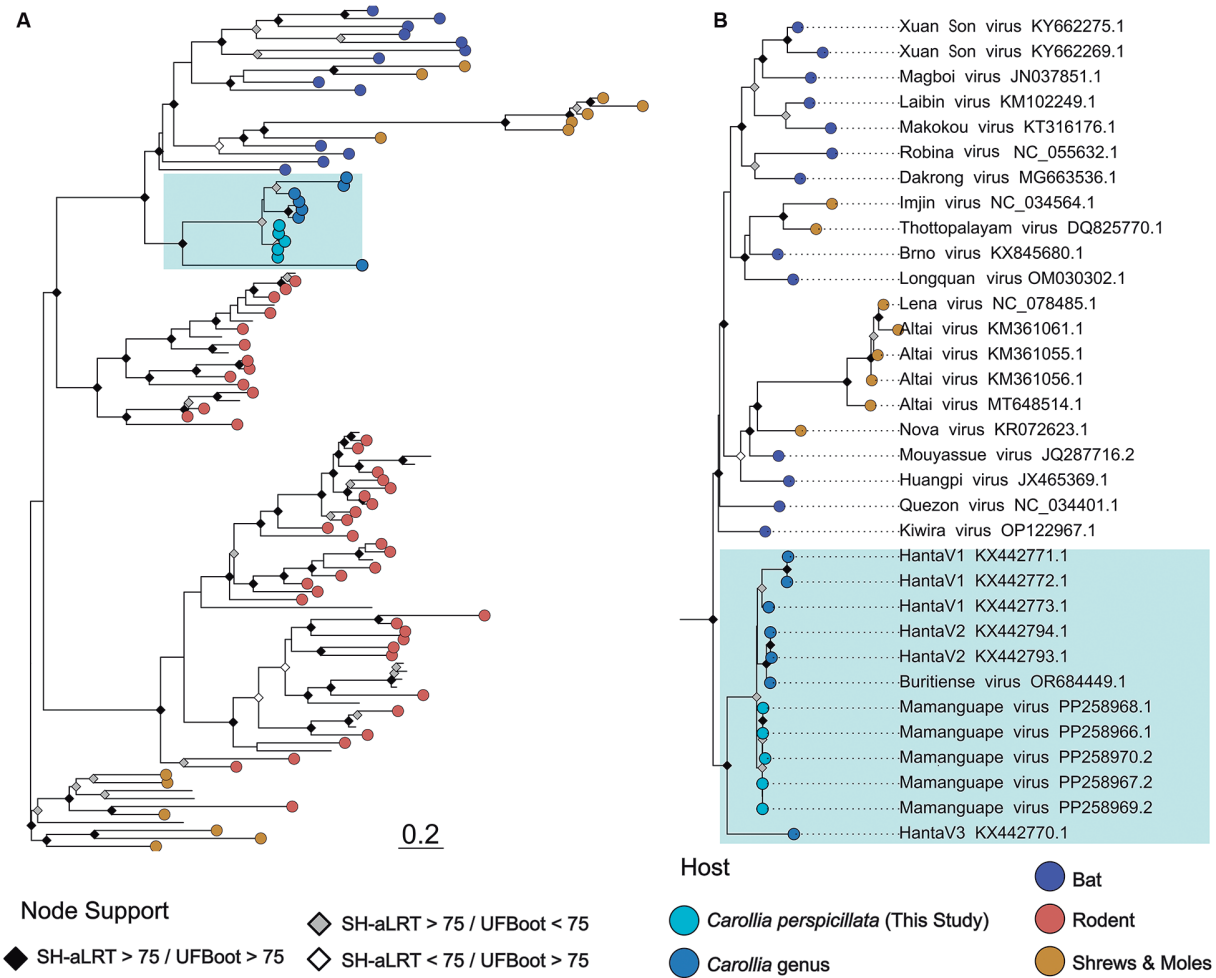
maximum likelihood tree illustrating the diversity of Hantaviruses, particularly focusing on novel sequences discovered in Seba’s short-tailed bats. The phylogeny inference was conducted using an alignment spanning 354 bp and comprising 106 sequences. The analysis employed the general time reversible model with invariable sites and gamma distribution with four rate categories (GTR+F+I+G4). To enhance visualisation, the tree was midpoint-rooted. Node supports are colour-coded, with black shapes indicating nodes with support ≥ 75 in SH-like approximate likelihood ratio test (SH-aLRT) and ultrafast bootstrap analyses, grey for SH-aLRT-only support, white for ultrafast bootstrap-only support, and unmarked nodes for support < 75 in both parameters. The tips of the tree are colour-coded based on known host information: light-blue represents *Carollia perspicillata* found in this study, dark blue indicates other known viruses found in the *Carollia* genus, purple denotes viruses found in other bats, red signifies rodents, and orange denotes shrews and moles. The grey-highlighted region within the tree represents the lineage of bat hantaviruses, with a magnified view provided on the right. Within this clade, the monophyletic *Carollia* hantavirus is highlighted in aqua-blue.

The MLP analysis based on partial L-segment sequences reveals that the sequences obtained from the five *C. perspicillata* (Mamanguape virus) specimens form a strongly monophyletic clade (SH-aLRT = 98.4, UFBoot = 95), closely related to strains from other *Carollia* samples, particularly Buritiense (Fig. 2). Notably, this clade is distinct and does not fall within other known clades of bat Hantaviruses. The Mamanguape virus sequences exhibit significant genetic divergence from other bat-, insectivore, -and rodent-borne hantaviruses. Despite the inability to amplify the complete genome, the partial genetic and phylogenetic data suggest that the newly discovered virus strain, designated as Mamanguape, likely represents a distinct lineage within the class of bat-borne hantaviruses.

## DISCUSSION

In this study, we identified a distinct bat-borne hantavirus from a bat reservoir in Brazil, *C. perspicillata*, captured in the *Guaribas Biological Reserve*, located in the Atlantic Rainforest biome in the State of Paraíba. Seba’s short-tailed bat (*C. perspicillata*) has an extensive geographic distribution spanning from Mexico throughout tropical South America, including all biomes of Brazil,^37^ ^,^ ^38^ and extending southwards to Paraguay and southern Bolivia.^39^ This primarily frugivorous bat thrives in tropical lowland humid forests but also adapts well to human-modified landscapes. It is abundant in semideciduous tropical forests surrounded by sugarcane monoculture, such as the Guaribas Biological Reserve, as well as in urban forest fragments and human settlements showcasing its behavioural plasticity.^40^ ^,^ ^41^ In such contexts, the most common species often serve as competent hosts for pathogen transmission, potentially altering the dynamics of transmission of pathogens that cause known endemic diseases.^42^ The presence of bats in urban areas increases the probability of contact with humans and domestic animals, particularly cats. As observed with the rabies virus,^43^ this interaction can amplify or alter the pathogen transmission cycle.^44^

To date, there have been no reports of bat-borne hantaviruses associated with human cases, although the potential spillover of the MGPV from *C. perspicillata* to humans warrants consideration due to several factors: (1) their habitat associations with disturbed areas, (2) their opportunistic and generalist habits, (3) their tendency to roost in urban areas, (4) their high abundance, and (5) the high hantavirus prevalence of *C. perspicillata* (25%) reported in the study area. In this context, comparative sequence analysis of hantavirus patients and bat-borne hantaviruses described in the same area should help clarify the implications, if any, of these newfound hantaviruses as etiologic agents for human disease in Brazil. Su et al. emphasise that a bat airway organoid system (culture model) might be a useful tool to analyse such questions,^45^ including virus infectivity and interspecies transmission. Moreover, specific serological assays using bat-borne hantaviruses as antigens, virus neutralisation tests using bat-borne hantavirus, and the surveillance of patients with an epidemiological history of contact with bats (exposed subjects) should help clarify the impact of these hantaviruses on human health.

According to our data, five *C. perspicillata* individuals tested positive for hantavirus, of which three (60%) were males, all adults, including a lactating female. Studies on rodent-borne hantaviruses suggest that older individuals have a higher likelihood of infection due to longer exposure periods to the virus.^46^ ^,^ ^47^ ^,^ ^48^ Additionally, dominant males of *C. perspicillata*, are known to have harems of ten or more females, exhibit behaviours conducting to horizontal viral transmission, such as gregarious and grooming behaviours. This behaviour facilitates transmission via aerosolised excreta and secretions, the primary route of hantavirus transmission among.^49^ ^,^ ^50^ The detection of hantavirus RNA in the kidney of one specimen further suggests a possible excretion route. Our study found hantavirus exclusively in *C. perspicillata* individuals, indicating potential host specificity. Similar host specificity has been suggested in studies conducted in Europe and Asia involving other bat (*Nyctalus noctula:* Vespertilionidae) and QZNV and Geoffroy’s rousettes (*Rousettus amplexicaudatus*: Pteropodidae) species.^8^ ^,^ ^51^ Further ecological studies with continuous long-term data collection are necessary to elucidate the role of other bat species and enhance our understanding of bat-borne hantavirus ecology in Brazil.

**Fig. 3:**
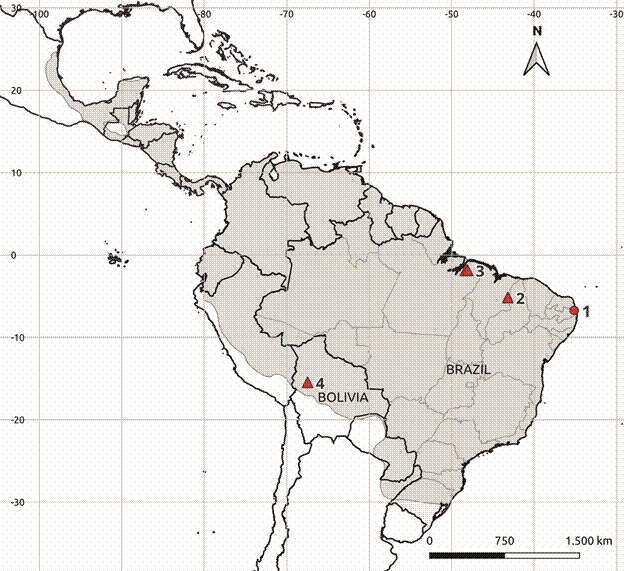
distribution map of *Carollia perspicillata* in the Americas in grey colour.^38^ Circle 1 represents the sequences of collection points of *C. perspicillata* samples positive for hantavirus which were included in this study. Triangles 2 represents the Buritiense strain (OR684449.1), 3 and 4 represent the areas where other hantaviruses were also detected in *C. perspicillata* (unpublished data)*.*

The MGPV sequences of *C. perspicillata* shared 68.9-89.4% and 76.9-100% identities at the nucleotide and amino acid levels, respectively, with *Carollia*-associated sequences (data only available at GenBank: KX442773; KX442793; KX442794; KX442772; KX442771; KX442770; OR684449.1), based on the L (412 nt) segment. This similarity was expected as MGPV, Hanta-V and Buritiense strains belong to the same bat genera, supporting the cospeciation hypothesis and indicating stable circulation of Mamanguape viruses in *C. perspicillata* bats across their geographic distribution (Fig. 3). However, compared to well-recognised bat-borne hantaviruses, Brazilian sequences showed the highest similarity (approximately 81% aa) with Kiwira virus recently described in Free Tailed bat (*Mops condylurus*) from East and Central Africa.^8^ ^,^ ^9^ ^,^ ^52^

Phylogenetic analysis placed the MGPV and *Carollia*-associated sequences in a well-supported monophyletic group, suggesting a highly divergent lineage composed of Brazilian and potentially South American bats. The unsuccessful attempt to obtain the complete genome amplification of MGPV may be attributed to several factors, including the high diversity of bat-borne hantavirus sequences, low virus titre, or RNA degradation due to improper tissue preservation, as demonstrated in previous related studies.^53^ ^,^ ^54^ ^,^ ^55^ ^,^ ^56^ ^,^ ^57^ Our ongoing efforts involve complete sequencing and phylogenetic analyses of the L segment, as well as the S and M segments, to better characterise MGPV sequences. Based on Pairwise Evolutionary Distance values (calculated from the complete concatenated sequences), we aim to determine its taxonomic classification (species or new genera within the subfamily *Mammantavirinae*) according to the current international committee on taxonomy of viruses guidelines. Future specimen collection in follow-up studies within the same region, along with molecular approaches such as genome walking, will not only address this issue but also enhance our understanding of the ecology and virus-host interactions of MGPV. In summary, we present a presumably novel hantavirus provisionally named MGPV, detected in Seba’s short-tailed bat (*C. perspicillata*, Phyllostomidae) from Brazil. Further analyses are underway, but our results suggest that the described sequences represent a novel hantavirus, with Seba’s short-tailed bat *C. perspicillata* serving as the natural host for this virus, which is potentially distributed throughout South America.

*Ethics issues* - The study was conducted in accordance with the Declaration of Helsinki, and approved by the institutional Ethics Committee on Animal Research, the Ethics Committee of the Oswaldo Cruz Foundation (FIOCRUZ) CEUA LW-81/12, P-42 /12.1. Permits for field collection were granted by IBAMA/Sisbio (Brazilian Institute of Environment and Renewable Natural Resources) under process number IBAMA/Sisbio No. 41683.
