# Supplementary material for: A newly bat-borne hantavirus detected in Seba’s short-tailed bats (Carollia perspicillata) in the Brazilian Atlantic Rainforest
Source: Mem Inst Oswaldo Cruz. 2024 Dec 16;119:e240132. doi: 10.1590/0074-02760240132 (PMC11654739; doi:10.1590/0074-02760240132)
Supplement: Supplementary file 1 [file 1678-8060-mioc-119-e240132-s.pdf]

TABLE A

Percent identity matrix based on amino acids

	Mamanguape_virus_EM_725	Mamanguape_virus_EM_711	Mamanguape_virus_EM_708	OR684449.1	MG663536.1	JN037851.1	KT316176.1	JQ287716.2	OP122967.1	JX465369.1	Mamanguape_virus_EM_709	Mamanguape_virus_EM_707	Hantavirus_HantaV1_KX442773_1	Hantavirus_HantaV1_KX442793_1	Hantavirus_HantaV1_KX442794_1	Hantavirus_HantaV2_KX442772_1	Hantavirus_HantaV2_KX442771_1	Hantavirus_HantaV3_KX442770_1	EF405801.1	NC_005217.1	NC_005226.1	NC_006435.1	NC_055170.1	AF291704.5	AY526217.1	DQ371906.1	DQ825770.1
Mamanguape_virus_EM_725	1,00	0,99	0,96	0,99	0,75	0,69	0,70	0,83	0,80	0,82	0,99	0,99	0,99	0,98	0,98	0,97	0,97	0,82	0,64	0,64	0,66	0,66	0,58	0,67	0,63	0,67	0,73
Mamanguape_virus_EM_711	0,99	1,00	0,93	0,99	0,76	0,73	0,74	0,80	0,81	0,80	0,99	0,98	1,00	0,98	0,98	0,97	0,95	0,81	0,67	0,65	0,66	0,67	0,59	0,66	0,66	0,68	0,74
Mamanguape_virus_EM_708	0,96	0,93	1,00	0,93	0,72	0,69	0,71	0,76	0,77	0,77	0,93	0,92	0,93	0,92	0,92	0,91	0,90	0,77	0,64	0,61	0,62	0,64	0,56	0,63	0,63	0,64	0,70
OR684449.1	0,99	0,99	0,93	1,00	0,76	0,74	0,74	0,80	0,82	0,80	1,00	0,99	0,99	0,99	0,99	0,97	0,96	0,81	0,66	0,64	0,66	0,67	0,60	0,66	0,66	0,68	0,74
MG663536.1	0,75	0,76	0,72	0,76	1,00	0,74	0,78	0,78	0,75	0,78	0,76	0,75	0,75	0,76	0,76	0,74	0,74	0,74	0,66	0,64	0,65	0,68	0,60	0,66	0,64	0,68	0,76
JN037851.1	0,69	0,73	0,69	0,74	0,74	1,00	0,79	0,74	0,72	0,73	0,73	0,73	0,72	0,74	0,74	0,73	0,71	0,72	0,65	0,63	0,66	0,63	0,61	0,66	0,63	0,62	0,77
KT316176.1	0,70	0,74	0,71	0,74	0,78	0,79	1,00	0,76	0,76	0,76	0,74	0,73	0,74	0,74	0,74	0,74	0,74	0,74	0,63	0,61	0,65	0,67	0,62	0,65	0,62	0,66	0,73
JQ287716.2	0,83	0,80	0,76	0,80	0,78	0,74	0,76	1,00	0,76	0,83	0,80	0,80	0,80	0,80	0,80	0,81	0,80	0,76	0,68	0,66	0,68	0,69	0,63	0,70	0,66	0,70	0,76
OP122967.1	0,80	0,81	0,77	0,82	0,75	0,72	0,76	0,76	1,00	0,77	0,82	0,81	0,80	0,82	0,82	0,80	0,80	0,75	0,66	0,67	0,67	0,68	0,60	0,68	0,66	0,66	0,69
JX465369.1	0,82	0,80	0,77	0,80	0,78	0,73	0,76	0,83	0,77	1,00	0,79	0,80	0,79	0,80	0,80	0,77	0,77	0,75	0,62	0,61	0,61	0,66	0,61	0,66	0,63	0,66	0,75
Mamanguape_virus_EM_709	0,99	0,99	0,93	1,00	0,76	0,73	0,74	0,80	0,82	0,79	1,00	0,99	0,99	0,99	0,99	0,97	0,96	0,81	0,66	0,64	0,66	0,67	0,59	0,66	0,65	0,68	0,74
Mamanguape_virus_EM_707	0,99	0,98	0,92	0,99	0,75	0,73	0,73	0,80	0,81	0,80	0,99	1,00	0,98	0,98	0,98	0,97	0,95	0,81	0,66	0,64	0,66	0,67	0,60	0,66	0,66	0,68	0,74
Hantavirus_HantaV1_KX442773_1	0,99	1,00	0,93	0,99	0,75	0,72	0,74	0,80	0,80	0,79	0,99	0,98	1,00	0,98	0,98	0,97	0,95	0,81	0,66	0,64	0,64	0,66	0,57	0,65	0,65	0,67	0,74
Hantavirus_HantaV1_KX442793_1	0,98	0,98	0,92	0,99	0,76	0,74	0,74	0,80	0,82	0,80	0,99	0,98	0,98	1,00	1,00	0,97	0,95	0,82	0,64	0,62	0,64	0,65	0,59	0,64	0,64	0,66	0,74
Hantavirus_HantaV1_KX442794_1	0,98	0,98	0,92	0,99	0,76	0,74	0,74	0,80	0,82	0,80	0,99	0,98	0,98	1,00	1,00	0,97	0,95	0,82	0,64	0,62	0,64	0,65	0,59	0,64	0,64	0,66	0,74
Hantavirus_HantaV2_KX442772_1	0,97	0,97	0,91	0,97	0,74	0,73	0,74	0,81	0,80	0,77	0,97	0,97	0,97	0,97	0,97	1,00	0,98	0,81	0,65	0,64	0,65	0,67	0,58	0,65	0,64	0,68	0,74
Hantavirus_HantaV2_KX442771_1	0,97	0,95	0,90	0,96	0,74	0,71	0,74	0,80	0,80	0,77	0,96	0,95	0,95	0,95	0,95	0,98	1,00	0,80	0,64	0,63	0,64	0,67	0,57	0,64	0,64	0,67	0,73
Hantavirus_HantaV3_KX442770_1	0,82	0,81	0,77	0,81	0,74	0,72	0,74	0,76	0,75	0,75	0,81	0,81	0,81	0,82	0,82	0,81	0,80	1,00	0,64	0,65	0,64	0,69	0,62	0,69	0,64	0,67	0,75
EF405801.1	0,64	0,67	0,64	0,66	0,66	0,65	0,63	0,68	0,66	0,62	0,66	0,66	0,66	0,64	0,64	0,65	0,64	0,64	1,00	0,79	0,86	0,67	0,63	0,81	0,96	0,68	0,64
NC_005217.1	0,64	0,65	0,61	0,64	0,64	0,63	0,61	0,66	0,67	0,61	0,64	0,64	0,64	0,62	0,62	0,64	0,63	0,65	0,79	1,00	0,80	0,67	0,62	0,86	0,77	0,67	0,63
NC_005226.1	0,66	0,66	0,62	0,66	0,65	0,66	0,65	0,68	0,67	0,61	0,66	0,66	0,64	0,64	0,64	0,65	0,64	0,64	0,86	0,80	1,00	0,67	0,66	0,81	0,84	0,69	0,63
NC_006435.1	0,66	0,67	0,64	0,67	0,68	0,63	0,67	0,69	0,68	0,66	0,67	0,67	0,66	0,65	0,65	0,67	0,67	0,69	0,67	0,67	0,67	1,00	0,66	0,68	0,66	0,96	0,67
NC_055170.1	0,58	0,59	0,56	0,60	0,60	0,61	0,62	0,63	0,60	0,61	0,59	0,60	0,57	0,59	0,59	0,58	0,57	0,62	0,63	0,62	0,66	0,66	1,00	0,66	0,63	0,66	0,67
AF291704.5	0,67	0,66	0,63	0,66	0,66	0,66	0,65	0,70	0,68	0,66	0,66	0,66	0,65	0,64	0,64	0,65	0,64	0,69	0,81	0,86	0,81	0,68	0,66	1,00	0,77	0,66	0,66
AY526217.1	0,63	0,66	0,63	0,66	0,64	0,63	0,62	0,66	0,66	0,63	0,65	0,66	0,65	0,64	0,64	0,64	0,64	0,64	0,96	0,77	0,84	0,66	0,63	0,77	1,00	0,67	0,64
DQ371906.1	0,67	0,68	0,64	0,68	0,68	0,62	0,66	0,70	0,66	0,66	0,68	0,68	0,67	0,66	0,66	0,68	0,67	0,67	0,68	0,67	0,69	0,96	0,66	0,66	0,67	1,00	0,67
DQ825770.1	0,73	0,74	0,70	0,74	0,76	0,77	0,73	0,76	0,69	0,75	0,74	0,74	0,74	0,74	0,74	0,74	0,73	0,75	0,64	0,63	0,63	0,67	0,67	0,66	0,64	0,67	1,00
DQ989237.1	0,68	0,68	0,64	0,68	0,66	0,61	0,63	0,69	0,65	0,66	0,68	0,68	0,67	0,66	0,66	0,68	0,67	0,65	0,66	0,66	0,66	0,92	0,62	0,66	0,65	0,93	0,66
EF397003.1	0,66	0,66	0,62	0,66	0,66	0,68	0,64	0,68	0,69	0,66	0,65	0,66	0,64	0,65	0,65	0,64	0,64	0,70	0,78	0,86	0,80	0,66	0,66	0,89	0,77	0,66	0,69
EF646763.1	0,66	0,69	0,65	0,68	0,69	0,65	0,66	0,69	0,70	0,64	0,68	0,68	0,68	0,66	0,66	0,67	0,66	0,67	0,87	0,78	0,89	0,70	0,64	0,79	0,86	0,71	0,64
EU788002.1	0,66	0,66	0,62	0,66	0,66	0,66	0,62	0,65	0,68	0,64	0,65	0,66	0,64	0,64	0,64	0,64	0,64	0,67	0,77	0,87	0,81	0,64	0,62	0,88	0,77	0,63	0,65
GU140096.1	0,66	0,67	0,64	0,67	0,69	0,64	0,67	0,70	0,67	0,66	0,67	0,67	0,66	0,65	0,65	0,67	0,67	0,68	0,68	0,66	0,67	0,99	0,66	0,67	0,67	0,96	0,68

CC BY

FJ858378.1	0,67	0,69	0,65	0,68	0,67	0,70	0,66	0,70	0,69	0,66	0,68	0,68	0,68	0,66	0,66	0,67	0,66	0,70	0,82	0,83	0,81	0,69	0,66	0,87	0,80	0,70	0,69
GQ244526.1	0,67	0,68	0,64	0,67	0,65	0,69	0,66	0,69	0,70	0,68	0,67	0,67	0,67	0,65	0,65	0,66	0,66	0,70	0,80	0,83	0,78	0,70	0,65	0,87	0,78	0,69	0,68
HM015220.1	0,62	0,64	0,60	0,64	0,67	0,62	0,63	0,70	0,63	0,65	0,64	0,64	0,63	0,64	0,64	0,62	0,62	0,64	0,77	0,71	0,75	0,64	0,66	0,73	0,74	0,65	0,65
JQ026206.1	0,72	0,71	0,67	0,71	0,70	0,64	0,66	0,73	0,71	0,68	0,70	0,71	0,70	0,70	0,70	0,69	0,68	0,70	0,68	0,67	0,70	0,81	0,64	0,66	0,67	0,82	0,68
JQ083393.1	0,66	0,67	0,64	0,67	0,68	0,63	0,67	0,69	0,68	0,66	0,67	0,67	0,66	0,65	0,65	0,67	0,67	0,69	0,67	0,67	0,67	1,00	0,66	0,68	0,66	0,96	0,67
JN831945.1	0,64	0,67	0,64	0,66	0,66	0,64	0,62	0,67	0,66	0,63	0,66	0,66	0,66	0,64	0,64	0,65	0,64	0,64	0,99	0,78	0,85	0,67	0,64	0,80	0,97	0,68	0,65
JX853574.1	0,70	0,70	0,66	0,70	0,70	0,65	0,67	0,72	0,72	0,69	0,70	0,70	0,69	0,70	0,70	0,69	0,68	0,72	0,67	0,71	0,71	0,82	0,64	0,68	0,66	0,82	0,67
JX879770.1	0,70	0,70	0,66	0,70	0,70	0,65	0,67	0,72	0,72	0,69	0,70	0,70	0,69	0,70	0,70	0,69	0,68	0,72	0,67	0,71	0,71	0,82	0,64	0,68	0,66	0,82	0,67
JX028271.1	0,66	0,67	0,64	0,66	0,66	0,65	0,64	0,70	0,67	0,63	0,66	0,66	0,66	0,64	0,64	0,65	0,64	0,67	0,95	0,79	0,87	0,67	0,63	0,79	0,93	0,67	0,66
KF705679.1	0,63	0,65	0,63	0,64	0,64	0,64	0,66	0,63	0,66	0,63	0,64	0,64	0,66	0,65	0,65	0,65	0,65	0,69	0,66	0,64	0,65	0,76	0,64	0,66	0,66	0,75	0,61
KF537001.1	0,64	0,65	0,61	0,64	0,64	0,63	0,61	0,66	0,67	0,61	0,64	0,64	0,64	0,62	0,62	0,64	0,63	0,65	0,79	1,00	0,80	0,67	0,62	0,86	0,77	0,67	0,63
KM102249.1	0,75	0,76	0,72	0,76	0,83	0,81	0,86	0,78	0,77	0,80	0,76	0,75	0,75	0,76	0,76	0,74	0,74	0,73	0,69	0,65	0,68	0,68	0,61	0,66	0,67	0,69	0,74
KR072623.1	0,82	0,79	0,75	0,79	0,73	0,71	0,67	0,81	0,74	0,79	0,79	0,79	0,78	0,80	0,80	0,79	0,78	0,74	0,62	0,62	0,65	0,63	0,62	0,63	0,63	0,64	0,76
KM361055.1	0,66	0,69	0,66	0,69	0,68	0,64	0,68	0,73	0,65	0,70	0,68	0,69	0,68	0,68	0,68	0,68	0,67	0,68	0,63	0,62	0,62	0,64	0,60	0,61	0,62	0,64	0,69
KM361056.1	0,66	0,69	0,66	0,69	0,68	0,64	0,68	0,73	0,65	0,70	0,68	0,69	0,68	0,68	0,68	0,68	0,67	0,68	0,63	0,62	0,62	0,64	0,60	0,61	0,62	0,64	0,69
KM361061.1	0,66	0,69	0,66	0,69	0,68	0,64	0,67	0,73	0,65	0,71	0,68	0,69	0,68	0,68	0,68	0,68	0,67	0,68	0,62	0,61	0,61	0,63	0,60	0,60	0,63	0,63	0,69
KT885041.1	0,72	0,71	0,67	0,71	0,70	0,63	0,65	0,73	0,71	0,68	0,70	0,71	0,70	0,70	0,70	0,69	0,68	0,70	0,67	0,68	0,68	0,81	0,63	0,67	0,66	0,81	0,66
KT885044.1	0,64	0,65	0,61	0,64	0,64	0,63	0,61	0,66	0,67	0,61	0,64	0,64	0,64	0,62	0,62	0,64	0,63	0,65	0,79	1,00	0,80	0,67	0,62	0,86	0,77	0,67	0,63
KT885047.1	0,66	0,67	0,64	0,67	0,68	0,63	0,67	0,69	0,68	0,66	0,67	0,67	0,66	0,65	0,65	0,67	0,67	0,69	0,67	0,67	0,67	0,99	0,66	0,68	0,66	0,96	0,67
KT885050.1	0,64	0,67	0,64	0,66	0,66	0,65	0,63	0,68	0,66	0,62	0,66	0,66	0,66	0,64	0,64	0,65	0,64	0,64	1,00	0,79	0,86	0,67	0,63	0,81	0,96	0,68	0,64
KT934965.1	0,66	0,67	0,64	0,67	0,68	0,63	0,67	0,69	0,68	0,66	0,67	0,67	0,66	0,65	0,65	0,67	0,67	0,69	0,67	0,67	0,67	0,99	0,66	0,68	0,66	0,96	0,67
KU215675.1	0,66	0,67	0,64	0,67	0,69	0,64	0,67	0,70	0,67	0,66	0,67	0,67	0,66	0,65	0,65	0,67	0,67	0,68	0,68	0,66	0,67	0,99	0,66	0,67	0,67	0,96	0,68
KX845680.1	0,78	0,78	0,73	0,79	0,75	0,76	0,72	0,79	0,76	0,80	0,78	0,79	0,77	0,79	0,79	0,78	0,77	0,76	0,66	0,66	0,66	0,68	0,63	0,68	0,65	0,68	0,82
KY594712.1	0,66	0,67	0,64	0,67	0,68	0,63	0,67	0,69	0,68	0,66	0,67	0,67	0,66	0,65	0,65	0,67	0,67	0,69	0,67	0,67	0,67	1,00	0,66	0,68	0,66	0,96	0,67
KY662269.1	0,67	0,71	0,67	0,70	0,77	0,79	0,78	0,76	0,74	0,70	0,70	0,69	0,70	0,70	0,70	0,70	0,69	0,69	0,69	0,69	0,68	0,64	0,62	0,68	0,66	0,66	0,74
KY662275.1	0,70	0,74	0,69	0,73	0,78	0,75	0,79	0,77	0,77	0,71	0,73	0,72	0,73	0,73	0,73	0,73	0,72	0,68	0,69	0,67	0,67	0,65	0,62	0,68	0,66	0,66	0,73
MG717393.1	0,64	0,66	0,62	0,65	0,64	0,64	0,62	0,67	0,70	0,64	0,64	0,65	0,64	0,64	0,64	0,64	0,64	0,67	0,80	0,95	0,78	0,70	0,65	0,87	0,77	0,70	0,66
KY978757.1	0,67	0,67	0,64	0,67	0,67	0,61	0,65	0,67	0,67	0,66	0,67	0,67	0,66	0,65	0,65	0,65	0,65	0,68	0,66	0,66	0,66	0,98	0,65	0,68	0,65	0,93	0,65
MN183135.1	0,66	0,66	0,63	0,67	0,66	0,64	0,66	0,68	0,68	0,62	0,67	0,67	0,65	0,65	0,65	0,66	0,65	0,64	0,87	0,80	0,97	0,69	0,66	0,82	0,86	0,70	0,62
MN850093.1	0,67	0,66	0,63	0,66	0,66	0,67	0,65	0,70	0,68	0,66	0,66	0,66	0,65	0,64	0,64	0,65	0,64	0,69	0,81	0,86	0,82	0,68	0,66	0,99	0,77	0,66	0,66
MN850096.1	0,67	0,66	0,63	0,66	0,66	0,67	0,65	0,70	0,68	0,66	0,66	0,66	0,65	0,64	0,64	0,65	0,64	0,69	0,82	0,86	0,82	0,68	0,66	0,98	0,78	0,66	0,66
MN639737.1	0,63	0,66	0,63	0,66	0,68	0,63	0,62	0,66	0,66	0,62	0,65	0,66	0,65	0,64	0,64	0,64	0,64	0,64	0,98	0,80	0,83	0,66	0,63	0,79	0,95	0,66	0,64
MN639740.1	0,63	0,66	0,63	0,66	0,68	0,63	0,62	0,66	0,66	0,62	0,65	0,66	0,65	0,64	0,64	0,64	0,64	0,64	0,98	0,80	0,83	0,66	0,63	0,79	0,95	0,66	0,64
MT024592.1	0,64	0,67	0,64	0,66	0,66	0,64	0,62	0,67	0,66	0,63	0,66	0,66	0,66	0,64	0,64	0,65	0,64	0,64	0,99	0,78	0,85	0,67	0,64	0,80	0,97	0,68	0,65
MT514291.1	0,63	0,66	0,63	0,66	0,68	0,64	0,62	0,66	0,66	0,61	0,65	0,66	0,65	0,64	0,64	0,64	0,64	0,64	0,98	0,81	0,84	0,66	0,62	0,80	0,94	0,66	0,63
MT514292.1	0,64	0,67	0,64	0,66	0,67	0,63	0,62	0,65	0,66	0,62	0,66	0,66	0,66	0,64	0,64	0,65	0,64	0,66	0,94	0,79	0,84	0,66	0,63	0,78	0,93	0,66	0,64
MT514294.1	0,64	0,67	0,64	0,66	0,66	0,64	0,62	0,67	0,66	0,63	0,66	0,66	0,66	0,64	0,64	0,65	0,64	0,64	0,99	0,78	0,85	0,67	0,64	0,80	0,97	0,68	0,65
MT514295.1	0,66	0,66	0,62	0,66	0,65	0,66	0,65	0,68	0,67	0,61	0,66	0,66	0,64	0,64	0,64	0,65	0,64	0,64	0,86	0,80	1,00	0,67	0,66	0,81	0,84	0,69	0,63
MT514296.1	0,66	0,66	0,63	0,67	0,66	0,64	0,66	0,68	0,68	0,62	0,67	0,67	0,65	0,65	0,65	0,66	0,65	0,64	0,87	0,80	0,98	0,69	0,66	0,82	0,86	0,70	0,62
MT514297.1	0,67	0,67	0,64	0,68	0,67	0,66	0,66	0,69	0,70	0,63	0,68	0,68	0,66	0,66	0,66	0,67	0,66	0,65	0,86	0,81	0,96	0,69	0,66	0,82	0,84	0,68	0,63
MT514298.1	0,66	0,66	0,62	0,66	0,65	0,66	0,65	0,68	0,67	0,61	0,66	0,66	0,64	0,64	0,64	0,65	0,64	0,64	0,86	0,80	1,00	0,67	0,66	0,81	0,84	0,69	0,63
MN258157.1	0,67	0,66	0,63	0,66	0,66	0,67	0,65	0,70	0,68	0,66	0,66	0,66	0,65	0,64	0,64	0,65	0,64	0,69	0,81	0,86	0,82	0,68	0,66	0,99	0,77	0,66	0,66
MN832779.1	0,64	0,67	0,64	0,66	0,65	0,64	0,62	0,66	0,66	0,64	0,66	0,66	0,66	0,64	0,64	0,65	0,64	0,64	0,97	0,77	0,85	0,67	0,64	0,78	0,99	0,68	0,65
MN832782.1	0,64	0,67	0,64	0,66	0,66	0,64	0,62	0,67	0,66	0,63	0,66	0,66	0,66	0,64	0,64	0,65	0,64	0,64	0,99	0,78	0,85	0,67	0,64	0,80	0,97	0,68	0,65

MT648514.1	0,69	0,70	0,67	0,69	0,65	0,69	0,66	0,69	0,65	0,68	0,69	0,69	0,69	0,69	0,69	0,68	0,66	0,69	0,60	0,60	0,61	0,61	0,59	0,61	0,58	0,61	0,69
OM030302.1	0,78	0,78	0,73	0,77	0,73	0,73	0,71	0,79	0,72	0,76	0,77	0,77	0,77	0,77	0,77	0,75	0,74	0,77	0,68	0,67	0,68	0,68	0,65	0,67	0,66	0,69	0,78
OM030305.1	0,69	0,70	0,66	0,70	0,68	0,62	0,65	0,71	0,66	0,67	0,70	0,70	0,69	0,68	0,68	0,70	0,69	0,67	0,67	0,66	0,66	0,94	0,63	0,66	0,66	0,96	0,66
OM030311.1	0,64	0,66	0,64	0,66	0,68	0,64	0,65	0,66	0,69	0,64	0,65	0,66	0,65	0,65	0,65	0,64	0,64	0,66	0,68	0,67	0,66	0,79	0,65	0,67	0,68	0,78	0,62
OK422869.1	0,71	0,70	0,68	0,70	0,69	0,64	0,66	0,72	0,70	0,67	0,70	0,70	0,69	0,70	0,70	0,68	0,67	0,69	0,66	0,67	0,67	0,80	0,62	0,66	0,66	0,80	0,66
MZ504241.1	0,71	0,71	0,67	0,71	0,70	0,65	0,68	0,74	0,72	0,70	0,70	0,71	0,70	0,70	0,70	0,70	0,70	0,71	0,66	0,71	0,70	0,82	0,64	0,67	0,66	0,83	0,69
OQ092243.1	0,64	0,64	0,60	0,64	0,65	0,64	0,62	0,68	0,66	0,66	0,64	0,64	0,63	0,62	0,62	0,63	0,62	0,66	0,78	0,86	0,78	0,66	0,63	0,93	0,77	0,64	0,65
OR148904.1	0,69	0,68	0,64	0,67	0,67	0,66	0,63	0,68	0,68	0,62	0,67	0,67	0,67	0,65	0,65	0,66	0,65	0,69	0,81	0,89	0,82	0,69	0,64	0,87	0,78	0,67	0,66
OR365538.1	0,50	0,52	0,52	0,52	0,52	0,54	0,51	0,54	0,58	0,55	0,52	0,52	0,50	0,51	0,51	0,50	0,50	0,56	0,63	0,70	0,64	0,55	0,53	0,72	0,61	0,54	0,56
NC_005235.1	0,72	0,71	0,67	0,71	0,70	0,63	0,65	0,73	0,71	0,68	0,70	0,71	0,70	0,70	0,70	0,69	0,68	0,70	0,67	0,68	0,68	0,81	0,63	0,67	0,66	0,81	0,66
NC_034401.1	0,76	0,78	0,72	0,78	0,79	0,71	0,75	0,78	0,78	0,75	0,78	0,78	0,77	0,78	0,78	0,78	0,78	0,77	0,69	0,64	0,66	0,69	0,63	0,66	0,66	0,68	0,75
NC_034399.1	0,68	0,70	0,68	0,71	0,68	0,66	0,69	0,71	0,74	0,66	0,70	0,71	0,69	0,69	0,69	0,70	0,69	0,68	0,70	0,66	0,68	0,77	0,68	0,69	0,68	0,77	0,68
NC_034402.1	0,66	0,66	0,62	0,66	0,67	0,66	0,68	0,69	0,66	0,70	0,65	0,66	0,64	0,65	0,65	0,64	0,64	0,67	0,66	0,68	0,68	0,70	0,69	0,69	0,66	0,70	0,70
NC_034403.1	0,66	0,66	0,63	0,66	0,65	0,61	0,61	0,66	0,64	0,61	0,65	0,66	0,65	0,64	0,64	0,64	0,64	0,67	0,82	0,85	0,82	0,71	0,66	0,85	0,82	0,74	0,65
NC_034407.1	0,64	0,68	0,64	0,67	0,70	0,67	0,70	0,67	0,70	0,67	0,67	0,67	0,67	0,67	0,67	0,66	0,65	0,68	0,72	0,67	0,70	0,72	0,67	0,68	0,72	0,72	0,72
NC_034467.1	0,68	0,70	0,66	0,69	0,65	0,63	0,65	0,69	0,70	0,64	0,69	0,69	0,69	0,67	0,67	0,68	0,67	0,69	0,81	0,79	0,80	0,73	0,66	0,81	0,81	0,72	0,66
NC_034485.1	0,68	0,68	0,66	0,67	0,69	0,66	0,68	0,67	0,69	0,65	0,67	0,67	0,67	0,66	0,66	0,66	0,65	0,69	0,69	0,69	0,69	0,81	0,66	0,70	0,68	0,80	0,65
NC_034515.1	0,67	0,67	0,64	0,66	0,65	0,65	0,62	0,68	0,68	0,62	0,66	0,66	0,66	0,64	0,64	0,65	0,64	0,67	0,81	0,88	0,81	0,66	0,63	0,87	0,79	0,66	0,65
NC_034517.1	0,71	0,71	0,68	0,71	0,70	0,66	0,66	0,72	0,72	0,68	0,70	0,71	0,70	0,70	0,70	0,70	0,70	0,71	0,68	0,70	0,69	0,84	0,66	0,69	0,67	0,82	0,71
NC_034519.1	0,67	0,68	0,64	0,67	0,66	0,64	0,65	0,70	0,69	0,64	0,67	0,67	0,67	0,65	0,65	0,66	0,65	0,69	0,92	0,82	0,88	0,70	0,66	0,83	0,91	0,70	0,65
NC_034556.1	0,70	0,69	0,67	0,69	0,72	0,68	0,73	0,71	0,71	0,74	0,69	0,69	0,68	0,69	0,69	0,67	0,66	0,72	0,66	0,68	0,71	0,82	0,66	0,69	0,66	0,81	0,70
NC_034560.1	0,70	0,71	0,67	0,70	0,71	0,66	0,71	0,70	0,74	0,66	0,70	0,70	0,70	0,69	0,69	0,69	0,68	0,70	0,71	0,67	0,69	0,80	0,70	0,71	0,69	0,79	0,68
NC_034564.1	0,73	0,74	0,70	0,74	0,76	0,72	0,71	0,78	0,69	0,77	0,74	0,74	0,74	0,74	0,74	0,73	0,72	0,74	0,64	0,61	0,64	0,65	0,65	0,66	0,64	0,65	0,86
NC_038529.1	0,62	0,66	0,63	0,66	0,64	0,62	0,64	0,66	0,67	0,62	0,65	0,66	0,65	0,64	0,64	0,64	0,64	0,64	0,80	0,73	0,79	0,68	0,65	0,77	0,78	0,67	0,63
NC_043068.1	0,69	0,69	0,67	0,68	0,70	0,68	0,72	0,71	0,71	0,66	0,68	0,68	0,68	0,67	0,67	0,67	0,66	0,69	0,72	0,66	0,69	0,77	0,69	0,68	0,71	0,77	0,66
NC_043175.1	0,63	0,66	0,62	0,65	0,66	0,69	0,68	0,69	0,70	0,67	0,64	0,65	0,65	0,65	0,65	0,65	0,64	0,66	0,66	0,66	0,66	0,77	0,69	0,66	0,66	0,77	0,66
NC_043407.1	0,66	0,65	0,62	0,65	0,66	0,66	0,62	0,66	0,66	0,65	0,65	0,65	0,64	0,65	0,65	0,64	0,64	0,68	0,75	0,84	0,80	0,66	0,66	0,87	0,75	0,67	0,67
NC_055147.1	0,64	0,66	0,64	0,66	0,70	0,66	0,69	0,68	0,67	0,64	0,65	0,66	0,64	0,65	0,65	0,64	0,64	0,66	0,70	0,64	0,66	0,75	0,65	0,66	0,67	0,76	0,66
NC_055632.1	0,72	0,74	0,71	0,73	0,78	0,70	0,75	0,75	0,77	0,73	0,73	0,72	0,73	0,73	0,73	0,72	0,71	0,74	0,66	0,66	0,64	0,68	0,60	0,66	0,63	0,67	0,69
NC_055636.1	0,68	0,69	0,65	0,68	0,67	0,66	0,67	0,70	0,69	0,66	0,68	0,68	0,68	0,66	0,66	0,67	0,67	0,69	0,87	0,81	0,89	0,71	0,67	0,84	0,86	0,71	0,66
NC_078485.1	0,66	0,69	0,66	0,69	0,68	0,64	0,68	0,73	0,65	0,70	0,68	0,69	0,68	0,68	0,68	0,68	0,67	0,68	0,63	0,62	0,62	0,64	0,60	0,61	0,62	0,64	0,69

	DQ989237.1	EF397003.1	EF646763.1	EU788002.1	GU140096.1	FJ858378.1	GQ244526.1	HM015220.1	JQ026206.1	JQ083393.1	JN831945.1	JX853574.1	JX879770.1	JX028271.1	KF705679.1	KF537001.1	KM102249.1	KR072623.1	KM361055.1	KM361056.1	KM361061.1	KT885041.1	KT885044.1	KT885047.1	KT885050.1	KT934965.1	KU215675.1
Mamanguape_virus_EM_725	0,68	0,66	0,66	0,66	0,66	0,67	0,67	0,62	0,72	0,66	0,64	0,70	0,70	0,66	0,63	0,64	0,75	0,82	0,66	0,66	0,66	0,72	0,64	0,66	0,64	0,66	0,66
Mamanguape_virus_EM_711	0,68	0,66	0,69	0,66	0,67	0,69	0,68	0,64	0,71	0,67	0,67	0,70	0,70	0,67	0,65	0,65	0,76	0,79	0,69	0,69	0,69	0,71	0,65	0,67	0,67	0,67	0,67
Mamanguape_virus_EM_708	0,64	0,62	0,65	0,62	0,64	0,65	0,64	0,60	0,67	0,64	0,64	0,66	0,66	0,64	0,63	0,61	0,72	0,75	0,66	0,66	0,66	0,67	0,61	0,64	0,64	0,64	0,64
OR684449.1	0,68	0,66	0,68	0,66	0,67	0,68	0,67	0,64	0,71	0,67	0,66	0,70	0,70	0,66	0,64	0,64	0,76	0,79	0,69	0,69	0,69	0,71	0,64	0,67	0,66	0,67	0,67
MG663536.1	0,66	0,66	0,69	0,66	0,69	0,67	0,65	0,67	0,70	0,68	0,66	0,70	0,70	0,66	0,64	0,64	0,83	0,73	0,68	0,68	0,68	0,70	0,64	0,68	0,66	0,68	0,69
JN037851.1	0,61	0,68	0,65	0,66	0,64	0,70	0,69	0,62	0,64	0,63	0,64	0,65	0,65	0,65	0,64	0,63	0,81	0,71	0,64	0,64	0,64	0,63	0,63	0,63	0,65	0,63	0,64
KT316176.1	0,63	0,64	0,66	0,62	0,67	0,66	0,66	0,63	0,66	0,67	0,62	0,67	0,67	0,64	0,66	0,61	0,86	0,67	0,68	0,68	0,67	0,65	0,61	0,67	0,63	0,67	0,67
JQ287716.2	0,69	0,68	0,69	0,65	0,70	0,70	0,69	0,70	0,73	0,69	0,67	0,72	0,72	0,70	0,63	0,66	0,78	0,81	0,73	0,73	0,73	0,73	0,66	0,69	0,68	0,69	0,70

OP122967.1	0,65	0,69	0,70	0,68	0,67	0,69	0,70	0,63	0,71	0,68	0,66	0,72	0,72	0,67	0,66	0,67	0,77	0,74	0,65	0,65	0,65	0,71	0,67	0,68	0,66	0,68	0,67
JX465369.1	0,66	0,66	0,64	0,64	0,66	0,66	0,68	0,65	0,68	0,66	0,63	0,69	0,69	0,63	0,63	0,61	0,80	0,79	0,70	0,70	0,71	0,68	0,61	0,66	0,62	0,66	0,66
Mamanguape_virus_EM_709	0,68	0,65	0,68	0,65	0,67	0,68	0,67	0,64	0,70	0,67	0,66	0,70	0,70	0,66	0,64	0,64	0,76	0,79	0,68	0,68	0,68	0,70	0,64	0,67	0,66	0,67	0,67
Mamanguape_virus_EM_707	0,68	0,66	0,68	0,66	0,67	0,68	0,67	0,64	0,71	0,67	0,66	0,70	0,70	0,66	0,64	0,64	0,75	0,79	0,69	0,69	0,69	0,71	0,64	0,67	0,66	0,67	0,67
Hantavirus_HantaV1_KX442773_1	0,67	0,64	0,68	0,64	0,66	0,68	0,67	0,63	0,70	0,66	0,66	0,69	0,69	0,66	0,66	0,64	0,75	0,78	0,68	0,68	0,68	0,70	0,64	0,66	0,66	0,66	0,66
Hantavirus_HantaV1_KX442793_1	0,66	0,65	0,66	0,64	0,65	0,66	0,65	0,64	0,70	0,65	0,64	0,70	0,70	0,64	0,65	0,62	0,76	0,80	0,68	0,68	0,68	0,70	0,62	0,65	0,64	0,65	0,65
Hantavirus_HantaV1_KX442794_1	0,66	0,65	0,66	0,64	0,65	0,66	0,65	0,64	0,70	0,65	0,64	0,70	0,70	0,64	0,65	0,62	0,76	0,80	0,68	0,68	0,68	0,70	0,62	0,65	0,64	0,65	0,65
Hantavirus_HantaV2_KX442772_1	0,68	0,64	0,67	0,64	0,67	0,67	0,66	0,62	0,69	0,67	0,65	0,69	0,69	0,65	0,65	0,64	0,74	0,79	0,68	0,68	0,68	0,69	0,64	0,67	0,65	0,67	0,67
Hantavirus_HantaV2_KX442771_1	0,67	0,64	0,66	0,64	0,67	0,66	0,66	0,62	0,68	0,67	0,64	0,68	0,68	0,64	0,65	0,63	0,74	0,78	0,67	0,67	0,67	0,68	0,63	0,67	0,64	0,67	0,67
Hantavirus_HantaV3_KX442770_1	0,65	0,70	0,67	0,67	0,68	0,70	0,70	0,64	0,70	0,69	0,64	0,72	0,72	0,67	0,69	0,65	0,73	0,74	0,68	0,68	0,68	0,70	0,65	0,69	0,64	0,69	0,68
EF405801.1	0,66	0,78	0,87	0,77	0,68	0,82	0,80	0,77	0,68	0,67	0,99	0,67	0,67	0,95	0,66	0,79	0,69	0,62	0,63	0,63	0,62	0,67	0,79	0,67	1,00	0,67	0,68
NC_005217.1	0,66	0,86	0,78	0,87	0,66	0,83	0,83	0,71	0,67	0,67	0,78	0,71	0,71	0,79	0,64	1,00	0,65	0,62	0,62	0,62	0,61	0,68	1,00	0,67	0,79	0,67	0,66
NC_005226.1	0,66	0,80	0,89	0,81	0,67	0,81	0,78	0,75	0,70	0,67	0,85	0,71	0,71	0,87	0,65	0,80	0,68	0,65	0,62	0,62	0,61	0,68	0,80	0,67	0,86	0,67	0,67
NC_006435.1	0,92	0,66	0,70	0,64	0,99	0,69	0,70	0,64	0,81	1,00	0,67	0,82	0,82	0,67	0,76	0,67	0,68	0,63	0,64	0,64	0,63	0,81	0,67	0,99	0,67	0,99	0,99
NC_055170.1	0,62	0,66	0,64	0,62	0,66	0,66	0,65	0,66	0,64	0,66	0,64	0,64	0,64	0,63	0,64	0,62	0,61	0,62	0,60	0,60	0,60	0,63	0,62	0,66	0,63	0,66	0,66
AF291704.5	0,66	0,89	0,79	0,88	0,67	0,87	0,87	0,73	0,66	0,68	0,80	0,68	0,68	0,79	0,66	0,86	0,66	0,63	0,61	0,61	0,60	0,67	0,86	0,68	0,81	0,68	0,67
AY526217.1	0,65	0,77	0,86	0,77	0,67	0,80	0,78	0,74	0,67	0,66	0,97	0,66	0,66	0,93	0,66	0,77	0,67	0,63	0,62	0,62	0,63	0,66	0,77	0,66	0,96	0,66	0,67
DQ371906.1	0,93	0,66	0,71	0,63	0,96	0,70	0,69	0,65	0,82	0,96	0,68	0,82	0,82	0,67	0,75	0,67	0,69	0,64	0,64	0,64	0,63	0,81	0,67	0,96	0,68	0,96	0,96
DQ825770.1	0,66	0,69	0,64	0,65	0,68	0,69	0,68	0,65	0,68	0,67	0,65	0,67	0,67	0,66	0,61	0,63	0,74	0,76	0,69	0,69	0,69	0,66	0,63	0,67	0,64	0,67	0,68
DQ989237.1	1,00	0,65	0,68	0,61	0,93	0,69	0,68	0,61	0,81	0,92	0,66	0,80	0,80	0,65	0,73	0,66	0,67	0,66	0,62	0,62	0,61	0,81	0,66	0,92	0,66	0,92	0,93
EF397003.1	0,65	1,00	0,80	0,87	0,66	0,87	0,87	0,75	0,67	0,66	0,77	0,71	0,71	0,79	0,65	0,86	0,68	0,66	0,62	0,62	0,61	0,68	0,86	0,67	0,78	0,67	0,66
EF646763.1	0,68	0,80	1,00	0,79	0,70	0,83	0,81	0,79	0,71	0,70	0,87	0,71	0,71	0,88	0,68	0,78	0,70	0,66	0,66	0,66	0,66	0,70	0,78	0,70	0,87	0,70	0,70
EU788002.1	0,61	0,87	0,79	1,00	0,63	0,86	0,84	0,69	0,65	0,64	0,78	0,67	0,67	0,78	0,62	0,87	0,66	0,63	0,61	0,61	0,61	0,65	0,87	0,64	0,77	0,64	0,63
GU140096.1	0,93	0,66	0,70	0,63	1,00	0,70	0,71	0,64	0,82	0,99	0,68	0,81	0,81	0,68	0,76	0,66	0,69	0,64	0,64	0,64	0,63	0,82	0,66	0,98	0,68	0,98	1,00
FJ858378.1	0,69	0,87	0,83	0,86	0,70	1,00	0,96	0,74	0,69	0,69	0,81	0,68	0,68	0,82	0,68	0,83	0,71	0,67	0,65	0,65	0,64	0,69	0,83	0,69	0,82	0,69	0,70
GQ244526.1	0,68	0,87	0,81	0,84	0,71	0,96	1,00	0,74	0,67	0,70	0,79	0,66	0,66	0,80	0,70	0,83	0,69	0,66	0,65	0,65	0,64	0,67	0,83	0,70	0,80	0,70	0,71
HM015220.1	0,61	0,75	0,79	0,69	0,64	0,74	0,74	1,00	0,71	0,64	0,76	0,69	0,69	0,77	0,63	0,71	0,67	0,65	0,62	0,62	0,61	0,69	0,71	0,64	0,77	0,64	0,64
JQ026206.1	0,81	0,67	0,71	0,65	0,82	0,69	0,67	0,71	1,00	0,81	0,68	0,87	0,87	0,69	0,75	0,67	0,71	0,70	0,62	0,62	0,61	0,98	0,67	0,81	0,68	0,81	0,82
JQ083393.1	0,92	0,66	0,70	0,64	0,99	0,69	0,70	0,64	0,81	1,00	0,67	0,82	0,82	0,67	0,76	0,67	0,68	0,63	0,64	0,64	0,63	0,81	0,67	0,99	0,67	0,99	0,99
JN831945.1	0,66	0,77	0,87	0,78	0,68	0,81	0,79	0,76	0,68	0,67	1,00	0,67	0,67	0,94	0,66	0,78	0,68	0,63	0,62	0,62	0,63	0,67	0,78	0,67	0,99	0,67	0,68
JX853574.1	0,80	0,71	0,71	0,67	0,81	0,68	0,66	0,69	0,87	0,82	0,67	1,00	1,00	0,67	0,73	0,71	0,70	0,66	0,62	0,62	0,63	0,86	0,71	0,82	0,67	0,82	0,81
JX879770.1	0,80	0,71	0,71	0,67	0,81	0,68	0,66	0,69	0,87	0,82	0,67	1,00	1,00	0,67	0,73	0,71	0,70	0,66	0,62	0,62	0,63	0,86	0,71	0,82	0,67	0,82	0,81
JX028271.1	0,65	0,79	0,88	0,78	0,68	0,82	0,80	0,77	0,69	0,67	0,94	0,67	0,67	1,00	0,66	0,79	0,68	0,65	0,66	0,66	0,65	0,69	0,79	0,66	0,95	0,66	0,68
KF705679.1	0,73	0,65	0,68	0,62	0,76	0,68	0,70	0,63	0,75	0,76	0,66	0,73	0,73	0,66	1,00	0,64	0,65	0,63	0,62	0,62	0,61	0,75	0,64	0,76	0,66	0,76	0,76
KF537001.1	0,66	0,86	0,78	0,87	0,66	0,83	0,83	0,71	0,67	0,67	0,78	0,71	0,71	0,79	0,64	1,00	0,65	0,62	0,62	0,62	0,61	0,68	1,00	0,67	0,79	0,67	0,66
KM102249.1	0,67	0,68	0,70	0,66	0,69	0,71	0,69	0,67	0,71	0,68	0,68	0,70	0,70	0,68	0,65	0,65	1,00	0,72	0,67	0,67	0,65	0,70	0,65	0,69	0,69	0,69	0,69
KR072623.1	0,66	0,66	0,66	0,63	0,64	0,67	0,66	0,65	0,70	0,63	0,63	0,66	0,66	0,65	0,63	0,62	0,72	1,00	0,70	0,70	0,70	0,69	0,62	0,63	0,62	0,63	0,64
KM361055.1	0,62	0,62	0,66	0,61	0,64	0,65	0,65	0,62	0,62	0,64	0,62	0,62	0,62	0,66	0,62	0,62	0,67	0,70	1,00	1,00	0,98	0,62	0,62	0,64	0,63	0,64	0,64
KM361056.1	0,62	0,62	0,66	0,61	0,64	0,65	0,65	0,62	0,62	0,64	0,62	0,62	0,62	0,66	0,62	0,62	0,67	0,70	1,00	1,00	0,98	0,62	0,62	0,64	0,63	0,64	0,64
KM361061.1	0,61	0,61	0,66	0,61	0,63	0,64	0,64	0,61	0,61	0,63	0,63	0,63	0,63	0,65	0,61	0,61	0,65	0,70	0,98	0,98	1,00	0,61	0,61	0,63	0,62	0,63	0,63
KT885041.1	0,81	0,68	0,70	0,65	0,82	0,69	0,67	0,69	0,98	0,81	0,67	0,86	0,86	0,69	0,75	0,68	0,70	0,69	0,62	0,62	0,61	1,00	0,68	0,80	0,67	0,80	0,82
KT885044.1	0,66	0,86	0,78	0,87	0,66	0,83	0,83	0,71	0,67	0,67	0,78	0,71	0,71	0,79	0,64	1,00	0,65	0,62	0,62	0,62	0,61	0,68	1,00	0,67	0,79	0,67	0,66
KT885047.1	0,92	0,67	0,70	0,64	0,98	0,69	0,70	0,64	0,81	0,99	0,67	0,82	0,82	0,66	0,76	0,67	0,69	0,63	0,64	0,64	0,63	0,80	0,67	1,00	0,67	1,00	0,98
KT885050.1	0,66	0,78	0,87	0,77	0,68	0,82	0,80	0,77	0,68	0,67	0,99	0,67	0,67	0,95	0,66	0,79	0,69	0,62	0,63	0,63	0,62	0,67	0,79	0,67	1,00	0,67	0,68

KT934965.1	0,92	0,67	0,70	0,64	0,98	0,69	0,70	0,64	0,81	0,99	0,67	0,82	0,82	0,66	0,76	0,67	0,69	0,63	0,64	0,64	0,63	0,80	0,67	1,00	0,67	1,00	0,98
KU215675.1	0,93	0,66	0,70	0,63	1,00	0,70	0,71	0,64	0,82	0,99	0,68	0,81	0,81	0,68	0,76	0,66	0,69	0,64	0,64	0,64	0,63	0,82	0,66	0,98	0,68	0,98	1,00
KX845680.1	0,67	0,70	0,67	0,67	0,68	0,72	0,72	0,66	0,69	0,68	0,65	0,66	0,66	0,67	0,66	0,66	0,74	0,79	0,74	0,74	0,72	0,68	0,66	0,68	0,66	0,68	0,68
KY594712.1	0,92	0,66	0,70	0,64	0,99	0,69	0,70	0,64	0,81	1,00	0,67	0,82	0,82	0,67	0,76	0,67	0,68	0,63	0,64	0,64	0,63	0,81	0,67	0,99	0,67	0,99	0,99
KY662269.1	0,63	0,67	0,68	0,67	0,65	0,71	0,70	0,65	0,68	0,64	0,68	0,70	0,70	0,68	0,64	0,69	0,81	0,67	0,65	0,65	0,65	0,67	0,69	0,64	0,69	0,64	0,65
KY662275.1	0,62	0,66	0,69	0,66	0,65	0,70	0,68	0,66	0,67	0,65	0,68	0,69	0,69	0,68	0,64	0,67	0,81	0,67	0,67	0,67	0,67	0,67	0,67	0,64	0,69	0,64	0,65
MG717393.1	0,68	0,87	0,79	0,85	0,69	0,85	0,84	0,72	0,70	0,70	0,79	0,71	0,71	0,79	0,66	0,95	0,66	0,64	0,63	0,63	0,62	0,71	0,95	0,70	0,80	0,70	0,69
KY978757.1	0,92	0,66	0,68	0,63	0,97	0,68	0,69	0,63	0,80	0,98	0,66	0,81	0,81	0,66	0,75	0,66	0,66	0,61	0,63	0,63	0,62	0,82	0,66	0,97	0,66	0,97	0,97
MN183135.1	0,66	0,79	0,88	0,79	0,69	0,81	0,79	0,76	0,69	0,69	0,87	0,70	0,70	0,88	0,66	0,80	0,68	0,63	0,64	0,64	0,63	0,69	0,80	0,68	0,87	0,68	0,69
MN850093.1	0,66	0,88	0,80	0,89	0,67	0,87	0,87	0,73	0,66	0,68	0,80	0,68	0,68	0,79	0,66	0,86	0,66	0,63	0,61	0,61	0,60	0,67	0,86	0,68	0,81	0,68	0,67
MN850096.1	0,66	0,88	0,80	0,88	0,67	0,87	0,87	0,73	0,66	0,68	0,81	0,68	0,68	0,79	0,66	0,86	0,66	0,63	0,61	0,61	0,60	0,67	0,86	0,68	0,82	0,68	0,67
MN639737.1	0,64	0,77	0,85	0,80	0,67	0,79	0,78	0,74	0,66	0,66	0,98	0,66	0,66	0,92	0,66	0,80	0,66	0,61	0,62	0,62	0,63	0,66	0,80	0,66	0,98	0,66	0,67
MN639740.1	0,64	0,77	0,85	0,80	0,67	0,79	0,78	0,74	0,66	0,66	0,98	0,66	0,66	0,92	0,66	0,80	0,66	0,61	0,62	0,62	0,63	0,66	0,80	0,66	0,98	0,66	0,67
MT024592.1	0,66	0,77	0,87	0,78	0,68	0,81	0,79	0,76	0,68	0,67	1,00	0,67	0,67	0,94	0,66	0,78	0,68	0,63	0,62	0,62	0,63	0,67	0,78	0,67	0,99	0,67	0,68
MT514291.1	0,64	0,77	0,85	0,79	0,67	0,80	0,79	0,75	0,66	0,66	0,98	0,66	0,66	0,93	0,66	0,81	0,67	0,61	0,63	0,63	0,62	0,66	0,81	0,66	0,98	0,66	0,67
MT514292.1	0,65	0,76	0,86	0,80	0,67	0,79	0,78	0,72	0,67	0,66	0,95	0,66	0,66	0,91	0,66	0,79	0,67	0,62	0,62	0,62	0,63	0,66	0,79	0,66	0,94	0,66	0,67
MT514294.1	0,66	0,77	0,87	0,78	0,68	0,81	0,79	0,76	0,68	0,67	1,00	0,67	0,67	0,94	0,66	0,78	0,68	0,63	0,62	0,62	0,63	0,67	0,78	0,67	0,99	0,67	0,68
MT514295.1	0,66	0,80	0,89	0,81	0,67	0,81	0,78	0,75	0,70	0,67	0,85	0,71	0,71	0,87	0,65	0,80	0,68	0,65	0,62	0,62	0,61	0,68	0,80	0,67	0,86	0,67	0,67
MT514296.1	0,66	0,79	0,88	0,79	0,69	0,81	0,79	0,76	0,69	0,69	0,87	0,70	0,70	0,88	0,66	0,80	0,68	0,63	0,64	0,64	0,63	0,69	0,80	0,68	0,87	0,68	0,69
MT514297.1	0,66	0,81	0,90	0,82	0,68	0,82	0,79	0,77	0,68	0,69	0,85	0,70	0,70	0,88	0,65	0,81	0,70	0,64	0,64	0,64	0,63	0,68	0,81	0,68	0,86	0,68	0,68
MT514298.1	0,66	0,80	0,89	0,81	0,67	0,81	0,78	0,75	0,70	0,67	0,85	0,71	0,71	0,87	0,65	0,80	0,68	0,65	0,62	0,62	0,61	0,68	0,80	0,67	0,86	0,67	0,67
MN258157.1	0,66	0,88	0,80	0,89	0,67	0,87	0,87	0,73	0,66	0,68	0,80	0,68	0,68	0,79	0,66	0,86	0,66	0,63	0,61	0,61	0,60	0,67	0,86	0,68	0,81	0,68	0,67
MN832779.1	0,66	0,77	0,87	0,78	0,68	0,81	0,79	0,75	0,68	0,67	0,98	0,67	0,67	0,94	0,66	0,77	0,68	0,64	0,63	0,63	0,64	0,67	0,77	0,67	0,97	0,67	0,68
MN832782.1	0,66	0,77	0,87	0,78	0,68	0,81	0,79	0,76	0,68	0,67	1,00	0,67	0,67	0,94	0,66	0,78	0,68	0,63	0,62	0,62	0,63	0,67	0,78	0,67	0,99	0,67	0,68
MT648514.1	0,61	0,61	0,62	0,61	0,61	0,63	0,62	0,58	0,61	0,61	0,59	0,62	0,62	0,61	0,63	0,60	0,65	0,71	0,87	0,87	0,86	0,61	0,60	0,61	0,60	0,61	0,61
OM030302.1	0,67	0,71	0,69	0,66	0,68	0,71	0,71	0,68	0,71	0,68	0,67	0,71	0,71	0,70	0,66	0,67	0,74	0,74	0,67	0,67	0,66	0,71	0,67	0,68	0,68	0,68	0,68
OM030305.1	0,98	0,66	0,69	0,62	0,95	0,70	0,69	0,63	0,82	0,94	0,67	0,82	0,82	0,66	0,75	0,66	0,69	0,66	0,64	0,64	0,63	0,82	0,66	0,94	0,67	0,94	0,95
OM030311.1	0,75	0,68	0,69	0,66	0,78	0,68	0,67	0,63	0,78	0,79	0,68	0,75	0,75	0,66	0,81	0,67	0,65	0,64	0,66	0,66	0,65	0,77	0,67	0,79	0,68	0,79	0,78
OK422869.1	0,80	0,67	0,69	0,64	0,81	0,68	0,66	0,69	0,97	0,80	0,66	0,86	0,86	0,68	0,76	0,67	0,69	0,68	0,61	0,61	0,61	0,98	0,67	0,79	0,66	0,79	0,81
MZ504241.1	0,81	0,71	0,71	0,66	0,82	0,67	0,66	0,69	0,87	0,82	0,66	0,98	0,98	0,66	0,72	0,71	0,71	0,68	0,63	0,63	0,64	0,86	0,71	0,83	0,66	0,83	0,82
OQ092243.1	0,63	0,86	0,77	0,87	0,65	0,83	0,84	0,71	0,65	0,66	0,79	0,67	0,67	0,77	0,65	0,86	0,64	0,61	0,60	0,60	0,61	0,66	0,86	0,66	0,78	0,66	0,65
OR148904.1	0,67	0,85	0,78	0,86	0,70	0,84	0,85	0,71	0,69	0,69	0,80	0,68	0,68	0,83	0,69	0,89	0,66	0,65	0,63	0,63	0,62	0,71	0,89	0,68	0,81	0,68	0,70
OR365538.1	0,53	0,81	0,65	0,70	0,54	0,71	0,71	0,58	0,55	0,55	0,62	0,58	0,58	0,63	0,51	0,70	0,55	0,51	0,50	0,50	0,49	0,56	0,70	0,55	0,63	0,55	0,54
NC_005235.1	0,81	0,68	0,70	0,65	0,82	0,69	0,67	0,70	0,98	0,81	0,67	0,87	0,87	0,69	0,75	0,68	0,70	0,69	0,62	0,62	0,61	0,98	0,68	0,80	0,67	0,80	0,82
NC_034401.1	0,65	0,66	0,68	0,61	0,69	0,68	0,67	0,67	0,68	0,69	0,68	0,68	0,68	0,69	0,62	0,64	0,78	0,73	0,72	0,72	0,70	0,69	0,64	0,68	0,69	0,68	0,69
NC_034399.1	0,73	0,66	0,69	0,65	0,78	0,68	0,68	0,64	0,74	0,77	0,70	0,75	0,75	0,68	0,77	0,66	0,67	0,65	0,62	0,62	0,63	0,73	0,66	0,77	0,70	0,77	0,78
NC_034402.1	0,71	0,71	0,67	0,66	0,70	0,66	0,67	0,65	0,71	0,70	0,66	0,74	0,74	0,66	0,71	0,68	0,67	0,66	0,62	0,62	0,63	0,71	0,68	0,70	0,66	0,70	0,70
NC_034403.1	0,71	0,82	0,81	0,83	0,71	0,84	0,82	0,73	0,71	0,71	0,83	0,71	0,71	0,81	0,71	0,85	0,66	0,64	0,62	0,62	0,62	0,72	0,85	0,71	0,82	0,71	0,71
NC_034407.1	0,71	0,69	0,71	0,68	0,73	0,72	0,72	0,66	0,72	0,72	0,72	0,69	0,69	0,72	0,73	0,67	0,71	0,63	0,69	0,69	0,68	0,71	0,67	0,72	0,72	0,72	0,73
NC_034467.1	0,71	0,81	0,84	0,77	0,73	0,82	0,82	0,76	0,71	0,73	0,80	0,72	0,72	0,83	0,71	0,79	0,66	0,66	0,66	0,66	0,66	0,73	0,79	0,72	0,81	0,72	0,73
NC_034485.1	0,79	0,69	0,71	0,66	0,81	0,69	0,69	0,63	0,79	0,81	0,69	0,79	0,79	0,69	0,88	0,69	0,66	0,67	0,66	0,66	0,65	0,80	0,69	0,81	0,69	0,81	0,81
NC_034515.1	0,66	0,86	0,81	0,88	0,66	0,88	0,87	0,73	0,68	0,66	0,80	0,68	0,68	0,82	0,66	0,88	0,66	0,65	0,63	0,63	0,63	0,68	0,88	0,66	0,81	0,66	0,66
NC_034517.1	0,81	0,71	0,71	0,69	0,83	0,71	0,70	0,69	0,86	0,84	0,68	0,87	0,87	0,69	0,77	0,70	0,69	0,70	0,64	0,64	0,63	0,84	0,70	0,84	0,68	0,84	0,83
NC_034519.1	0,67	0,82	0,91	0,80	0,69	0,84	0,82	0,79	0,71	0,70	0,92	0,71	0,71	0,93	0,68	0,82	0,68	0,66	0,66	0,66	0,65	0,69	0,82	0,70	0,92	0,70	0,69

NC_034556.1	0,79	0,71	0,71	0,66	0,82	0,68	0,68	0,68	0,89	0,82	0,66	0,85	0,85	0,66	0,77	0,68	0,72	0,69	0,65	0,65	0,64	0,87	0,68	0,82	0,66	0,82	0,82
NC_034560.1	0,77	0,68	0,72	0,67	0,79	0,71	0,70	0,66	0,79	0,80	0,71	0,77	0,77	0,69	0,85	0,67	0,71	0,68	0,67	0,67	0,66	0,78	0,67	0,80	0,71	0,80	0,79
NC_034564.1	0,62	0,68	0,65	0,63	0,66	0,66	0,66	0,66	0,68	0,65	0,65	0,67	0,67	0,65	0,60	0,61	0,74	0,75	0,68	0,68	0,68	0,66	0,61	0,66	0,64	0,66	0,66
NC_038529.1	0,65	0,75	0,84	0,76	0,68	0,78	0,77	0,72	0,67	0,68	0,81	0,68	0,68	0,81	0,65	0,73	0,66	0,61	0,62	0,62	0,63	0,67	0,73	0,67	0,80	0,67	0,68
NC_043068.1	0,76	0,68	0,71	0,66	0,77	0,72	0,71	0,66	0,80	0,77	0,72	0,75	0,75	0,71	0,83	0,66	0,72	0,69	0,66	0,66	0,65	0,79	0,66	0,77	0,72	0,77	0,77
NC_043175.1	0,72	0,67	0,69	0,66	0,77	0,70	0,69	0,63	0,75	0,77	0,66	0,72	0,72	0,65	0,77	0,66	0,70	0,65	0,66	0,66	0,66	0,74	0,66	0,77	0,66	0,77	0,77
NC_043407.1	0,65	0,86	0,75	0,86	0,66	0,80	0,80	0,70	0,68	0,66	0,76	0,70	0,70	0,76	0,67	0,84	0,64	0,66	0,62	0,62	0,62	0,69	0,84	0,66	0,75	0,66	0,66
NC_055147.1	0,72	0,67	0,69	0,62	0,75	0,66	0,66	0,67	0,80	0,75	0,70	0,75	0,75	0,68	0,77	0,64	0,69	0,66	0,64	0,64	0,63	0,79	0,64	0,75	0,70	0,75	0,75
NC_055632.1	0,66	0,66	0,66	0,63	0,68	0,68	0,69	0,68	0,68	0,68	0,65	0,67	0,67	0,65	0,67	0,66	0,76	0,70	0,70	0,70	0,69	0,68	0,66	0,68	0,66	0,68	0,68
NC_055636.1	0,67	0,83	0,91	0,81	0,71	0,87	0,84	0,79	0,70	0,71	0,87	0,71	0,71	0,89	0,68	0,81	0,71	0,66	0,65	0,65	0,64	0,69	0,81	0,71	0,87	0,71	0,71
NC_078485.1	0,62	0,62	0,66	0,61	0,64	0,65	0,65	0,62	0,62	0,64	0,62	0,62	0,62	0,66	0,62	0,62	0,67	0,70	1,00	1,00	0,98	0,62	0,62	0,64	0,63	0,64	0,64

	KX845680.1	KY594712.1	KY662269.1	KY662275.1	MG717393.1	KY978757.1	MN183135.1	MN850093.1	MN850096.1	MN639737.1	MN639740.1	MT024592.1	MT514291.1	MT514292.1	MT514294.1	MT514295.1	MT514296.1	MT514297.1	MT514298.1	MN258157.1	MN832779.1	MN832782.1	MT648514.1	OM030302.1	OM030305.1	OM030311.1	OK422869.1
Mamanguape_virus_EM_725	0,78	0,66	0,67	0,70	0,64	0,67	0,66	0,67	0,67	0,63	0,63	0,64	0,63	0,64	0,64	0,66	0,66	0,67	0,66	0,67	0,64	0,64	0,69	0,78	0,69	0,64	0,71
Mamanguape_virus_EM_711	0,78	0,67	0,71	0,74	0,66	0,67	0,66	0,66	0,66	0,66	0,66	0,67	0,66	0,67	0,67	0,66	0,66	0,67	0,66	0,66	0,67	0,67	0,70	0,78	0,70	0,66	0,70
Mamanguape_virus_EM_708	0,73	0,64	0,67	0,69	0,62	0,64	0,63	0,63	0,63	0,63	0,63	0,64	0,63	0,64	0,64	0,62	0,63	0,64	0,62	0,63	0,64	0,64	0,67	0,73	0,66	0,64	0,68
OR684449.1	0,79	0,67	0,70	0,73	0,65	0,67	0,67	0,66	0,66	0,66	0,66	0,66	0,66	0,66	0,66	0,66	0,67	0,68	0,66	0,66	0,66	0,66	0,69	0,77	0,70	0,66	0,70
MG663536.1	0,75	0,68	0,77	0,78	0,64	0,67	0,66	0,66	0,66	0,68	0,68	0,66	0,68	0,67	0,66	0,65	0,66	0,67	0,65	0,66	0,65	0,66	0,65	0,73	0,68	0,68	0,69
JN037851.1	0,76	0,63	0,79	0,75	0,64	0,61	0,64	0,67	0,67	0,63	0,63	0,64	0,64	0,63	0,64	0,66	0,64	0,66	0,66	0,67	0,64	0,64	0,69	0,73	0,62	0,64	0,64
KT316176.1	0,72	0,67	0,78	0,79	0,62	0,65	0,66	0,65	0,65	0,62	0,62	0,62	0,62	0,62	0,62	0,65	0,66	0,66	0,65	0,65	0,62	0,62	0,66	0,71	0,65	0,65	0,66
JQ287716.2	0,79	0,69	0,76	0,77	0,67	0,67	0,68	0,70	0,70	0,66	0,66	0,67	0,66	0,65	0,67	0,68	0,68	0,69	0,68	0,70	0,66	0,67	0,69	0,79	0,71	0,66	0,72
OP122967.1	0,76	0,68	0,74	0,77	0,70	0,67	0,68	0,68	0,68	0,66	0,66	0,66	0,66	0,66	0,66	0,67	0,68	0,70	0,67	0,68	0,66	0,66	0,65	0,72	0,66	0,69	0,70
JX465369.1	0,80	0,66	0,70	0,71	0,64	0,66	0,62	0,66	0,66	0,62	0,62	0,63	0,61	0,62	0,63	0,61	0,62	0,63	0,61	0,66	0,64	0,63	0,68	0,76	0,67	0,64	0,67
Mamanguape_virus_EM_709	0,78	0,67	0,70	0,73	0,64	0,67	0,67	0,66	0,66	0,65	0,65	0,66	0,65	0,66	0,66	0,66	0,67	0,68	0,66	0,66	0,66	0,66	0,69	0,77	0,70	0,65	0,70
Mamanguape_virus_EM_707	0,79	0,67	0,69	0,72	0,65	0,67	0,67	0,66	0,66	0,66	0,66	0,66	0,66	0,66	0,66	0,66	0,67	0,68	0,66	0,66	0,66	0,66	0,69	0,77	0,70	0,66	0,70
Hantavirus_HantaV1_KX442773_1	0,77	0,66	0,70	0,73	0,64	0,66	0,65	0,65	0,65	0,65	0,65	0,66	0,65	0,66	0,66	0,64	0,65	0,66	0,64	0,65	0,66	0,66	0,69	0,77	0,69	0,65	0,69
Hantavirus_HantaV1_KX442793_1	0,79	0,65	0,70	0,73	0,64	0,65	0,65	0,64	0,64	0,64	0,64	0,64	0,64	0,64	0,64	0,64	0,65	0,66	0,64	0,64	0,64	0,64	0,69	0,77	0,68	0,65	0,70
Hantavirus_HantaV1_KX442794_1	0,79	0,65	0,70	0,73	0,64	0,65	0,65	0,64	0,64	0,64	0,64	0,64	0,64	0,64	0,64	0,64	0,65	0,66	0,64	0,64	0,64	0,64	0,69	0,77	0,68	0,65	0,70
Hantavirus_HantaV2_KX442772_1	0,78	0,67	0,70	0,73	0,64	0,65	0,66	0,65	0,65	0,64	0,64	0,65	0,64	0,65	0,65	0,65	0,66	0,67	0,65	0,65	0,65	0,65	0,68	0,75	0,70	0,64	0,68
Hantavirus_HantaV2_KX442771_1	0,77	0,67	0,69	0,72	0,64	0,65	0,65	0,64	0,64	0,64	0,64	0,64	0,64	0,64	0,64	0,64	0,65	0,66	0,64	0,64	0,64	0,64	0,66	0,74	0,69	0,64	0,67
Hantavirus_HantaV3_KX442770_1	0,76	0,69	0,69	0,68	0,67	0,68	0,64	0,69	0,69	0,64	0,64	0,64	0,64	0,66	0,64	0,64	0,64	0,65	0,64	0,69	0,64	0,64	0,69	0,77	0,67	0,66	0,69
EF405801.1	0,66	0,67	0,69	0,69	0,80	0,66	0,87	0,81	0,82	0,98	0,98	0,99	0,98	0,94	0,99	0,86	0,87	0,86	0,86	0,81	0,97	0,99	0,60	0,68	0,67	0,68	0,66
NC_005217.1	0,66	0,67	0,69	0,67	0,95	0,66	0,80	0,86	0,86	0,80	0,80	0,78	0,81	0,79	0,78	0,80	0,80	0,81	0,80	0,86	0,77	0,78	0,60	0,67	0,66	0,67	0,67
NC_005226.1	0,66	0,67	0,68	0,67	0,78	0,66	0,97	0,82	0,82	0,83	0,83	0,85	0,84	0,84	0,85	1,00	0,98	0,96	1,00	0,82	0,85	0,85	0,61	0,68	0,66	0,66	0,67
NC_006435.1	0,68	1,00	0,64	0,65	0,70	0,98	0,69	0,68	0,68	0,66	0,66	0,67	0,66	0,66	0,67	0,67	0,69	0,69	0,67	0,68	0,67	0,67	0,61	0,68	0,94	0,79	0,80
NC_055170.1	0,63	0,66	0,62	0,62	0,65	0,65	0,66	0,66	0,66	0,63	0,63	0,64	0,62	0,63	0,64	0,66	0,66	0,66	0,66	0,66	0,64	0,64	0,59	0,65	0,63	0,65	0,62
AF291704.5	0,68	0,68	0,68	0,68	0,87	0,68	0,82	0,99	0,98	0,79	0,79	0,80	0,80	0,78	0,80	0,81	0,82	0,82	0,81	0,99	0,78	0,80	0,61	0,67	0,66	0,67	0,66
AY526217.1	0,65	0,66	0,66	0,66	0,77	0,65	0,86	0,77	0,78	0,95	0,95	0,97	0,94	0,93	0,97	0,84	0,86	0,84	0,84	0,77	0,99	0,97	0,58	0,66	0,66	0,68	0,66
DQ371906.1	0,68	0,96	0,66	0,66	0,70	0,93	0,70	0,66	0,66	0,66	0,66	0,68	0,66	0,66	0,68	0,69	0,70	0,68	0,69	0,66	0,68	0,68	0,61	0,69	0,96	0,78	0,80
DQ825770.1	0,82	0,67	0,74	0,73	0,66	0,65	0,62	0,66	0,66	0,64	0,64	0,65	0,63	0,64	0,65	0,63	0,62	0,63	0,63	0,66	0,65	0,65	0,69	0,78	0,66	0,62	0,66
DQ989237.1	0,67	0,92	0,63	0,62	0,68	0,92	0,66	0,66	0,66	0,64	0,64	0,66	0,64	0,65	0,66	0,66	0,66	0,66	0,66	0,66	0,66	0,66	0,61	0,67	0,98	0,75	0,80

EF397003.1	0,70	0,66	0,67	0,66	0,87	0,66	0,79	0,88	0,88	0,77	0,77	0,77	0,77	0,76	0,77	0,80	0,79	0,81	0,80	0,88	0,77	0,77	0,61	0,71	0,66	0,68	0,67
EF646763.1	0,67	0,70	0,68	0,69	0,79	0,68	0,88	0,80	0,80	0,85	0,85	0,87	0,85	0,86	0,87	0,89	0,88	0,90	0,89	0,80	0,87	0,87	0,62	0,69	0,69	0,69	0,69
EU788002.1	0,67	0,64	0,67	0,66	0,85	0,63	0,79	0,89	0,88	0,80	0,80	0,78	0,79	0,80	0,78	0,81	0,79	0,82	0,81	0,89	0,78	0,78	0,61	0,66	0,62	0,66	0,64
GU140096.1	0,68	0,99	0,65	0,65	0,69	0,97	0,69	0,67	0,67	0,67	0,67	0,68	0,67	0,67	0,68	0,67	0,69	0,68	0,67	0,67	0,68	0,68	0,61	0,68	0,95	0,78	0,81
FJ858378.1	0,72	0,69	0,71	0,70	0,85	0,68	0,81	0,87	0,87	0,79	0,79	0,81	0,80	0,79	0,81	0,81	0,81	0,82	0,81	0,87	0,81	0,81	0,63	0,71	0,70	0,68	0,68
GQ244526.1	0,72	0,70	0,70	0,68	0,84	0,69	0,79	0,87	0,87	0,78	0,78	0,79	0,79	0,78	0,79	0,78	0,79	0,79	0,78	0,87	0,79	0,79	0,62	0,71	0,69	0,67	0,66
HM015220.1	0,66	0,64	0,65	0,66	0,72	0,63	0,76	0,73	0,73	0,74	0,74	0,76	0,75	0,72	0,76	0,75	0,76	0,77	0,75	0,73	0,75	0,76	0,58	0,68	0,63	0,63	0,69
JQ026206.1	0,69	0,81	0,68	0,67	0,70	0,80	0,69	0,66	0,66	0,66	0,66	0,68	0,66	0,67	0,68	0,70	0,69	0,68	0,70	0,66	0,68	0,68	0,61	0,71	0,82	0,78	0,97
JQ083393.1	0,68	1,00	0,64	0,65	0,70	0,98	0,69	0,68	0,68	0,66	0,66	0,67	0,66	0,66	0,67	0,67	0,69	0,69	0,67	0,68	0,67	0,67	0,61	0,68	0,94	0,79	0,80
JN831945.1	0,65	0,67	0,68	0,68	0,79	0,66	0,87	0,80	0,81	0,98	0,98	1,00	0,98	0,95	1,00	0,85	0,87	0,85	0,85	0,80	0,98	1,00	0,59	0,67	0,67	0,68	0,66
JX853574.1	0,66	0,82	0,70	0,69	0,71	0,81	0,70	0,68	0,68	0,66	0,66	0,67	0,66	0,66	0,67	0,71	0,70	0,70	0,71	0,68	0,67	0,67	0,62	0,71	0,82	0,75	0,86
JX879770.1	0,66	0,82	0,70	0,69	0,71	0,81	0,70	0,68	0,68	0,66	0,66	0,67	0,66	0,66	0,67	0,71	0,70	0,70	0,71	0,68	0,67	0,67	0,62	0,71	0,82	0,75	0,86
JX028271.1	0,67	0,67	0,68	0,68	0,79	0,66	0,88	0,79	0,79	0,92	0,92	0,94	0,93	0,91	0,94	0,87	0,88	0,88	0,87	0,79	0,94	0,94	0,61	0,70	0,66	0,66	0,68
KF705679.1	0,66	0,76	0,64	0,64	0,66	0,75	0,66	0,66	0,66	0,66	0,66	0,66	0,66	0,66	0,66	0,65	0,66	0,65	0,65	0,66	0,66	0,66	0,63	0,66	0,75	0,81	0,76
KF537001.1	0,66	0,67	0,69	0,67	0,95	0,66	0,80	0,86	0,86	0,80	0,80	0,78	0,81	0,79	0,78	0,80	0,80	0,81	0,80	0,86	0,77	0,78	0,60	0,67	0,66	0,67	0,67
KM102249.1	0,74	0,68	0,81	0,81	0,66	0,66	0,68	0,66	0,66	0,66	0,66	0,68	0,67	0,67	0,68	0,68	0,68	0,70	0,68	0,66	0,68	0,68	0,65	0,74	0,69	0,65	0,69
KR072623.1	0,79	0,63	0,67	0,67	0,64	0,61	0,63	0,63	0,63	0,61	0,61	0,63	0,61	0,62	0,63	0,65	0,63	0,64	0,65	0,63	0,64	0,63	0,71	0,74	0,66	0,64	0,68
KM361055.1	0,74	0,64	0,65	0,67	0,63	0,63	0,64	0,61	0,61	0,62	0,62	0,62	0,63	0,62	0,62	0,62	0,64	0,64	0,62	0,61	0,63	0,62	0,87	0,67	0,64	0,66	0,61
KM361056.1	0,74	0,64	0,65	0,67	0,63	0,63	0,64	0,61	0,61	0,62	0,62	0,62	0,63	0,62	0,62	0,62	0,64	0,64	0,62	0,61	0,63	0,62	0,87	0,67	0,64	0,66	0,61
KM361061.1	0,72	0,63	0,65	0,67	0,62	0,62	0,63	0,60	0,60	0,63	0,63	0,63	0,62	0,63	0,63	0,61	0,63	0,63	0,61	0,60	0,64	0,63	0,86	0,66	0,63	0,65	0,61
KT885041.1	0,68	0,81	0,67	0,67	0,71	0,82	0,69	0,67	0,67	0,66	0,66	0,67	0,66	0,66	0,67	0,68	0,69	0,68	0,68	0,67	0,67	0,67	0,61	0,71	0,82	0,77	0,98
KT885044.1	0,66	0,67	0,69	0,67	0,95	0,66	0,80	0,86	0,86	0,80	0,80	0,78	0,81	0,79	0,78	0,80	0,80	0,81	0,80	0,86	0,77	0,78	0,60	0,67	0,66	0,67	0,67
KT885047.1	0,68	0,99	0,64	0,64	0,70	0,97	0,68	0,68	0,68	0,66	0,66	0,67	0,66	0,66	0,67	0,67	0,68	0,68	0,67	0,68	0,67	0,67	0,61	0,68	0,94	0,79	0,79
KT885050.1	0,66	0,67	0,69	0,69	0,80	0,66	0,87	0,81	0,82	0,98	0,98	0,99	0,98	0,94	0,99	0,86	0,87	0,86	0,86	0,81	0,97	0,99	0,60	0,68	0,67	0,68	0,66
KT934965.1	0,68	0,99	0,64	0,64	0,70	0,97	0,68	0,68	0,68	0,66	0,66	0,67	0,66	0,66	0,67	0,67	0,68	0,68	0,67	0,68	0,67	0,67	0,61	0,68	0,94	0,79	0,79
KU215675.1	0,68	0,99	0,65	0,65	0,69	0,97	0,69	0,67	0,67	0,67	0,67	0,68	0,67	0,67	0,68	0,67	0,69	0,68	0,67	0,67	0,68	0,68	0,61	0,68	0,95	0,78	0,81
KX845680.1	1,00	0,68	0,69	0,71	0,68	0,66	0,66	0,68	0,68	0,66	0,66	0,65	0,66	0,66	0,65	0,66	0,66	0,67	0,66	0,68	0,66	0,65	0,73	0,81	0,68	0,67	0,67
KY594712.1	0,68	1,00	0,64	0,65	0,70	0,98	0,69	0,68	0,68	0,66	0,66	0,67	0,66	0,66	0,67	0,67	0,69	0,69	0,67	0,68	0,67	0,67	0,61	0,68	0,94	0,79	0,80
KY662269.1	0,69	0,64	1,00	0,95	0,69	0,62	0,67	0,68	0,68	0,66	0,66	0,68	0,67	0,66	0,68	0,68	0,67	0,68	0,68	0,68	0,67	0,68	0,66	0,74	0,65	0,66	0,66
KY662275.1	0,71	0,65	0,95	1,00	0,69	0,63	0,69	0,68	0,68	0,66	0,66	0,68	0,67	0,66	0,68	0,67	0,69	0,70	0,67	0,68	0,67	0,68	0,65	0,72	0,63	0,67	0,66
MG717393.1	0,68	0,70	0,69	0,69	1,00	0,69	0,80	0,87	0,87	0,79	0,79	0,79	0,80	0,78	0,79	0,78	0,80	0,81	0,78	0,87	0,78	0,79	0,61	0,69	0,69	0,69	0,70
KY978757.1	0,66	0,98	0,62	0,63	0,69	1,00	0,67	0,68	0,68	0,65	0,65	0,66	0,65	0,65	0,66	0,66	0,67	0,67	0,66	0,68	0,66	0,66	0,60	0,68	0,93	0,77	0,81
MN183135.1	0,66	0,69	0,67	0,69	0,80	0,67	1,00	0,82	0,82	0,85	0,85	0,87	0,86	0,85	0,87	0,97	0,99	0,96	0,97	0,82	0,87	0,87	0,59	0,66	0,67	0,67	0,68
MN850093.1	0,68	0,68	0,68	0,68	0,87	0,68	0,82	1,00	0,99	0,79	0,79	0,80	0,80	0,78	0,80	0,82	0,82	0,83	0,82	1,00	0,78	0,80	0,61	0,67	0,66	0,67	0,66
MN850096.1	0,68	0,68	0,68	0,68	0,87	0,68	0,82	0,99	1,00	0,80	0,80	0,81	0,81	0,79	0,81	0,82	0,82	0,83	0,82	0,99	0,79	0,81	0,61	0,67	0,66	0,67	0,66
MN639737.1	0,66	0,66	0,66	0,66	0,79	0,65	0,85	0,79	0,80	1,00	1,00	0,98	0,99	0,97	0,98	0,83	0,85	0,83	0,83	0,79	0,96	0,98	0,59	0,66	0,66	0,68	0,65
MN639740.1	0,66	0,66	0,66	0,66	0,79	0,65	0,85	0,79	0,80	1,00	1,00	0,98	0,99	0,97	0,98	0,83	0,85	0,83	0,83	0,79	0,96	0,98	0,59	0,66	0,66	0,68	0,65
MT024592.1	0,65	0,67	0,68	0,68	0,79	0,66	0,87	0,80	0,81	0,98	0,98	1,00	0,98	0,95	1,00	0,85	0,87	0,85	0,85	0,80	0,98	1,00	0,59	0,67	0,67	0,68	0,66
MT514291.1	0,66	0,66	0,67	0,67	0,80	0,65	0,86	0,80	0,81	0,99	0,99	0,98	1,00	0,96	0,98	0,84	0,86	0,84	0,84	0,80	0,95	0,98	0,60	0,66	0,66	0,68	0,65
MT514292.1	0,66	0,66	0,66	0,66	0,78	0,65	0,85	0,78	0,79	0,97	0,97	0,95	0,96	1,00	0,95	0,84	0,85	0,83	0,84	0,78	0,94	0,95	0,59	0,66	0,66	0,68	0,65
MT514294.1	0,65	0,67	0,68	0,68	0,79	0,66	0,87	0,80	0,81	0,98	0,98	1,00	0,98	0,95	1,00	0,85	0,87	0,85	0,85	0,80	0,98	1,00	0,59	0,67	0,67	0,68	0,66
MT514295.1	0,66	0,67	0,68	0,67	0,78	0,66	0,97	0,82	0,82	0,83	0,83	0,85	0,84	0,84	0,85	1,00	0,98	0,96	1,00	0,82	0,85	0,85	0,61	0,68	0,66	0,66	0,67
MT514296.1	0,66	0,69	0,67	0,69	0,80	0,67	0,99	0,82	0,82	0,85	0,85	0,87	0,86	0,85	0,87	0,98	1,00	0,97	0,98	0,82	0,87	0,87	0,59	0,66	0,67	0,67	0,68
MT514297.1	0,67	0,69	0,68	0,70	0,81	0,67	0,96	0,83	0,83	0,83	0,83	0,85	0,84	0,83	0,85	0,96	0,97	1,00	0,96	0,83	0,85	0,85	0,61	0,67	0,66	0,66	0,67

MT514298.1	0,66	0,67	0,68	0,67	0,78	0,66	0,97	0,82	0,82	0,83	0,83	0,85	0,84	0,84	0,85	1,00	0,98	0,96	1,00	0,82	0,85	0,85	0,61	0,68	0,66	0,66	0,67
MN258157.1	0,68	0,68	0,68	0,68	0,87	0,68	0,82	1,00	0,99	0,79	0,79	0,80	0,80	0,78	0,80	0,82	0,82	0,83	0,82	1,00	0,78	0,80	0,61	0,67	0,66	0,67	0,66
MN832779.1	0,66	0,67	0,67	0,67	0,78	0,66	0,87	0,78	0,79	0,96	0,96	0,98	0,95	0,94	0,98	0,85	0,87	0,85	0,85	0,78	1,00	0,98	0,59	0,67	0,67	0,69	0,66
MN832782.1	0,65	0,67	0,68	0,68	0,79	0,66	0,87	0,80	0,81	0,98	0,98	1,00	0,98	0,95	1,00	0,85	0,87	0,85	0,85	0,80	0,98	1,00	0,59	0,67	0,67	0,68	0,66
MT648514.1	0,73	0,61	0,66	0,65	0,61	0,60	0,59	0,61	0,61	0,59	0,59	0,59	0,60	0,59	0,59	0,61	0,59	0,61	0,61	0,61	0,59	0,59	1,00	0,69	0,62	0,63	0,61
OM030302.1	0,81	0,68	0,74	0,72	0,69	0,68	0,66	0,67	0,67	0,66	0,66	0,67	0,66	0,66	0,67	0,68	0,66	0,67	0,68	0,67	0,67	0,67	0,69	1,00	0,68	0,65	0,70
OM030305.1	0,68	0,94	0,65	0,63	0,69	0,93	0,67	0,66	0,66	0,66	0,66	0,67	0,66	0,66	0,67	0,66	0,67	0,66	0,66	0,66	0,67	0,67	0,62	0,68	1,00	0,77	0,82
OM030311.1	0,67	0,79	0,66	0,67	0,69	0,77	0,67	0,67	0,67	0,68	0,68	0,68	0,68	0,68	0,68	0,66	0,67	0,66	0,66	0,67	0,69	0,68	0,63	0,65	0,77	1,00	0,78
OK422869.1	0,67	0,80	0,66	0,66	0,70	0,81	0,68	0,66	0,66	0,65	0,65	0,66	0,65	0,65	0,66	0,67	0,68	0,67	0,67	0,66	0,66	0,66	0,61	0,70	0,82	0,78	1,00
MZ504241.1	0,68	0,82	0,69	0,70	0,71	0,81	0,69	0,67	0,67	0,65	0,65	0,66	0,65	0,66	0,66	0,70	0,69	0,69	0,70	0,67	0,66	0,66	0,61	0,70	0,82	0,75	0,86
OQ092243.1	0,65	0,66	0,66	0,66	0,85	0,66	0,80	0,93	0,93	0,78	0,78	0,79	0,77	0,77	0,79	0,78	0,80	0,81	0,78	0,93	0,77	0,79	0,59	0,66	0,64	0,66	0,65
OR148904.1	0,69	0,69	0,69	0,67	0,87	0,69	0,82	0,87	0,86	0,81	0,81	0,80	0,82	0,81	0,80	0,82	0,82	0,82	0,82	0,87	0,79	0,80	0,62	0,71	0,68	0,67	0,70
OR365538.1	0,56	0,55	0,54	0,53	0,71	0,55	0,64	0,71	0,71	0,61	0,61	0,62	0,62	0,61	0,62	0,64	0,64	0,65	0,64	0,71	0,62	0,62	0,48	0,57	0,54	0,54	0,55
NC_005235.1	0,68	0,81	0,67	0,67	0,71	0,82	0,69	0,67	0,67	0,66	0,66	0,67	0,66	0,66	0,67	0,68	0,69	0,68	0,68	0,67	0,67	0,67	0,61	0,71	0,82	0,77	0,99
NC_034401.1	0,77	0,69	0,75	0,77	0,65	0,68	0,68	0,66	0,66	0,67	0,67	0,68	0,68	0,66	0,68	0,66	0,68	0,69	0,66	0,66	0,67	0,68	0,67	0,75	0,66	0,66	0,68
NC_034399.1	0,68	0,77	0,66	0,68	0,66	0,76	0,69	0,69	0,69	0,70	0,70	0,70	0,70	0,69	0,70	0,68	0,69	0,68	0,68	0,69	0,69	0,70	0,61	0,66	0,75	0,79	0,74
NC_034402.1	0,66	0,70	0,64	0,65	0,69	0,70	0,67	0,69	0,69	0,66	0,66	0,66	0,65	0,66	0,66	0,68	0,67	0,67	0,68	0,69	0,66	0,66	0,61	0,67	0,71	0,69	0,71
NC_034403.1	0,66	0,71	0,69	0,69	0,87	0,71	0,82	0,86	0,86	0,82	0,82	0,83	0,81	0,81	0,83	0,82	0,82	0,82	0,82	0,86	0,82	0,83	0,61	0,69	0,72	0,70	0,71
NC_034407.1	0,71	0,72	0,70	0,69	0,68	0,71	0,71	0,68	0,68	0,72	0,72	0,72	0,72	0,72	0,72	0,70	0,71	0,70	0,70	0,68	0,73	0,72	0,62	0,69	0,71	0,78	0,71
NC_034467.1	0,70	0,73	0,67	0,69	0,82	0,73	0,82	0,81	0,81	0,79	0,79	0,80	0,80	0,79	0,80	0,80	0,82	0,82	0,80	0,81	0,82	0,80	0,62	0,71	0,72	0,71	0,72
NC_034485.1	0,67	0,81	0,67	0,66	0,70	0,81	0,69	0,70	0,70	0,68	0,68	0,69	0,68	0,68	0,69	0,69	0,69	0,67	0,69	0,70	0,69	0,69	0,68	0,70	0,81	0,87	0,81
NC_034515.1	0,70	0,66	0,68	0,67	0,87	0,65	0,82	0,87	0,87	0,80	0,80	0,80	0,81	0,80	0,80	0,81	0,82	0,82	0,81	0,87	0,80	0,80	0,60	0,69	0,66	0,66	0,67
NC_034517.1	0,71	0,84	0,68	0,70	0,73	0,82	0,69	0,69	0,69	0,68	0,68	0,68	0,68	0,68	0,68	0,69	0,69	0,69	0,69	0,69	0,68	0,68	0,62	0,71	0,82	0,79	0,84
NC_034519.1	0,69	0,70	0,67	0,68	0,83	0,68	0,89	0,83	0,84	0,90	0,90	0,92	0,91	0,90	0,92	0,88	0,89	0,90	0,88	0,83	0,92	0,92	0,61	0,71	0,68	0,70	0,68
NC_034556.1	0,71	0,82	0,67	0,66	0,69	0,82	0,69	0,69	0,69	0,66	0,66	0,66	0,66	0,66	0,66	0,71	0,69	0,69	0,71	0,69	0,66	0,66	0,64	0,71	0,80	0,80	0,88
NC_034560.1	0,71	0,80	0,71	0,72	0,70	0,78	0,70	0,71	0,71	0,71	0,71	0,71	0,71	0,70	0,71	0,69	0,70	0,70	0,69	0,71	0,70	0,71	0,66	0,70	0,78	0,83	0,77
NC_034564.1	0,78	0,65	0,68	0,69	0,63	0,63	0,64	0,66	0,66	0,64	0,64	0,65	0,63	0,64	0,65	0,64	0,65	0,66	0,64	0,66	0,65	0,65	0,65	0,74	0,63	0,62	0,66
NC_038529.1	0,64	0,68	0,67	0,69	0,74	0,66	0,81	0,77	0,77	0,80	0,80	0,81	0,79	0,79	0,81	0,79	0,81	0,81	0,79	0,77	0,79	0,81	0,59	0,65	0,66	0,66	0,66
NC_043068.1	0,68	0,77	0,72	0,72	0,67	0,75	0,69	0,68	0,68	0,71	0,71	0,72	0,71	0,70	0,72	0,69	0,69	0,68	0,69	0,68	0,71	0,72	0,65	0,69	0,77	0,84	0,80
NC_043175.1	0,67	0,77	0,69	0,71	0,70	0,75	0,66	0,67	0,67	0,66	0,66	0,66	0,66	0,66	0,66	0,66	0,66	0,66	0,66	0,67	0,67	0,66	0,61	0,69	0,74	0,79	0,73
NC_043407.1	0,70	0,66	0,69	0,67	0,83	0,66	0,79	0,87	0,86	0,77	0,77	0,76	0,76	0,78	0,76	0,80	0,80	0,79	0,80	0,87	0,76	0,76	0,61	0,68	0,66	0,71	0,68
NC_055147.1	0,69	0,75	0,65	0,66	0,67	0,73	0,68	0,66	0,66	0,69	0,69	0,70	0,69	0,67	0,70	0,66	0,68	0,66	0,66	0,66	0,68	0,70	0,61	0,68	0,74	0,78	0,79
NC_055632.1	0,74	0,68	0,73	0,75	0,67	0,67	0,65	0,66	0,66	0,65	0,65	0,65	0,66	0,64	0,65	0,64	0,65	0,66	0,64	0,66	0,64	0,65	0,69	0,72	0,67	0,71	0,69
NC_055636.1	0,69	0,71	0,67	0,68	0,83	0,69	0,89	0,85	0,85	0,85	0,85	0,87	0,86	0,85	0,87	0,89	0,89	0,89	0,89	0,85	0,87	0,87	0,63	0,71	0,69	0,68	0,68
NC_078485.1	0,74	0,64	0,65	0,67	0,63	0,63	0,64	0,61	0,61	0,62	0,62	0,62	0,63	0,62	0,62	0,62	0,64	0,64	0,62	0,61	0,63	0,62	0,87	0,67	0,64	0,66	0,61

	MZ504241.1	OQ092243.1	OR148904.1	OR365538.1	NC_005235.1	NC_034401.1	NC_034399.1	NC_034402.1	NC_034403.1	NC_034407.1	NC_034467.1	NC_034485.1	NC_034515.1	NC_034517.1	NC_034519.1	NC_034556.1	NC_034560.1	NC_034564.1	NC_038529.1	NC_043068.1	NC_043175.1	NC_043407.1	NC_055147.1	NC_055632.1	NC_055636.1	NC_078485.1
Mamanguape_virus_EM_725	0,71	0,64	0,69	0,50	0,72	0,76	0,68	0,66	0,66	0,64	0,68	0,68	0,67	0,71	0,67	0,70	0,70	0,73	0,62	0,69	0,63	0,66	0,64	0,72	0,68	0,66
Mamanguape_virus_EM_711	0,71	0,64	0,68	0,52	0,71	0,78	0,70	0,66	0,66	0,68	0,70	0,68	0,67	0,71	0,68	0,69	0,71	0,74	0,66	0,69	0,66	0,65	0,66	0,74	0,69	0,69
Mamanguape_virus_EM_708	0,67	0,60	0,64	0,52	0,67	0,72	0,68	0,62	0,63	0,64	0,66	0,66	0,64	0,68	0,64	0,67	0,67	0,70	0,63	0,67	0,62	0,62	0,64	0,71	0,65	0,66
OR684449.1	0,71	0,64	0,67	0,52	0,71	0,78	0,71	0,66	0,66	0,67	0,69	0,67	0,66	0,71	0,67	0,69	0,70	0,74	0,66	0,68	0,65	0,65	0,66	0,73	0,68	0,69

MG663536.1	0,70	0,65	0,67	0,52	0,70	0,79	0,68	0,67	0,65	0,70	0,65	0,69	0,65	0,70	0,66	0,72	0,71	0,76	0,64	0,70	0,66	0,66	0,70	0,78	0,67	0,68
JN037851.1	0,65	0,64	0,66	0,54	0,63	0,71	0,66	0,66	0,61	0,67	0,63	0,66	0,65	0,66	0,64	0,68	0,66	0,72	0,62	0,68	0,69	0,66	0,66	0,70	0,66	0,64
KT316176.1	0,68	0,62	0,63	0,51	0,65	0,75	0,69	0,68	0,61	0,70	0,65	0,68	0,62	0,66	0,65	0,73	0,71	0,71	0,64	0,72	0,68	0,62	0,69	0,75	0,67	0,68
JQ287716.2	0,74	0,68	0,68	0,54	0,73	0,78	0,71	0,69	0,66	0,67	0,69	0,67	0,68	0,72	0,70	0,71	0,70	0,78	0,66	0,71	0,69	0,66	0,68	0,75	0,70	0,73
OP122967.1	0,72	0,66	0,68	0,58	0,71	0,78	0,74	0,66	0,64	0,70	0,70	0,69	0,68	0,72	0,69	0,71	0,74	0,69	0,67	0,71	0,70	0,66	0,67	0,77	0,69	0,65
JX465369.1	0,70	0,66	0,62	0,55	0,68	0,75	0,66	0,70	0,61	0,67	0,64	0,65	0,62	0,68	0,64	0,74	0,66	0,77	0,62	0,66	0,67	0,65	0,64	0,73	0,66	0,70
Mamanguape_virus_EM_709	0,70	0,64	0,67	0,52	0,70	0,78	0,70	0,65	0,65	0,67	0,69	0,67	0,66	0,70	0,67	0,69	0,70	0,74	0,65	0,68	0,64	0,65	0,65	0,73	0,68	0,68
Mamanguape_virus_EM_707	0,71	0,64	0,67	0,52	0,71	0,78	0,71	0,66	0,66	0,67	0,69	0,67	0,66	0,71	0,67	0,69	0,70	0,74	0,66	0,68	0,65	0,65	0,66	0,72	0,68	0,69
Hantavirus_HantaV1_KX442773_1	0,70	0,63	0,67	0,50	0,70	0,77	0,69	0,64	0,65	0,67	0,69	0,67	0,66	0,70	0,67	0,68	0,70	0,74	0,65	0,68	0,65	0,64	0,64	0,73	0,68	0,68
Hantavirus_HantaV1_KX442793_1	0,70	0,62	0,65	0,51	0,70	0,78	0,69	0,65	0,64	0,67	0,67	0,66	0,64	0,70	0,65	0,69	0,69	0,74	0,64	0,67	0,65	0,65	0,65	0,73	0,66	0,68
Hantavirus_HantaV1_KX442794_1	0,70	0,62	0,65	0,51	0,70	0,78	0,69	0,65	0,64	0,67	0,67	0,66	0,64	0,70	0,65	0,69	0,69	0,74	0,64	0,67	0,65	0,65	0,65	0,73	0,66	0,68
Hantavirus_HantaV2_KX442772_1	0,70	0,63	0,66	0,50	0,69	0,78	0,70	0,64	0,64	0,66	0,68	0,66	0,65	0,70	0,66	0,67	0,69	0,73	0,64	0,67	0,65	0,64	0,64	0,72	0,67	0,68
Hantavirus_HantaV2_KX442771_1	0,70	0,62	0,65	0,50	0,68	0,78	0,69	0,64	0,64	0,65	0,67	0,65	0,64	0,70	0,65	0,66	0,68	0,72	0,64	0,66	0,64	0,64	0,64	0,71	0,67	0,67
Hantavirus_HantaV3_KX442770_1	0,71	0,66	0,69	0,56	0,70	0,77	0,68	0,67	0,67	0,68	0,69	0,69	0,67	0,71	0,69	0,72	0,70	0,74	0,64	0,69	0,66	0,68	0,66	0,74	0,69	0,68
EF405801.1	0,66	0,78	0,81	0,63	0,67	0,69	0,70	0,66	0,82	0,72	0,81	0,69	0,81	0,68	0,92	0,66	0,71	0,64	0,80	0,72	0,66	0,75	0,70	0,66	0,87	0,63
NC_005217.1	0,71	0,86	0,89	0,70	0,68	0,64	0,66	0,68	0,85	0,67	0,79	0,69	0,88	0,70	0,82	0,68	0,67	0,61	0,73	0,66	0,66	0,84	0,64	0,66	0,81	0,62
NC_005226.1	0,70	0,78	0,82	0,64	0,68	0,66	0,68	0,68	0,82	0,70	0,80	0,69	0,81	0,69	0,88	0,71	0,69	0,64	0,79	0,69	0,66	0,80	0,66	0,64	0,89	0,62
NC_006435.1	0,82	0,66	0,69	0,55	0,81	0,69	0,77	0,70	0,71	0,72	0,73	0,81	0,66	0,84	0,70	0,82	0,80	0,65	0,68	0,77	0,77	0,66	0,75	0,68	0,71	0,64
NC_055170.1	0,64	0,63	0,64	0,53	0,63	0,63	0,68	0,69	0,66	0,67	0,66	0,66	0,63	0,66	0,66	0,66	0,70	0,65	0,65	0,69	0,69	0,66	0,65	0,60	0,67	0,60
AF291704.5	0,67	0,93	0,87	0,72	0,67	0,66	0,69	0,69	0,85	0,68	0,81	0,70	0,87	0,69	0,83	0,69	0,71	0,66	0,77	0,68	0,66	0,87	0,66	0,66	0,84	0,61
AY526217.1	0,66	0,77	0,78	0,61	0,66	0,66	0,68	0,66	0,82	0,72	0,81	0,68	0,79	0,67	0,91	0,66	0,69	0,64	0,78	0,71	0,66	0,75	0,67	0,63	0,86	0,62
DQ371906.1	0,83	0,64	0,67	0,54	0,81	0,68	0,77	0,70	0,74	0,72	0,72	0,80	0,66	0,82	0,70	0,81	0,79	0,65	0,67	0,77	0,77	0,67	0,76	0,67	0,71	0,64
DQ825770.1	0,69	0,65	0,66	0,56	0,66	0,75	0,68	0,70	0,65	0,72	0,66	0,65	0,65	0,71	0,65	0,70	0,68	0,86	0,63	0,66	0,66	0,67	0,66	0,69	0,66	0,69
DQ989237.1	0,81	0,63	0,67	0,53	0,81	0,65	0,73	0,71	0,71	0,71	0,71	0,79	0,66	0,81	0,67	0,79	0,77	0,62	0,65	0,76	0,72	0,65	0,72	0,66	0,67	0,62
EF397003.1	0,71	0,86	0,85	0,81	0,68	0,66	0,66	0,71	0,82	0,69	0,81	0,69	0,86	0,71	0,82	0,71	0,68	0,68	0,75	0,68	0,67	0,86	0,67	0,66	0,83	0,62
EF646763.1	0,71	0,77	0,78	0,65	0,70	0,68	0,69	0,67	0,81	0,71	0,84	0,71	0,81	0,71	0,91	0,71	0,72	0,65	0,84	0,71	0,69	0,75	0,69	0,66	0,91	0,66
EU788002.1	0,66	0,87	0,86	0,70	0,65	0,61	0,65	0,66	0,83	0,68	0,77	0,66	0,88	0,69	0,80	0,66	0,67	0,63	0,76	0,66	0,66	0,86	0,62	0,63	0,81	0,61
GU140096.1	0,82	0,65	0,70	0,54	0,82	0,69	0,78	0,70	0,71	0,73	0,73	0,81	0,66	0,83	0,69	0,82	0,79	0,66	0,68	0,77	0,77	0,66	0,75	0,68	0,71	0,64
FJ858378.1	0,67	0,83	0,84	0,71	0,69	0,68	0,68	0,66	0,84	0,72	0,82	0,69	0,88	0,71	0,84	0,68	0,71	0,66	0,78	0,72	0,70	0,80	0,66	0,68	0,87	0,65
GQ244526.1	0,66	0,84	0,85	0,71	0,67	0,67	0,68	0,67	0,82	0,72	0,82	0,69	0,87	0,70	0,82	0,68	0,70	0,66	0,77	0,71	0,69	0,80	0,66	0,69	0,84	0,65
HM015220.1	0,69	0,71	0,71	0,58	0,70	0,67	0,64	0,65	0,73	0,66	0,76	0,63	0,73	0,69	0,79	0,68	0,66	0,66	0,72	0,66	0,63	0,70	0,67	0,68	0,79	0,62
JQ026206.1	0,87	0,65	0,69	0,55	0,98	0,68	0,74	0,71	0,71	0,72	0,71	0,79	0,68	0,86	0,71	0,89	0,79	0,68	0,67	0,80	0,75	0,68	0,80	0,68	0,70	0,62
JQ083393.1	0,82	0,66	0,69	0,55	0,81	0,69	0,77	0,70	0,71	0,72	0,73	0,81	0,66	0,84	0,70	0,82	0,80	0,65	0,68	0,77	0,77	0,66	0,75	0,68	0,71	0,64
JN831945.1	0,66	0,79	0,80	0,62	0,67	0,68	0,70	0,66	0,83	0,72	0,80	0,69	0,80	0,68	0,92	0,66	0,71	0,65	0,81	0,72	0,66	0,76	0,70	0,65	0,87	0,62
JX853574.1	0,98	0,67	0,68	0,58	0,87	0,68	0,75	0,74	0,71	0,69	0,72	0,79	0,68	0,87	0,71	0,85	0,77	0,67	0,68	0,75	0,72	0,70	0,75	0,67	0,71	0,62
JX879770.1	0,98	0,67	0,68	0,58	0,87	0,68	0,75	0,74	0,71	0,69	0,72	0,79	0,68	0,87	0,71	0,85	0,77	0,67	0,68	0,75	0,72	0,70	0,75	0,67	0,71	0,62
JX028271.1	0,66	0,77	0,83	0,63	0,69	0,69	0,68	0,66	0,81	0,72	0,83	0,69	0,82	0,69	0,93	0,66	0,69	0,65	0,81	0,71	0,65	0,76	0,68	0,65	0,89	0,66
KF705679.1	0,72	0,65	0,69	0,51	0,75	0,62	0,77	0,71	0,71	0,73	0,71	0,88	0,66	0,77	0,68	0,77	0,85	0,60	0,65	0,83	0,77	0,67	0,77	0,67	0,68	0,62
KF537001.1	0,71	0,86	0,89	0,70	0,68	0,64	0,66	0,68	0,85	0,67	0,79	0,69	0,88	0,70	0,82	0,68	0,67	0,61	0,73	0,66	0,66	0,84	0,64	0,66	0,81	0,62
KM102249.1	0,71	0,64	0,66	0,55	0,70	0,78	0,67	0,67	0,66	0,71	0,66	0,66	0,66	0,69	0,68	0,72	0,71	0,74	0,66	0,72	0,70	0,64	0,69	0,76	0,71	0,67
KR072623.1	0,68	0,61	0,65	0,51	0,69	0,73	0,65	0,66	0,64	0,63	0,66	0,67	0,65	0,70	0,66	0,69	0,68	0,75	0,61	0,69	0,65	0,66	0,66	0,70	0,66	0,70
KM361055.1	0,63	0,60	0,63	0,50	0,62	0,72	0,62	0,62	0,62	0,69	0,66	0,66	0,63	0,64	0,66	0,65	0,67	0,68	0,62	0,66	0,66	0,62	0,64	0,70	0,65	1,00
KM361056.1	0,63	0,60	0,63	0,50	0,62	0,72	0,62	0,62	0,62	0,69	0,66	0,66	0,63	0,64	0,66	0,65	0,67	0,68	0,62	0,66	0,66	0,62	0,64	0,70	0,65	1,00
KM361061.1	0,64	0,61	0,62	0,49	0,61	0,70	0,63	0,63	0,62	0,68	0,66	0,65	0,63	0,63	0,65	0,64	0,66	0,68	0,63	0,65	0,66	0,62	0,63	0,69	0,64	0,98

KT885041.1	0,86	0,66	0,71	0,56	0,98	0,69	0,73	0,71	0,72	0,71	0,73	0,80	0,68	0,84	0,69	0,87	0,78	0,66	0,67	0,79	0,74	0,69	0,79	0,68	0,69	0,62
KT885044.1	0,71	0,86	0,89	0,70	0,68	0,64	0,66	0,68	0,85	0,67	0,79	0,69	0,88	0,70	0,82	0,68	0,67	0,61	0,73	0,66	0,66	0,84	0,64	0,66	0,81	0,62
KT885047.1	0,83	0,66	0,68	0,55	0,80	0,68	0,77	0,70	0,71	0,72	0,72	0,81	0,66	0,84	0,70	0,82	0,80	0,66	0,67	0,77	0,77	0,66	0,75	0,68	0,71	0,64
KT885050.1	0,66	0,78	0,81	0,63	0,67	0,69	0,70	0,66	0,82	0,72	0,81	0,69	0,81	0,68	0,92	0,66	0,71	0,64	0,80	0,72	0,66	0,75	0,70	0,66	0,87	0,63
KT934965.1	0,83	0,66	0,68	0,55	0,80	0,68	0,77	0,70	0,71	0,72	0,72	0,81	0,66	0,84	0,70	0,82	0,80	0,66	0,67	0,77	0,77	0,66	0,75	0,68	0,71	0,64
KU215675.1	0,82	0,65	0,70	0,54	0,82	0,69	0,78	0,70	0,71	0,73	0,73	0,81	0,66	0,83	0,69	0,82	0,79	0,66	0,68	0,77	0,77	0,66	0,75	0,68	0,71	0,64
KX845680.1	0,68	0,65	0,69	0,56	0,68	0,77	0,68	0,66	0,66	0,71	0,70	0,67	0,70	0,71	0,69	0,71	0,71	0,78	0,64	0,68	0,67	0,70	0,69	0,74	0,69	0,74
KY594712.1	0,82	0,66	0,69	0,55	0,81	0,69	0,77	0,70	0,71	0,72	0,73	0,81	0,66	0,84	0,70	0,82	0,80	0,65	0,68	0,77	0,77	0,66	0,75	0,68	0,71	0,64
KY662269.1	0,69	0,66	0,69	0,54	0,67	0,75	0,66	0,64	0,69	0,70	0,67	0,67	0,68	0,68	0,67	0,67	0,71	0,68	0,67	0,72	0,69	0,69	0,65	0,73	0,67	0,65
KY662275.1	0,70	0,66	0,67	0,53	0,67	0,77	0,68	0,65	0,69	0,69	0,69	0,66	0,67	0,70	0,68	0,66	0,72	0,69	0,69	0,72	0,71	0,67	0,66	0,75	0,68	0,67
MG717393.1	0,71	0,85	0,87	0,71	0,71	0,65	0,66	0,69	0,87	0,68	0,82	0,70	0,87	0,73	0,83	0,69	0,70	0,63	0,74	0,67	0,70	0,83	0,67	0,67	0,83	0,63
KY978757.1	0,81	0,66	0,69	0,55	0,82	0,68	0,76	0,70	0,71	0,71	0,73	0,81	0,65	0,82	0,68	0,82	0,78	0,63	0,66	0,75	0,75	0,66	0,73	0,67	0,69	0,63
MN183135.1	0,69	0,80	0,82	0,64	0,69	0,68	0,69	0,67	0,82	0,71	0,82	0,69	0,82	0,69	0,89	0,69	0,70	0,64	0,81	0,69	0,66	0,79	0,68	0,65	0,89	0,64
MN850093.1	0,67	0,93	0,87	0,71	0,67	0,66	0,69	0,69	0,86	0,68	0,81	0,70	0,87	0,69	0,83	0,69	0,71	0,66	0,77	0,68	0,67	0,87	0,66	0,66	0,85	0,61
MN850096.1	0,67	0,93	0,86	0,71	0,67	0,66	0,69	0,69	0,86	0,68	0,81	0,70	0,87	0,69	0,84	0,69	0,71	0,66	0,77	0,68	0,67	0,86	0,66	0,66	0,85	0,61
MN639737.1	0,65	0,78	0,81	0,61	0,66	0,67	0,70	0,66	0,82	0,72	0,79	0,68	0,80	0,68	0,90	0,66	0,71	0,64	0,80	0,71	0,66	0,77	0,69	0,65	0,85	0,62
MN639740.1	0,65	0,78	0,81	0,61	0,66	0,67	0,70	0,66	0,82	0,72	0,79	0,68	0,80	0,68	0,90	0,66	0,71	0,64	0,80	0,71	0,66	0,77	0,69	0,65	0,85	0,62
MT024592.1	0,66	0,79	0,80	0,62	0,67	0,68	0,70	0,66	0,83	0,72	0,80	0,69	0,80	0,68	0,92	0,66	0,71	0,65	0,81	0,72	0,66	0,76	0,70	0,65	0,87	0,62
MT514291.1	0,65	0,77	0,82	0,62	0,66	0,68	0,70	0,65	0,81	0,72	0,80	0,68	0,81	0,68	0,91	0,66	0,71	0,63	0,79	0,71	0,66	0,76	0,69	0,66	0,86	0,63
MT514292.1	0,66	0,77	0,81	0,61	0,66	0,66	0,69	0,66	0,81	0,72	0,79	0,68	0,80	0,68	0,90	0,66	0,70	0,64	0,79	0,70	0,66	0,78	0,67	0,64	0,85	0,62
MT514294.1	0,66	0,79	0,80	0,62	0,67	0,68	0,70	0,66	0,83	0,72	0,80	0,69	0,80	0,68	0,92	0,66	0,71	0,65	0,81	0,72	0,66	0,76	0,70	0,65	0,87	0,62
MT514295.1	0,70	0,78	0,82	0,64	0,68	0,66	0,68	0,68	0,82	0,70	0,80	0,69	0,81	0,69	0,88	0,71	0,69	0,64	0,79	0,69	0,66	0,80	0,66	0,64	0,89	0,62
MT514296.1	0,69	0,80	0,82	0,64	0,69	0,68	0,69	0,67	0,82	0,71	0,82	0,69	0,82	0,69	0,89	0,69	0,70	0,65	0,81	0,69	0,66	0,80	0,68	0,65	0,89	0,64
MT514297.1	0,69	0,81	0,82	0,65	0,68	0,69	0,68	0,67	0,82	0,70	0,82	0,67	0,82	0,69	0,90	0,69	0,70	0,66	0,81	0,68	0,66	0,79	0,66	0,66	0,89	0,64
MT514298.1	0,70	0,78	0,82	0,64	0,68	0,66	0,68	0,68	0,82	0,70	0,80	0,69	0,81	0,69	0,88	0,71	0,69	0,64	0,79	0,69	0,66	0,80	0,66	0,64	0,89	0,62
MN258157.1	0,67	0,93	0,87	0,71	0,67	0,66	0,69	0,69	0,86	0,68	0,81	0,70	0,87	0,69	0,83	0,69	0,71	0,66	0,77	0,68	0,67	0,87	0,66	0,66	0,85	0,61
MN832779.1	0,66	0,77	0,79	0,62	0,67	0,67	0,69	0,66	0,82	0,73	0,82	0,69	0,80	0,68	0,92	0,66	0,70	0,65	0,79	0,71	0,67	0,76	0,68	0,64	0,87	0,63
MN832782.1	0,66	0,79	0,80	0,62	0,67	0,68	0,70	0,66	0,83	0,72	0,80	0,69	0,80	0,68	0,92	0,66	0,71	0,65	0,81	0,72	0,66	0,76	0,70	0,65	0,87	0,62
MT648514.1	0,61	0,59	0,62	0,48	0,61	0,67	0,61	0,61	0,61	0,62	0,62	0,68	0,60	0,62	0,61	0,64	0,66	0,65	0,59	0,65	0,61	0,61	0,61	0,69	0,63	0,87
OM030302.1	0,70	0,66	0,71	0,57	0,71	0,75	0,66	0,67	0,69	0,69	0,71	0,70	0,69	0,71	0,71	0,71	0,70	0,74	0,65	0,69	0,69	0,68	0,68	0,72	0,71	0,67
OM030305.1	0,82	0,64	0,68	0,54	0,82	0,66	0,75	0,71	0,72	0,71	0,72	0,81	0,66	0,82	0,68	0,80	0,78	0,63	0,66	0,77	0,74	0,66	0,74	0,67	0,69	0,64
OM030311.1	0,75	0,66	0,67	0,54	0,77	0,66	0,79	0,69	0,70	0,78	0,71	0,87	0,66	0,79	0,70	0,80	0,83	0,62	0,66	0,84	0,79	0,71	0,78	0,71	0,68	0,66
OK422869.1	0,86	0,65	0,70	0,55	0,99	0,68	0,74	0,71	0,71	0,71	0,72	0,81	0,67	0,84	0,68	0,88	0,77	0,66	0,66	0,80	0,73	0,68	0,79	0,69	0,68	0,61
MZ504241.1	1,00	0,66	0,67	0,57	0,87	0,69	0,75	0,75	0,71	0,69	0,71	0,78	0,67	0,88	0,70	0,86	0,77	0,68	0,67	0,76	0,74	0,69	0,75	0,67	0,70	0,63
OQ092243.1	0,66	1,00	0,84	0,70	0,66	0,63	0,66	0,67	0,83	0,66	0,77	0,68	0,85	0,66	0,81	0,68	0,68	0,65	0,75	0,66	0,65	0,86	0,64	0,65	0,79	0,60
OR148904.1	0,67	0,84	1,00	0,67	0,71	0,66	0,70	0,70	0,87	0,71	0,82	0,73	0,90	0,70	0,82	0,69	0,70	0,65	0,76	0,70	0,66	0,87	0,67	0,69	0,82	0,63
OR365538.1	0,57	0,70	0,67	1,00	0,56	0,54	0,53	0,57	0,66	0,58	0,65	0,55	0,69	0,57	0,65	0,57	0,55	0,55	0,61	0,55	0,54	0,68	0,55	0,52	0,66	0,50
NC_005235.1	0,87	0,66	0,71	0,56	1,00	0,69	0,73	0,71	0,72	0,71	0,73	0,80	0,68	0,85	0,69	0,87	0,78	0,67	0,67	0,79	0,74	0,69	0,78	0,68	0,69	0,62
NC_034401.1	0,69	0,63	0,66	0,54	0,69	1,00	0,70	0,66	0,66	0,71	0,68	0,65	0,65	0,67	0,69	0,71	0,69	0,73	0,65	0,70	0,67	0,64	0,70	0,80	0,71	0,72
NC_034399.1	0,75	0,66	0,70	0,53	0,73	0,70	1,00	0,75	0,71	0,77	0,71	0,80	0,68	0,76	0,69	0,77	0,81	0,66	0,69	0,82	0,78	0,65	0,78	0,69	0,71	0,62
NC_034402.1	0,75	0,67	0,70	0,57	0,71	0,66	0,75	1,00	0,71	0,76	0,71	0,75	0,65	0,71	0,67	0,76	0,77	0,69	0,70	0,75	0,73	0,70	0,74	0,64	0,68	0,62
NC_034403.1	0,71	0,83	0,87	0,66	0,72	0,66	0,71	0,71	1,00	0,71	0,81	0,74	0,86	0,73	0,82	0,69	0,74	0,66	0,79	0,73	0,71	0,83	0,70	0,66	0,85	0,62
NC_034407.1	0,69	0,66	0,71	0,58	0,71	0,71	0,77	0,76	0,71	1,00	0,74	0,74	0,71	0,73	0,71	0,71	0,77	0,69	0,71	0,76	0,74	0,70	0,77	0,68	0,71	0,69
NC_034467.1	0,71	0,77	0,82	0,65	0,73	0,68	0,71	0,71	0,81	0,74	1,00	0,74	0,82	0,74	0,86	0,71	0,73	0,66	0,87	0,71	0,70	0,76	0,71	0,66	0,85	0,66

NC_034485.1	0,78	0,68	0,73	0,55	0,80	0,65	0,80	0,75	0,74	0,74	0,74	1,00	0,68	0,82	0,71	0,81	0,87	0,63	0,66	0,87	0,77	0,72	0,77	0,72	0,71	0,66
NC_034515.1	0,67	0,85	0,90	0,69	0,68	0,65	0,68	0,65	0,86	0,71	0,82	0,68	1,00	0,71	0,85	0,65	0,68	0,65	0,77	0,68	0,66	0,84	0,66	0,67	0,85	0,63
NC_034517.1	0,88	0,66	0,70	0,57	0,85	0,67	0,76	0,71	0,73	0,73	0,74	0,82	0,71	1,00	0,71	0,83	0,82	0,69	0,70	0,80	0,77	0,70	0,76	0,69	0,71	0,64
NC_034519.1	0,70	0,81	0,82	0,65	0,69	0,69	0,69	0,67	0,82	0,71	0,86	0,71	0,85	0,71	1,00	0,71	0,72	0,66	0,82	0,71	0,67	0,78	0,69	0,67	0,92	0,66
NC_034556.1	0,86	0,68	0,69	0,57	0,87	0,71	0,77	0,76	0,69	0,71	0,71	0,81	0,65	0,83	0,71	1,00	0,81	0,68	0,67	0,80	0,77	0,69	0,78	0,70	0,70	0,65
NC_034560.1	0,77	0,68	0,70	0,55	0,78	0,69	0,81	0,77	0,74	0,77	0,73	0,87	0,68	0,82	0,72	0,81	1,00	0,66	0,71	0,92	0,82	0,70	0,81	0,70	0,72	0,67
NC_034564.1	0,68	0,65	0,65	0,55	0,67	0,73	0,66	0,69	0,66	0,69	0,66	0,63	0,65	0,69	0,66	0,68	0,66	1,00	0,64	0,65	0,64	0,67	0,66	0,69	0,67	0,68
NC_038529.1	0,67	0,75	0,76	0,61	0,67	0,65	0,69	0,70	0,79	0,71	0,87	0,66	0,77	0,70	0,82	0,67	0,71	0,64	1,00	0,69	0,66	0,71	0,68	0,63	0,82	0,62
NC_043068.1	0,76	0,66	0,70	0,55	0,79	0,70	0,82	0,75	0,73	0,76	0,71	0,87	0,68	0,80	0,71	0,80	0,92	0,65	0,69	1,00	0,82	0,70	0,81	0,71	0,71	0,66
NC_043175.1	0,74	0,65	0,66	0,54	0,74	0,67	0,78	0,73	0,71	0,74	0,70	0,77	0,66	0,77	0,67	0,77	0,82	0,64	0,66	0,82	1,00	0,69	0,78	0,66	0,70	0,66
NC_043407.1	0,69	0,86	0,87	0,68	0,69	0,64	0,65	0,70	0,83	0,70	0,76	0,72	0,84	0,70	0,78	0,69	0,70	0,67	0,71	0,70	0,69	1,00	0,66	0,64	0,79	0,62
NC_055147.1	0,75	0,64	0,67	0,55	0,78	0,70	0,78	0,74	0,70	0,77	0,71	0,77	0,66	0,76	0,69	0,78	0,81	0,66	0,68	0,81	0,78	0,66	1,00	0,70	0,70	0,64
NC_055632.1	0,67	0,65	0,69	0,52	0,68	0,80	0,69	0,64	0,66	0,68	0,66	0,72	0,67	0,69	0,67	0,70	0,70	0,69	0,63	0,71	0,66	0,64	0,70	1,00	0,67	0,70
NC_055636.1	0,70	0,79	0,82	0,66	0,69	0,71	0,71	0,68	0,85	0,71	0,85	0,71	0,85	0,71	0,92	0,70	0,72	0,67	0,82	0,71	0,70	0,79	0,70	0,67	1,00	0,65
NC_078485.1	0,63	0,60	0,63	0,50	0,62	0,72	0,62	0,62	0,62	0,69	0,66	0,66	0,63	0,64	0,66	0,65	0,67	0,68	0,62	0,66	0,66	0,62	0,64	0,70	0,65	1,00

TABLE B

Percent identity matrix based on nucleotides

	Mamanguape_virus_EM_725	Mamanguape_virus_EM_711	Mamanguape_virus_EM_708	OR684449.1	MG663536.1	JN037851.1	KT316176.1	JQ287716.2	OP122967.1	JX465369.1	Mamanguape_virus_EM_709	Mamanguape_virus_EM_707	Hantavirus_HantaV1_KX442773_1	Hantavirus_HantaV1_KX442793_1	Hantavirus_HantaV1_KX442794_1	Hantavirus_HantaV2_KX442772_1	Hantavirus_HantaV2_KX442771_1	Hantavirus_HantaV3_KX442770_1	EF405801.1	NC_005217.1	NC_005226.1	NC_006435.1	NC_055170.1	AF291704.5	AY526217.1	DQ371906.1	DQ825770.1
Mamanguape_virus_EM_725	1,00	0,99	0,96	0,99	0,75	0,69	0,70	0,83	0,80	0,82	0,99	0,99	0,99	0,98	0,98	0,97	0,97	0,82	0,64	0,64	0,66	0,66	0,58	0,67	0,63	0,67	0,73
Mamanguape_virus_EM_711	0,99	1,00	0,93	0,99	0,76	0,73	0,74	0,80	0,81	0,80	0,99	0,98	1,00	0,98	0,98	0,97	0,95	0,81	0,67	0,65	0,66	0,67	0,59	0,66	0,66	0,68	0,74
Mamanguape_virus_EM_708	0,96	0,93	1,00	0,93	0,72	0,69	0,71	0,76	0,77	0,77	0,93	0,92	0,93	0,92	0,92	0,91	0,90	0,77	0,64	0,61	0,62	0,64	0,56	0,63	0,63	0,64	0,70
OR684449.1	0,99	0,99	0,93	1,00	0,76	0,74	0,74	0,80	0,82	0,80	1,00	0,99	0,99	0,99	0,99	0,97	0,96	0,81	0,66	0,64	0,66	0,67	0,60	0,66	0,66	0,68	0,74
MG663536.1	0,75	0,76	0,72	0,76	1,00	0,74	0,78	0,78	0,75	0,78	0,76	0,75	0,75	0,76	0,76	0,74	0,74	0,74	0,66	0,64	0,65	0,68	0,60	0,66	0,64	0,68	0,76
JN037851.1	0,69	0,73	0,69	0,74	0,74	1,00	0,79	0,74	0,72	0,73	0,73	0,73	0,72	0,74	0,74	0,73	0,71	0,72	0,65	0,63	0,66	0,63	0,61	0,66	0,63	0,62	0,77
KT316176.1	0,70	0,74	0,71	0,74	0,78	0,79	1,00	0,76	0,76	0,76	0,74	0,73	0,74	0,74	0,74	0,74	0,74	0,74	0,63	0,61	0,65	0,67	0,62	0,65	0,62	0,66	0,73
JQ287716.2	0,83	0,80	0,76	0,80	0,78	0,74	0,76	1,00	0,76	0,83	0,80	0,80	0,80	0,80	0,80	0,81	0,80	0,76	0,68	0,66	0,68	0,69	0,63	0,70	0,66	0,70	0,76
OP122967.1	0,80	0,81	0,77	0,82	0,75	0,72	0,76	0,76	1,00	0,77	0,82	0,81	0,80	0,82	0,82	0,80	0,80	0,75	0,66	0,67	0,67	0,68	0,60	0,68	0,66	0,66	0,69
JX465369.1	0,82	0,80	0,77	0,80	0,78	0,73	0,76	0,83	0,77	1,00	0,79	0,80	0,79	0,80	0,80	0,77	0,77	0,75	0,62	0,61	0,61	0,66	0,61	0,66	0,63	0,66	0,75
Mamanguape_virus_EM_709	0,99	0,99	0,93	1,00	0,76	0,73	0,74	0,80	0,82	0,79	1,00	0,99	0,99	0,99	0,99	0,97	0,96	0,81	0,66	0,64	0,66	0,67	0,59	0,66	0,65	0,68	0,74
Mamanguape_virus_EM_707	0,99	0,98	0,92	0,99	0,75	0,73	0,73	0,80	0,81	0,80	0,99	1,00	0,98	0,98	0,98	0,97	0,95	0,81	0,66	0,64	0,66	0,67	0,60	0,66	0,66	0,68	0,74
Hantavirus_HantaV1_KX442773_1	0,99	1,00	0,93	0,99	0,75	0,72	0,74	0,80	0,80	0,79	0,99	0,98	1,00	0,98	0,98	0,97	0,95	0,81	0,66	0,64	0,64	0,66	0,57	0,65	0,65	0,67	0,74
Hantavirus_HantaV1_KX442793_1	0,98	0,98	0,92	0,99	0,76	0,74	0,74	0,80	0,82	0,80	0,99	0,98	0,98	1,00	1,00	0,97	0,95	0,82	0,64	0,62	0,64	0,65	0,59	0,64	0,64	0,66	0,74
Hantavirus_HantaV1_KX442794_1	0,98	0,98	0,92	0,99	0,76	0,74	0,74	0,80	0,82	0,80	0,99	0,98	0,98	1,00	1,00	0,97	0,95	0,82	0,64	0,62	0,64	0,65	0,59	0,64	0,64	0,66	0,74
Hantavirus_HantaV2_KX442772_1	0,97	0,97	0,91	0,97	0,74	0,73	0,74	0,81	0,80	0,77	0,97	0,97	0,97	0,97	0,97	1,00	0,98	0,81	0,65	0,64	0,65	0,67	0,58	0,65	0,64	0,68	0,74
Hantavirus_HantaV2_KX442771_1	0,97	0,95	0,90	0,96	0,74	0,71	0,74	0,80	0,80	0,77	0,96	0,95	0,95	0,95	0,95	0,98	1,00	0,80	0,64	0,63	0,64	0,67	0,57	0,64	0,64	0,67	0,73
Hantavirus_HantaV3_KX442770_1	0,82	0,81	0,77	0,81	0,74	0,72	0,74	0,76	0,75	0,75	0,81	0,81	0,81	0,82	0,82	0,81	0,80	1,00	0,64	0,65	0,64	0,69	0,62	0,69	0,64	0,67	0,75
EF405801.1	0,64	0,67	0,64	0,66	0,66	0,65	0,63	0,68	0,66	0,62	0,66	0,66	0,66	0,64	0,64	0,65	0,64	0,64	1,00	0,79	0,86	0,67	0,63	0,81	0,96	0,68	0,64
NC_005217.1	0,64	0,65	0,61	0,64	0,64	0,63	0,61	0,66	0,67	0,61	0,64	0,64	0,64	0,62	0,62	0,64	0,63	0,65	0,79	1,00	0,80	0,67	0,62	0,86	0,77	0,67	0,63
NC_005226.1	0,66	0,66	0,62	0,66	0,65	0,66	0,65	0,68	0,67	0,61	0,66	0,66	0,64	0,64	0,64	0,65	0,64	0,64	0,86	0,80	1,00	0,67	0,66	0,81	0,84	0,69	0,63
NC_006435.1	0,66	0,67	0,64	0,67	0,68	0,63	0,67	0,69	0,68	0,66	0,67	0,67	0,66	0,65	0,65	0,67	0,67	0,69	0,67	0,67	0,67	1,00	0,66	0,68	0,66	0,96	0,67
NC_055170.1	0,58	0,59	0,56	0,60	0,60	0,61	0,62	0,63	0,60	0,61	0,59	0,60	0,57	0,59	0,59	0,58	0,57	0,62	0,63	0,62	0,66	0,66	1,00	0,66	0,63	0,66	0,67
AF291704.5	0,67	0,66	0,63	0,66	0,66	0,66	0,65	0,70	0,68	0,66	0,66	0,66	0,65	0,64	0,64	0,65	0,64	0,69	0,81	0,86	0,81	0,68	0,66	1,00	0,77	0,66	0,66
AY526217.1	0,63	0,66	0,63	0,66	0,64	0,63	0,62	0,66	0,66	0,63	0,65	0,66	0,65	0,64	0,64	0,64	0,64	0,64	0,96	0,77	0,84	0,66	0,63	0,77	1,00	0,67	0,64
DQ371906.1	0,67	0,68	0,64	0,68	0,68	0,62	0,66	0,70	0,66	0,66	0,68	0,68	0,67	0,66	0,66	0,68	0,67	0,67	0,68	0,67	0,69	0,96	0,66	0,66	0,67	1,00	0,67
DQ825770.1	0,73	0,74	0,70	0,74	0,76	0,77	0,73	0,76	0,69	0,75	0,74	0,74	0,74	0,74	0,74	0,74	0,73	0,75	0,64	0,63	0,63	0,67	0,67	0,66	0,64	0,67	1,00
DQ989237.1	0,68	0,68	0,64	0,68	0,66	0,61	0,63	0,69	0,65	0,66	0,68	0,68	0,67	0,66	0,66	0,68	0,67	0,65	0,66	0,66	0,66	0,92	0,62	0,66	0,65	0,93	0,66
EF397003.1	0,66	0,66	0,62	0,66	0,66	0,68	0,64	0,68	0,69	0,66	0,65	0,66	0,64	0,65	0,65	0,64	0,64	0,70	0,78	0,86	0,80	0,66	0,66	0,89	0,77	0,66	0,69
EF646763.1	0,66	0,69	0,65	0,68	0,69	0,65	0,66	0,69	0,70	0,64	0,68	0,68	0,68	0,66	0,66	0,67	0,66	0,67	0,87	0,78	0,89	0,70	0,64	0,79	0,86	0,71	0,64
EU788002.1	0,66	0,66	0,62	0,66	0,66	0,66	0,62	0,65	0,68	0,64	0,65	0,66	0,64	0,64	0,64	0,64	0,64	0,67	0,77	0,87	0,81	0,64	0,62	0,88	0,77	0,63	0,65
GU140096.1	0,66	0,67	0,64	0,67	0,69	0,64	0,67	0,70	0,67	0,66	0,67	0,67	0,66	0,65	0,65	0,67	0,67	0,68	0,68	0,66	0,67	0,99	0,66	0,67	0,67	0,96	0,68
FJ858378.1	0,67	0,69	0,65	0,68	0,67	0,70	0,66	0,70	0,69	0,66	0,68	0,68	0,68	0,66	0,66	0,67	0,66	0,70	0,82	0,83	0,81	0,69	0,66	0,87	0,80	0,70	0,69

GQ244526.1	0,67	0,68	0,64	0,67	0,65	0,69	0,66	0,69	0,70	0,68	0,67	0,67	0,67	0,65	0,65	0,66	0,66	0,70	0,80	0,83	0,78	0,70	0,65	0,87	0,78	0,69	0,68
HM015220.1	0,62	0,64	0,60	0,64	0,67	0,62	0,63	0,70	0,63	0,65	0,64	0,64	0,63	0,64	0,64	0,62	0,62	0,64	0,77	0,71	0,75	0,64	0,66	0,73	0,74	0,65	0,65
JQ026206.1	0,72	0,71	0,67	0,71	0,70	0,64	0,66	0,73	0,71	0,68	0,70	0,71	0,70	0,70	0,70	0,69	0,68	0,70	0,68	0,67	0,70	0,81	0,64	0,66	0,67	0,82	0,68
JQ083393.1	0,66	0,67	0,64	0,67	0,68	0,63	0,67	0,69	0,68	0,66	0,67	0,67	0,66	0,65	0,65	0,67	0,67	0,69	0,67	0,67	0,67	1,00	0,66	0,68	0,66	0,96	0,67
JN831945.1	0,64	0,67	0,64	0,66	0,66	0,64	0,62	0,67	0,66	0,63	0,66	0,66	0,66	0,64	0,64	0,65	0,64	0,64	0,99	0,78	0,85	0,67	0,64	0,80	0,97	0,68	0,65
JX853574.1	0,70	0,70	0,66	0,70	0,70	0,65	0,67	0,72	0,72	0,69	0,70	0,70	0,69	0,70	0,70	0,69	0,68	0,72	0,67	0,71	0,71	0,82	0,64	0,68	0,66	0,82	0,67
JX879770.1	0,70	0,70	0,66	0,70	0,70	0,65	0,67	0,72	0,72	0,69	0,70	0,70	0,69	0,70	0,70	0,69	0,68	0,72	0,67	0,71	0,71	0,82	0,64	0,68	0,66	0,82	0,67
JX028271.1	0,66	0,67	0,64	0,66	0,66	0,65	0,64	0,70	0,67	0,63	0,66	0,66	0,66	0,64	0,64	0,65	0,64	0,67	0,95	0,79	0,87	0,67	0,63	0,79	0,93	0,67	0,66
KF705679.1	0,63	0,65	0,63	0,64	0,64	0,64	0,66	0,63	0,66	0,63	0,64	0,64	0,66	0,65	0,65	0,65	0,65	0,69	0,66	0,64	0,65	0,76	0,64	0,66	0,66	0,75	0,61
KF537001.1	0,64	0,65	0,61	0,64	0,64	0,63	0,61	0,66	0,67	0,61	0,64	0,64	0,64	0,62	0,62	0,64	0,63	0,65	0,79	1,00	0,80	0,67	0,62	0,86	0,77	0,67	0,63
KM102249.1	0,75	0,76	0,72	0,76	0,83	0,81	0,86	0,78	0,77	0,80	0,76	0,75	0,75	0,76	0,76	0,74	0,74	0,73	0,69	0,65	0,68	0,68	0,61	0,66	0,67	0,69	0,74
KR072623.1	0,82	0,79	0,75	0,79	0,73	0,71	0,67	0,81	0,74	0,79	0,79	0,79	0,78	0,80	0,80	0,79	0,78	0,74	0,62	0,62	0,65	0,63	0,62	0,63	0,63	0,64	0,76
KM361055.1	0,66	0,69	0,66	0,69	0,68	0,64	0,68	0,73	0,65	0,70	0,68	0,69	0,68	0,68	0,68	0,68	0,67	0,68	0,63	0,62	0,62	0,64	0,60	0,61	0,62	0,64	0,69
KM361056.1	0,66	0,69	0,66	0,69	0,68	0,64	0,68	0,73	0,65	0,70	0,68	0,69	0,68	0,68	0,68	0,68	0,67	0,68	0,63	0,62	0,62	0,64	0,60	0,61	0,62	0,64	0,69
KM361061.1	0,66	0,69	0,66	0,69	0,68	0,64	0,67	0,73	0,65	0,71	0,68	0,69	0,68	0,68	0,68	0,68	0,67	0,68	0,62	0,61	0,61	0,63	0,60	0,60	0,63	0,63	0,69
KT885041.1	0,72	0,71	0,67	0,71	0,70	0,63	0,65	0,73	0,71	0,68	0,70	0,71	0,70	0,70	0,70	0,69	0,68	0,70	0,67	0,68	0,68	0,81	0,63	0,67	0,66	0,81	0,66
KT885044.1	0,64	0,65	0,61	0,64	0,64	0,63	0,61	0,66	0,67	0,61	0,64	0,64	0,64	0,62	0,62	0,64	0,63	0,65	0,79	1,00	0,80	0,67	0,62	0,86	0,77	0,67	0,63
KT885047.1	0,66	0,67	0,64	0,67	0,68	0,63	0,67	0,69	0,68	0,66	0,67	0,67	0,66	0,65	0,65	0,67	0,67	0,69	0,67	0,67	0,67	0,99	0,66	0,68	0,66	0,96	0,67
KT885050.1	0,64	0,67	0,64	0,66	0,66	0,65	0,63	0,68	0,66	0,62	0,66	0,66	0,66	0,64	0,64	0,65	0,64	0,64	1,00	0,79	0,86	0,67	0,63	0,81	0,96	0,68	0,64
KT934965.1	0,66	0,67	0,64	0,67	0,68	0,63	0,67	0,69	0,68	0,66	0,67	0,67	0,66	0,65	0,65	0,67	0,67	0,69	0,67	0,67	0,67	0,99	0,66	0,68	0,66	0,96	0,67
KU215675.1	0,66	0,67	0,64	0,67	0,69	0,64	0,67	0,70	0,67	0,66	0,67	0,67	0,66	0,65	0,65	0,67	0,67	0,68	0,68	0,66	0,67	0,99	0,66	0,67	0,67	0,96	0,68
KX845680.1	0,78	0,78	0,73	0,79	0,75	0,76	0,72	0,79	0,76	0,80	0,78	0,79	0,77	0,79	0,79	0,78	0,77	0,76	0,66	0,66	0,66	0,68	0,63	0,68	0,65	0,68	0,82
KY594712.1	0,66	0,67	0,64	0,67	0,68	0,63	0,67	0,69	0,68	0,66	0,67	0,67	0,66	0,65	0,65	0,67	0,67	0,69	0,67	0,67	0,67	1,00	0,66	0,68	0,66	0,96	0,67
KY662269.1	0,67	0,71	0,67	0,70	0,77	0,79	0,78	0,76	0,74	0,70	0,70	0,69	0,70	0,70	0,70	0,70	0,69	0,69	0,69	0,69	0,68	0,64	0,62	0,68	0,66	0,66	0,74
KY662275.1	0,70	0,74	0,69	0,73	0,78	0,75	0,79	0,77	0,77	0,71	0,73	0,72	0,73	0,73	0,73	0,73	0,72	0,68	0,69	0,67	0,67	0,65	0,62	0,68	0,66	0,66	0,73
MG717393.1	0,64	0,66	0,62	0,65	0,64	0,64	0,62	0,67	0,70	0,64	0,64	0,65	0,64	0,64	0,64	0,64	0,64	0,67	0,80	0,95	0,78	0,70	0,65	0,87	0,77	0,70	0,66
KY978757.1	0,67	0,67	0,64	0,67	0,67	0,61	0,65	0,67	0,67	0,66	0,67	0,67	0,66	0,65	0,65	0,65	0,65	0,68	0,66	0,66	0,66	0,98	0,65	0,68	0,65	0,93	0,65
MN183135.1	0,66	0,66	0,63	0,67	0,66	0,64	0,66	0,68	0,68	0,62	0,67	0,67	0,65	0,65	0,65	0,66	0,65	0,64	0,87	0,80	0,97	0,69	0,66	0,82	0,86	0,70	0,62
MN850093.1	0,67	0,66	0,63	0,66	0,66	0,67	0,65	0,70	0,68	0,66	0,66	0,66	0,65	0,64	0,64	0,65	0,64	0,69	0,81	0,86	0,82	0,68	0,66	0,99	0,77	0,66	0,66
MN850096.1	0,67	0,66	0,63	0,66	0,66	0,67	0,65	0,70	0,68	0,66	0,66	0,66	0,65	0,64	0,64	0,65	0,64	0,69	0,82	0,86	0,82	0,68	0,66	0,98	0,78	0,66	0,66
MN639737.1	0,63	0,66	0,63	0,66	0,68	0,63	0,62	0,66	0,66	0,62	0,65	0,66	0,65	0,64	0,64	0,64	0,64	0,64	0,98	0,80	0,83	0,66	0,63	0,79	0,95	0,66	0,64
MN639740.1	0,63	0,66	0,63	0,66	0,68	0,63	0,62	0,66	0,66	0,62	0,65	0,66	0,65	0,64	0,64	0,64	0,64	0,64	0,98	0,80	0,83	0,66	0,63	0,79	0,95	0,66	0,64
MT024592.1	0,64	0,67	0,64	0,66	0,66	0,64	0,62	0,67	0,66	0,63	0,66	0,66	0,66	0,64	0,64	0,65	0,64	0,64	0,99	0,78	0,85	0,67	0,64	0,80	0,97	0,68	0,65
MT514291.1	0,63	0,66	0,63	0,66	0,68	0,64	0,62	0,66	0,66	0,61	0,65	0,66	0,65	0,64	0,64	0,64	0,64	0,64	0,98	0,81	0,84	0,66	0,62	0,80	0,94	0,66	0,63
MT514292.1	0,64	0,67	0,64	0,66	0,67	0,63	0,62	0,65	0,66	0,62	0,66	0,66	0,66	0,64	0,64	0,65	0,64	0,66	0,94	0,79	0,84	0,66	0,63	0,78	0,93	0,66	0,64
MT514294.1	0,64	0,67	0,64	0,66	0,66	0,64	0,62	0,67	0,66	0,63	0,66	0,66	0,66	0,64	0,64	0,65	0,64	0,64	0,99	0,78	0,85	0,67	0,64	0,80	0,97	0,68	0,65
MT514295.1	0,66	0,66	0,62	0,66	0,65	0,66	0,65	0,68	0,67	0,61	0,66	0,66	0,64	0,64	0,64	0,65	0,64	0,64	0,86	0,80	1,00	0,67	0,66	0,81	0,84	0,69	0,63
MT514296.1	0,66	0,66	0,63	0,67	0,66	0,64	0,66	0,68	0,68	0,62	0,67	0,67	0,65	0,65	0,65	0,66	0,65	0,64	0,87	0,80	0,98	0,69	0,66	0,82	0,86	0,70	0,62
MT514297.1	0,67	0,67	0,64	0,68	0,67	0,66	0,66	0,69	0,70	0,63	0,68	0,68	0,66	0,66	0,66	0,67	0,66	0,65	0,86	0,81	0,96	0,69	0,66	0,82	0,84	0,68	0,63
MT514298.1	0,66	0,66	0,62	0,66	0,65	0,66	0,65	0,68	0,67	0,61	0,66	0,66	0,64	0,64	0,64	0,65	0,64	0,64	0,86	0,80	1,00	0,67	0,66	0,81	0,84	0,69	0,63
MN258157.1	0,67	0,66	0,63	0,66	0,66	0,67	0,65	0,70	0,68	0,66	0,66	0,66	0,65	0,64	0,64	0,65	0,64	0,69	0,81	0,86	0,82	0,68	0,66	0,99	0,77	0,66	0,66
MN832779.1	0,64	0,67	0,64	0,66	0,65	0,64	0,62	0,66	0,66	0,64	0,66	0,66	0,66	0,64	0,64	0,65	0,64	0,64	0,97	0,77	0,85	0,67	0,64	0,78	0,99	0,68	0,65
MN832782.1	0,64	0,67	0,64	0,66	0,66	0,64	0,62	0,67	0,66	0,63	0,66	0,66	0,66	0,64	0,64	0,65	0,64	0,64	0,99	0,78	0,85	0,67	0,64	0,80	0,97	0,68	0,65
MT648514.1	0,69	0,70	0,67	0,69	0,65	0,69	0,66	0,69	0,65	0,68	0,69	0,69	0,69	0,69	0,69	0,68	0,66	0,69	0,60	0,60	0,61	0,61	0,59	0,61	0,58	0,61	0,69
OM030302.1	0,78	0,78	0,73	0,77	0,73	0,73	0,71	0,79	0,72	0,76	0,77	0,77	0,77	0,77	0,77	0,75	0,74	0,77	0,68	0,67	0,68	0,68	0,65	0,67	0,66	0,69	0,78
OM030305.1	0,69	0,70	0,66	0,70	0,68	0,62	0,65	0,71	0,66	0,67	0,70	0,70	0,69	0,68	0,68	0,70	0,69	0,67	0,67	0,66	0,66	0,94	0,63	0,66	0,66	0,96	0,66
OM030311.1	0,64	0,66	0,64	0,66	0,68	0,64	0,65	0,66	0,69	0,64	0,65	0,66	0,65	0,65	0,65	0,64	0,64	0,66	0,68	0,67	0,66	0,79	0,65	0,67	0,68	0,78	0,62

OK422869.1	0,71	0,70	0,68	0,70	0,69	0,64	0,66	0,72	0,70	0,67	0,70	0,70	0,69	0,70	0,70	0,68	0,67	0,69	0,66	0,67	0,67	0,80	0,62	0,66	0,66	0,80	0,66
MZ504241.1	0,71	0,71	0,67	0,71	0,70	0,65	0,68	0,74	0,72	0,70	0,70	0,71	0,70	0,70	0,70	0,70	0,70	0,71	0,66	0,71	0,70	0,82	0,64	0,67	0,66	0,83	0,69
OQ092243.1	0,64	0,64	0,60	0,64	0,65	0,64	0,62	0,68	0,66	0,66	0,64	0,64	0,63	0,62	0,62	0,63	0,62	0,66	0,78	0,86	0,78	0,66	0,63	0,93	0,77	0,64	0,65
OR148904.1	0,69	0,68	0,64	0,67	0,67	0,66	0,63	0,68	0,68	0,62	0,67	0,67	0,67	0,65	0,65	0,66	0,65	0,69	0,81	0,89	0,82	0,69	0,64	0,87	0,78	0,67	0,66
OR365538.1	0,50	0,52	0,52	0,52	0,52	0,54	0,51	0,54	0,58	0,55	0,52	0,52	0,50	0,51	0,51	0,50	0,50	0,56	0,63	0,70	0,64	0,55	0,53	0,72	0,61	0,54	0,56
NC_005235.1	0,72	0,71	0,67	0,71	0,70	0,63	0,65	0,73	0,71	0,68	0,70	0,71	0,70	0,70	0,70	0,69	0,68	0,70	0,67	0,68	0,68	0,81	0,63	0,67	0,66	0,81	0,66
NC_034401.1	0,76	0,78	0,72	0,78	0,79	0,71	0,75	0,78	0,78	0,75	0,78	0,78	0,77	0,78	0,78	0,78	0,78	0,77	0,69	0,64	0,66	0,69	0,63	0,66	0,66	0,68	0,75
NC_034399.1	0,68	0,70	0,68	0,71	0,68	0,66	0,69	0,71	0,74	0,66	0,70	0,71	0,69	0,69	0,69	0,70	0,69	0,68	0,70	0,66	0,68	0,77	0,68	0,69	0,68	0,77	0,68
NC_034402.1	0,66	0,66	0,62	0,66	0,67	0,66	0,68	0,69	0,66	0,70	0,65	0,66	0,64	0,65	0,65	0,64	0,64	0,67	0,66	0,68	0,68	0,70	0,69	0,69	0,66	0,70	0,70
NC_034403.1	0,66	0,66	0,63	0,66	0,65	0,61	0,61	0,66	0,64	0,61	0,65	0,66	0,65	0,64	0,64	0,64	0,64	0,67	0,82	0,85	0,82	0,71	0,66	0,85	0,82	0,74	0,65
NC_034407.1	0,64	0,68	0,64	0,67	0,70	0,67	0,70	0,67	0,70	0,67	0,67	0,67	0,67	0,67	0,67	0,66	0,65	0,68	0,72	0,67	0,70	0,72	0,67	0,68	0,72	0,72	0,72
NC_034467.1	0,68	0,70	0,66	0,69	0,65	0,63	0,65	0,69	0,70	0,64	0,69	0,69	0,69	0,67	0,67	0,68	0,67	0,69	0,81	0,79	0,80	0,73	0,66	0,81	0,81	0,72	0,66
NC_034485.1	0,68	0,68	0,66	0,67	0,69	0,66	0,68	0,67	0,69	0,65	0,67	0,67	0,67	0,66	0,66	0,66	0,65	0,69	0,69	0,69	0,69	0,81	0,66	0,70	0,68	0,80	0,65
NC_034515.1	0,67	0,67	0,64	0,66	0,65	0,65	0,62	0,68	0,68	0,62	0,66	0,66	0,66	0,64	0,64	0,65	0,64	0,67	0,81	0,88	0,81	0,66	0,63	0,87	0,79	0,66	0,65
NC_034517.1	0,71	0,71	0,68	0,71	0,70	0,66	0,66	0,72	0,72	0,68	0,70	0,71	0,70	0,70	0,70	0,70	0,70	0,71	0,68	0,70	0,69	0,84	0,66	0,69	0,67	0,82	0,71
NC_034519.1	0,67	0,68	0,64	0,67	0,66	0,64	0,65	0,70	0,69	0,64	0,67	0,67	0,67	0,65	0,65	0,66	0,65	0,69	0,92	0,82	0,88	0,70	0,66	0,83	0,91	0,70	0,65
NC_034556.1	0,70	0,69	0,67	0,69	0,72	0,68	0,73	0,71	0,71	0,74	0,69	0,69	0,68	0,69	0,69	0,67	0,66	0,72	0,66	0,68	0,71	0,82	0,66	0,69	0,66	0,81	0,70
NC_034560.1	0,70	0,71	0,67	0,70	0,71	0,66	0,71	0,70	0,74	0,66	0,70	0,70	0,70	0,69	0,69	0,69	0,68	0,70	0,71	0,67	0,69	0,80	0,70	0,71	0,69	0,79	0,68
NC_034564.1	0,73	0,74	0,70	0,74	0,76	0,72	0,71	0,78	0,69	0,77	0,74	0,74	0,74	0,74	0,74	0,73	0,72	0,74	0,64	0,61	0,64	0,65	0,65	0,66	0,64	0,65	0,86
NC_038529.1	0,62	0,66	0,63	0,66	0,64	0,62	0,64	0,66	0,67	0,62	0,65	0,66	0,65	0,64	0,64	0,64	0,64	0,64	0,80	0,73	0,79	0,68	0,65	0,77	0,78	0,67	0,63
NC_043068.1	0,69	0,69	0,67	0,68	0,70	0,68	0,72	0,71	0,71	0,66	0,68	0,68	0,68	0,67	0,67	0,67	0,66	0,69	0,72	0,66	0,69	0,77	0,69	0,68	0,71	0,77	0,66
NC_043175.1	0,63	0,66	0,62	0,65	0,66	0,69	0,68	0,69	0,70	0,67	0,64	0,65	0,65	0,65	0,65	0,65	0,64	0,66	0,66	0,66	0,66	0,77	0,69	0,66	0,66	0,77	0,66
NC_043407.1	0,66	0,65	0,62	0,65	0,66	0,66	0,62	0,66	0,66	0,65	0,65	0,65	0,64	0,65	0,65	0,64	0,64	0,68	0,75	0,84	0,80	0,66	0,66	0,87	0,75	0,67	0,67
NC_055147.1	0,64	0,66	0,64	0,66	0,70	0,66	0,69	0,68	0,67	0,64	0,65	0,66	0,64	0,65	0,65	0,64	0,64	0,66	0,70	0,64	0,66	0,75	0,65	0,66	0,67	0,76	0,66
NC_055632.1	0,72	0,74	0,71	0,73	0,78	0,70	0,75	0,75	0,77	0,73	0,73	0,72	0,73	0,73	0,73	0,72	0,71	0,74	0,66	0,66	0,64	0,68	0,60	0,66	0,63	0,67	0,69
NC_055636.1	0,68	0,69	0,65	0,68	0,67	0,66	0,67	0,70	0,69	0,66	0,68	0,68	0,68	0,66	0,66	0,67	0,67	0,69	0,87	0,81	0,89	0,71	0,67	0,84	0,86	0,71	0,66
NC_078485.1	0,66	0,69	0,66	0,69	0,68	0,64	0,68	0,73	0,65	0,70	0,68	0,69	0,68	0,68	0,68	0,68	0,67	0,68	0,63	0,62	0,62	0,64	0,60	0,61	0,62	0,64	0,69

	DQ989237.1	EF397003.1	EF646763.1	EU788002.1	GU140096.1	FJ858378.1	GQ244526.1	HM015220.1	JQ026206.1	JQ083393.1	JN831945.1	JX853574.1	JX879770.1	JX028271.1	KF705679.1	KF537001.1	KM102249.1	KR072623.1	KM361055.1	KM361056.1	KM361061.1	KT885041.1	KT885044.1	KT885047.1	KT885050.1	KT934965.1	KU215675.1
Mamanguape_virus_EM_725	0,68	0,66	0,66	0,66	0,66	0,67	0,67	0,62	0,72	0,66	0,64	0,70	0,70	0,66	0,63	0,64	0,75	0,82	0,66	0,66	0,66	0,72	0,64	0,66	0,64	0,66	0,66
Mamanguape_virus_EM_711	0,68	0,66	0,69	0,66	0,67	0,69	0,68	0,64	0,71	0,67	0,67	0,70	0,70	0,67	0,65	0,65	0,76	0,79	0,69	0,69	0,69	0,71	0,65	0,67	0,67	0,67	0,67
Mamanguape_virus_EM_708	0,64	0,62	0,65	0,62	0,64	0,65	0,64	0,60	0,67	0,64	0,64	0,66	0,66	0,64	0,63	0,61	0,72	0,75	0,66	0,66	0,66	0,67	0,61	0,64	0,64	0,64	0,64
OR684449.1	0,68	0,66	0,68	0,66	0,67	0,68	0,67	0,64	0,71	0,67	0,66	0,70	0,70	0,66	0,64	0,64	0,76	0,79	0,69	0,69	0,69	0,71	0,64	0,67	0,66	0,67	0,67
MG663536.1	0,66	0,66	0,69	0,66	0,69	0,67	0,65	0,67	0,70	0,68	0,66	0,70	0,70	0,66	0,64	0,64	0,83	0,73	0,68	0,68	0,68	0,70	0,64	0,68	0,66	0,68	0,69
JN037851.1	0,61	0,68	0,65	0,66	0,64	0,70	0,69	0,62	0,64	0,63	0,64	0,65	0,65	0,65	0,64	0,63	0,81	0,71	0,64	0,64	0,64	0,63	0,63	0,63	0,65	0,63	0,64
KT316176.1	0,63	0,64	0,66	0,62	0,67	0,66	0,66	0,63	0,66	0,67	0,62	0,67	0,67	0,64	0,66	0,61	0,86	0,67	0,68	0,68	0,67	0,65	0,61	0,67	0,63	0,67	0,67
JQ287716.2	0,69	0,68	0,69	0,65	0,70	0,70	0,69	0,70	0,73	0,69	0,67	0,72	0,72	0,70	0,63	0,66	0,78	0,81	0,73	0,73	0,73	0,73	0,66	0,69	0,68	0,69	0,70
OP122967.1	0,65	0,69	0,70	0,68	0,67	0,69	0,70	0,63	0,71	0,68	0,66	0,72	0,72	0,67	0,66	0,67	0,77	0,74	0,65	0,65	0,65	0,71	0,67	0,68	0,66	0,68	0,67
JX465369.1	0,66	0,66	0,64	0,64	0,66	0,66	0,68	0,65	0,68	0,66	0,63	0,69	0,69	0,63	0,63	0,61	0,80	0,79	0,70	0,70	0,71	0,68	0,61	0,66	0,62	0,66	0,66
Mamanguape_virus_EM_709	0,68	0,65	0,68	0,65	0,67	0,68	0,67	0,64	0,70	0,67	0,66	0,70	0,70	0,66	0,64	0,64	0,76	0,79	0,68	0,68	0,68	0,70	0,64	0,67	0,66	0,67	0,67
Mamanguape_virus_EM_707	0,68	0,66	0,68	0,66	0,67	0,68	0,67	0,64	0,71	0,67	0,66	0,70	0,70	0,66	0,64	0,64	0,75	0,79	0,69	0,69	0,69	0,71	0,64	0,67	0,66	0,67	0,67
Hantavirus_HantaV1_KX442773_1	0,67	0,64	0,68	0,64	0,66	0,68	0,67	0,63	0,70	0,66	0,66	0,69	0,69	0,66	0,66	0,64	0,75	0,78	0,68	0,68	0,68	0,70	0,64	0,66	0,66	0,66	0,66
Hantavirus_HantaV1_KX442793_1	0,66	0,65	0,66	0,64	0,65	0,66	0,65	0,64	0,70	0,65	0,64	0,70	0,70	0,64	0,65	0,62	0,76	0,80	0,68	0,68	0,68	0,70	0,62	0,65	0,64	0,65	0,65

Hantavirus_HantaV1_KX442794_1	0,66	0,65	0,66	0,64	0,65	0,66	0,65	0,64	0,70	0,65	0,64	0,70	0,70	0,64	0,65	0,62	0,76	0,80	0,68	0,68	0,68	0,70	0,62	0,65	0,64	0,65	0,65
Hantavirus_HantaV2_KX442772_1	0,68	0,64	0,67	0,64	0,67	0,67	0,66	0,62	0,69	0,67	0,65	0,69	0,69	0,65	0,65	0,64	0,74	0,79	0,68	0,68	0,68	0,69	0,64	0,67	0,65	0,67	0,67
Hantavirus_HantaV2_KX442771_1	0,67	0,64	0,66	0,64	0,67	0,66	0,66	0,62	0,68	0,67	0,64	0,68	0,68	0,64	0,65	0,63	0,74	0,78	0,67	0,67	0,67	0,68	0,63	0,67	0,64	0,67	0,67
Hantavirus_HantaV3_KX442770_1	0,65	0,70	0,67	0,67	0,68	0,70	0,70	0,64	0,70	0,69	0,64	0,72	0,72	0,67	0,69	0,65	0,73	0,74	0,68	0,68	0,68	0,70	0,65	0,69	0,64	0,69	0,68
EF405801.1	0,66	0,78	0,87	0,77	0,68	0,82	0,80	0,77	0,68	0,67	0,99	0,67	0,67	0,95	0,66	0,79	0,69	0,62	0,63	0,63	0,62	0,67	0,79	0,67	1,00	0,67	0,68
NC_005217.1	0,66	0,86	0,78	0,87	0,66	0,83	0,83	0,71	0,67	0,67	0,78	0,71	0,71	0,79	0,64	1,00	0,65	0,62	0,62	0,62	0,61	0,68	1,00	0,67	0,79	0,67	0,66
NC_005226.1	0,66	0,80	0,89	0,81	0,67	0,81	0,78	0,75	0,70	0,67	0,85	0,71	0,71	0,87	0,65	0,80	0,68	0,65	0,62	0,62	0,61	0,68	0,80	0,67	0,86	0,67	0,67
NC_006435.1	0,92	0,66	0,70	0,64	0,99	0,69	0,70	0,64	0,81	1,00	0,67	0,82	0,82	0,67	0,76	0,67	0,68	0,63	0,64	0,64	0,63	0,81	0,67	0,99	0,67	0,99	0,99
NC_055170.1	0,62	0,66	0,64	0,62	0,66	0,66	0,65	0,66	0,64	0,66	0,64	0,64	0,64	0,63	0,64	0,62	0,61	0,62	0,60	0,60	0,60	0,63	0,62	0,66	0,63	0,66	0,66
AF291704.5	0,66	0,89	0,79	0,88	0,67	0,87	0,87	0,73	0,66	0,68	0,80	0,68	0,68	0,79	0,66	0,86	0,66	0,63	0,61	0,61	0,60	0,67	0,86	0,68	0,81	0,68	0,67
AY526217.1	0,65	0,77	0,86	0,77	0,67	0,80	0,78	0,74	0,67	0,66	0,97	0,66	0,66	0,93	0,66	0,77	0,67	0,63	0,62	0,62	0,63	0,66	0,77	0,66	0,96	0,66	0,67
DQ371906.1	0,93	0,66	0,71	0,63	0,96	0,70	0,69	0,65	0,82	0,96	0,68	0,82	0,82	0,67	0,75	0,67	0,69	0,64	0,64	0,64	0,63	0,81	0,67	0,96	0,68	0,96	0,96
DQ825770.1	0,66	0,69	0,64	0,65	0,68	0,69	0,68	0,65	0,68	0,67	0,65	0,67	0,67	0,66	0,61	0,63	0,74	0,76	0,69	0,69	0,69	0,66	0,63	0,67	0,64	0,67	0,68
DQ989237.1	1,00	0,65	0,68	0,61	0,93	0,69	0,68	0,61	0,81	0,92	0,66	0,80	0,80	0,65	0,73	0,66	0,67	0,66	0,62	0,62	0,61	0,81	0,66	0,92	0,66	0,92	0,93
EF397003.1	0,65	1,00	0,80	0,87	0,66	0,87	0,87	0,75	0,67	0,66	0,77	0,71	0,71	0,79	0,65	0,86	0,68	0,66	0,62	0,62	0,61	0,68	0,86	0,67	0,78	0,67	0,66
EF646763.1	0,68	0,80	1,00	0,79	0,70	0,83	0,81	0,79	0,71	0,70	0,87	0,71	0,71	0,88	0,68	0,78	0,70	0,66	0,66	0,66	0,66	0,70	0,78	0,70	0,87	0,70	0,70
EU788002.1	0,61	0,87	0,79	1,00	0,63	0,86	0,84	0,69	0,65	0,64	0,78	0,67	0,67	0,78	0,62	0,87	0,66	0,63	0,61	0,61	0,61	0,65	0,87	0,64	0,77	0,64	0,63
GU140096.1	0,93	0,66	0,70	0,63	1,00	0,70	0,71	0,64	0,82	0,99	0,68	0,81	0,81	0,68	0,76	0,66	0,69	0,64	0,64	0,64	0,63	0,82	0,66	0,98	0,68	0,98	1,00
FJ858378.1	0,69	0,87	0,83	0,86	0,70	1,00	0,96	0,74	0,69	0,69	0,81	0,68	0,68	0,82	0,68	0,83	0,71	0,67	0,65	0,65	0,64	0,69	0,83	0,69	0,82	0,69	0,70
GQ244526.1	0,68	0,87	0,81	0,84	0,71	0,96	1,00	0,74	0,67	0,70	0,79	0,66	0,66	0,80	0,70	0,83	0,69	0,66	0,65	0,65	0,64	0,67	0,83	0,70	0,80	0,70	0,71
HM015220.1	0,61	0,75	0,79	0,69	0,64	0,74	0,74	1,00	0,71	0,64	0,76	0,69	0,69	0,77	0,63	0,71	0,67	0,65	0,62	0,62	0,61	0,69	0,71	0,64	0,77	0,64	0,64
JQ026206.1	0,81	0,67	0,71	0,65	0,82	0,69	0,67	0,71	1,00	0,81	0,68	0,87	0,87	0,69	0,75	0,67	0,71	0,70	0,62	0,62	0,61	0,98	0,67	0,81	0,68	0,81	0,82
JQ083393.1	0,92	0,66	0,70	0,64	0,99	0,69	0,70	0,64	0,81	1,00	0,67	0,82	0,82	0,67	0,76	0,67	0,68	0,63	0,64	0,64	0,63	0,81	0,67	0,99	0,67	0,99	0,99
JN831945.1	0,66	0,77	0,87	0,78	0,68	0,81	0,79	0,76	0,68	0,67	1,00	0,67	0,67	0,94	0,66	0,78	0,68	0,63	0,62	0,62	0,63	0,67	0,78	0,67	0,99	0,67	0,68
JX853574.1	0,80	0,71	0,71	0,67	0,81	0,68	0,66	0,69	0,87	0,82	0,67	1,00	1,00	0,67	0,73	0,71	0,70	0,66	0,62	0,62	0,63	0,86	0,71	0,82	0,67	0,82	0,81
JX879770.1	0,80	0,71	0,71	0,67	0,81	0,68	0,66	0,69	0,87	0,82	0,67	1,00	1,00	0,67	0,73	0,71	0,70	0,66	0,62	0,62	0,63	0,86	0,71	0,82	0,67	0,82	0,81
JX028271.1	0,65	0,79	0,88	0,78	0,68	0,82	0,80	0,77	0,69	0,67	0,94	0,67	0,67	1,00	0,66	0,79	0,68	0,65	0,66	0,66	0,65	0,69	0,79	0,66	0,95	0,66	0,68
KF705679.1	0,73	0,65	0,68	0,62	0,76	0,68	0,70	0,63	0,75	0,76	0,66	0,73	0,73	0,66	1,00	0,64	0,65	0,63	0,62	0,62	0,61	0,75	0,64	0,76	0,66	0,76	0,76
KF537001.1	0,66	0,86	0,78	0,87	0,66	0,83	0,83	0,71	0,67	0,67	0,78	0,71	0,71	0,79	0,64	1,00	0,65	0,62	0,62	0,62	0,61	0,68	1,00	0,67	0,79	0,67	0,66
KM102249.1	0,67	0,68	0,70	0,66	0,69	0,71	0,69	0,67	0,71	0,68	0,68	0,70	0,70	0,68	0,65	0,65	1,00	0,72	0,67	0,67	0,65	0,70	0,65	0,69	0,69	0,69	0,69
KR072623.1	0,66	0,66	0,66	0,63	0,64	0,67	0,66	0,65	0,70	0,63	0,63	0,66	0,66	0,65	0,63	0,62	0,72	1,00	0,70	0,70	0,70	0,69	0,62	0,63	0,62	0,63	0,64
KM361055.1	0,62	0,62	0,66	0,61	0,64	0,65	0,65	0,62	0,62	0,64	0,62	0,62	0,62	0,66	0,62	0,62	0,67	0,70	1,00	1,00	0,98	0,62	0,62	0,64	0,63	0,64	0,64
KM361056.1	0,62	0,62	0,66	0,61	0,64	0,65	0,65	0,62	0,62	0,64	0,62	0,62	0,62	0,66	0,62	0,62	0,67	0,70	1,00	1,00	0,98	0,62	0,62	0,64	0,63	0,64	0,64
KM361061.1	0,61	0,61	0,66	0,61	0,63	0,64	0,64	0,61	0,61	0,63	0,63	0,63	0,63	0,65	0,61	0,61	0,65	0,70	0,98	0,98	1,00	0,61	0,61	0,63	0,62	0,63	0,63
KT885041.1	0,81	0,68	0,70	0,65	0,82	0,69	0,67	0,69	0,98	0,81	0,67	0,86	0,86	0,69	0,75	0,68	0,70	0,69	0,62	0,62	0,61	1,00	0,68	0,80	0,67	0,80	0,82
KT885044.1	0,66	0,86	0,78	0,87	0,66	0,83	0,83	0,71	0,67	0,67	0,78	0,71	0,71	0,79	0,64	1,00	0,65	0,62	0,62	0,62	0,61	0,68	1,00	0,67	0,79	0,67	0,66
KT885047.1	0,92	0,67	0,70	0,64	0,98	0,69	0,70	0,64	0,81	0,99	0,67	0,82	0,82	0,66	0,76	0,67	0,69	0,63	0,64	0,64	0,63	0,80	0,67	1,00	0,67	1,00	0,98
KT885050.1	0,66	0,78	0,87	0,77	0,68	0,82	0,80	0,77	0,68	0,67	0,99	0,67	0,67	0,95	0,66	0,79	0,69	0,62	0,63	0,63	0,62	0,67	0,79	0,67	1,00	0,67	0,68
KT934965.1	0,92	0,67	0,70	0,64	0,98	0,69	0,70	0,64	0,81	0,99	0,67	0,82	0,82	0,66	0,76	0,67	0,69	0,63	0,64	0,64	0,63	0,80	0,67	1,00	0,67	1,00	0,98
KU215675.1	0,93	0,66	0,70	0,63	1,00	0,70	0,71	0,64	0,82	0,99	0,68	0,81	0,81	0,68	0,76	0,66	0,69	0,64	0,64	0,64	0,63	0,82	0,66	0,98	0,68	0,98	1,00
KX845680.1	0,67	0,70	0,67	0,67	0,68	0,72	0,72	0,66	0,69	0,68	0,65	0,66	0,66	0,67	0,66	0,66	0,74	0,79	0,74	0,74	0,72	0,68	0,66	0,68	0,66	0,68	0,68
KY594712.1	0,92	0,66	0,70	0,64	0,99	0,69	0,70	0,64	0,81	1,00	0,67	0,82	0,82	0,67	0,76	0,67	0,68	0,63	0,64	0,64	0,63	0,81	0,67	0,99	0,67	0,99	0,99
KY662269.1	0,63	0,67	0,68	0,67	0,65	0,71	0,70	0,65	0,68	0,64	0,68	0,70	0,70	0,68	0,64	0,69	0,81	0,67	0,65	0,65	0,65	0,67	0,69	0,64	0,69	0,64	0,65
KY662275.1	0,62	0,66	0,69	0,66	0,65	0,70	0,68	0,66	0,67	0,65	0,68	0,69	0,69	0,68	0,64	0,67	0,81	0,67	0,67	0,67	0,67	0,67	0,67	0,64	0,69	0,64	0,65
MG717393.1	0,68	0,87	0,79	0,85	0,69	0,85	0,84	0,72	0,70	0,70	0,79	0,71	0,71	0,79	0,66	0,95	0,66	0,64	0,63	0,63	0,62	0,71	0,95	0,70	0,80	0,70	0,69
KY978757.1	0,92	0,66	0,68	0,63	0,97	0,68	0,69	0,63	0,80	0,98	0,66	0,81	0,81	0,66	0,75	0,66	0,66	0,61	0,63	0,63	0,62	0,82	0,66	0,97	0,66	0,97	0,97
MN183135.1	0,66	0,79	0,88	0,79	0,69	0,81	0,79	0,76	0,69	0,69	0,87	0,70	0,70	0,88	0,66	0,80	0,68	0,63	0,64	0,64	0,63	0,69	0,80	0,68	0,87	0,68	0,69

MN850093.1	0,66	0,88	0,80	0,89	0,67	0,87	0,87	0,73	0,66	0,68	0,80	0,68	0,68	0,79	0,66	0,86	0,66	0,63	0,61	0,61	0,60	0,67	0,86	0,68	0,81	0,68	0,67
MN850096.1	0,66	0,88	0,80	0,88	0,67	0,87	0,87	0,73	0,66	0,68	0,81	0,68	0,68	0,79	0,66	0,86	0,66	0,63	0,61	0,61	0,60	0,67	0,86	0,68	0,82	0,68	0,67
MN639737.1	0,64	0,77	0,85	0,80	0,67	0,79	0,78	0,74	0,66	0,66	0,98	0,66	0,66	0,92	0,66	0,80	0,66	0,61	0,62	0,62	0,63	0,66	0,80	0,66	0,98	0,66	0,67
MN639740.1	0,64	0,77	0,85	0,80	0,67	0,79	0,78	0,74	0,66	0,66	0,98	0,66	0,66	0,92	0,66	0,80	0,66	0,61	0,62	0,62	0,63	0,66	0,80	0,66	0,98	0,66	0,67
MT024592.1	0,66	0,77	0,87	0,78	0,68	0,81	0,79	0,76	0,68	0,67	1,00	0,67	0,67	0,94	0,66	0,78	0,68	0,63	0,62	0,62	0,63	0,67	0,78	0,67	0,99	0,67	0,68
MT514291.1	0,64	0,77	0,85	0,79	0,67	0,80	0,79	0,75	0,66	0,66	0,98	0,66	0,66	0,93	0,66	0,81	0,67	0,61	0,63	0,63	0,62	0,66	0,81	0,66	0,98	0,66	0,67
MT514292.1	0,65	0,76	0,86	0,80	0,67	0,79	0,78	0,72	0,67	0,66	0,95	0,66	0,66	0,91	0,66	0,79	0,67	0,62	0,62	0,62	0,63	0,66	0,79	0,66	0,94	0,66	0,67
MT514294.1	0,66	0,77	0,87	0,78	0,68	0,81	0,79	0,76	0,68	0,67	1,00	0,67	0,67	0,94	0,66	0,78	0,68	0,63	0,62	0,62	0,63	0,67	0,78	0,67	0,99	0,67	0,68
MT514295.1	0,66	0,80	0,89	0,81	0,67	0,81	0,78	0,75	0,70	0,67	0,85	0,71	0,71	0,87	0,65	0,80	0,68	0,65	0,62	0,62	0,61	0,68	0,80	0,67	0,86	0,67	0,67
MT514296.1	0,66	0,79	0,88	0,79	0,69	0,81	0,79	0,76	0,69	0,69	0,87	0,70	0,70	0,88	0,66	0,80	0,68	0,63	0,64	0,64	0,63	0,69	0,80	0,68	0,87	0,68	0,69
MT514297.1	0,66	0,81	0,90	0,82	0,68	0,82	0,79	0,77	0,68	0,69	0,85	0,70	0,70	0,88	0,65	0,81	0,70	0,64	0,64	0,64	0,63	0,68	0,81	0,68	0,86	0,68	0,68
MT514298.1	0,66	0,80	0,89	0,81	0,67	0,81	0,78	0,75	0,70	0,67	0,85	0,71	0,71	0,87	0,65	0,80	0,68	0,65	0,62	0,62	0,61	0,68	0,80	0,67	0,86	0,67	0,67
MN258157.1	0,66	0,88	0,80	0,89	0,67	0,87	0,87	0,73	0,66	0,68	0,80	0,68	0,68	0,79	0,66	0,86	0,66	0,63	0,61	0,61	0,60	0,67	0,86	0,68	0,81	0,68	0,67
MN832779.1	0,66	0,77	0,87	0,78	0,68	0,81	0,79	0,75	0,68	0,67	0,98	0,67	0,67	0,94	0,66	0,77	0,68	0,64	0,63	0,63	0,64	0,67	0,77	0,67	0,97	0,67	0,68
MN832782.1	0,66	0,77	0,87	0,78	0,68	0,81	0,79	0,76	0,68	0,67	1,00	0,67	0,67	0,94	0,66	0,78	0,68	0,63	0,62	0,62	0,63	0,67	0,78	0,67	0,99	0,67	0,68
MT648514.1	0,61	0,61	0,62	0,61	0,61	0,63	0,62	0,58	0,61	0,61	0,59	0,62	0,62	0,61	0,63	0,60	0,65	0,71	0,87	0,87	0,86	0,61	0,60	0,61	0,60	0,61	0,61
OM030302.1	0,67	0,71	0,69	0,66	0,68	0,71	0,71	0,68	0,71	0,68	0,67	0,71	0,71	0,70	0,66	0,67	0,74	0,74	0,67	0,67	0,66	0,71	0,67	0,68	0,68	0,68	0,68
OM030305.1	0,98	0,66	0,69	0,62	0,95	0,70	0,69	0,63	0,82	0,94	0,67	0,82	0,82	0,66	0,75	0,66	0,69	0,66	0,64	0,64	0,63	0,82	0,66	0,94	0,67	0,94	0,95
OM030311.1	0,75	0,68	0,69	0,66	0,78	0,68	0,67	0,63	0,78	0,79	0,68	0,75	0,75	0,66	0,81	0,67	0,65	0,64	0,66	0,66	0,65	0,77	0,67	0,79	0,68	0,79	0,78
OK422869.1	0,80	0,67	0,69	0,64	0,81	0,68	0,66	0,69	0,97	0,80	0,66	0,86	0,86	0,68	0,76	0,67	0,69	0,68	0,61	0,61	0,61	0,98	0,67	0,79	0,66	0,79	0,81
MZ504241.1	0,81	0,71	0,71	0,66	0,82	0,67	0,66	0,69	0,87	0,82	0,66	0,98	0,98	0,66	0,72	0,71	0,71	0,68	0,63	0,63	0,64	0,86	0,71	0,83	0,66	0,83	0,82
OQ092243.1	0,63	0,86	0,77	0,87	0,65	0,83	0,84	0,71	0,65	0,66	0,79	0,67	0,67	0,77	0,65	0,86	0,64	0,61	0,60	0,60	0,61	0,66	0,86	0,66	0,78	0,66	0,65
OR148904.1	0,67	0,85	0,78	0,86	0,70	0,84	0,85	0,71	0,69	0,69	0,80	0,68	0,68	0,83	0,69	0,89	0,66	0,65	0,63	0,63	0,62	0,71	0,89	0,68	0,81	0,68	0,70
OR365538.1	0,53	0,81	0,65	0,70	0,54	0,71	0,71	0,58	0,55	0,55	0,62	0,58	0,58	0,63	0,51	0,70	0,55	0,51	0,50	0,50	0,49	0,56	0,70	0,55	0,63	0,55	0,54
NC_005235.1	0,81	0,68	0,70	0,65	0,82	0,69	0,67	0,70	0,98	0,81	0,67	0,87	0,87	0,69	0,75	0,68	0,70	0,69	0,62	0,62	0,61	0,98	0,68	0,80	0,67	0,80	0,82
NC_034401.1	0,65	0,66	0,68	0,61	0,69	0,68	0,67	0,67	0,68	0,69	0,68	0,68	0,68	0,69	0,62	0,64	0,78	0,73	0,72	0,72	0,70	0,69	0,64	0,68	0,69	0,68	0,69
NC_034399.1	0,73	0,66	0,69	0,65	0,78	0,68	0,68	0,64	0,74	0,77	0,70	0,75	0,75	0,68	0,77	0,66	0,67	0,65	0,62	0,62	0,63	0,73	0,66	0,77	0,70	0,77	0,78
NC_034402.1	0,71	0,71	0,67	0,66	0,70	0,66	0,67	0,65	0,71	0,70	0,66	0,74	0,74	0,66	0,71	0,68	0,67	0,66	0,62	0,62	0,63	0,71	0,68	0,70	0,66	0,70	0,70
NC_034403.1	0,71	0,82	0,81	0,83	0,71	0,84	0,82	0,73	0,71	0,71	0,83	0,71	0,71	0,81	0,71	0,85	0,66	0,64	0,62	0,62	0,62	0,72	0,85	0,71	0,82	0,71	0,71
NC_034407.1	0,71	0,69	0,71	0,68	0,73	0,72	0,72	0,66	0,72	0,72	0,72	0,69	0,69	0,72	0,73	0,67	0,71	0,63	0,69	0,69	0,68	0,71	0,67	0,72	0,72	0,72	0,73
NC_034467.1	0,71	0,81	0,84	0,77	0,73	0,82	0,82	0,76	0,71	0,73	0,80	0,72	0,72	0,83	0,71	0,79	0,66	0,66	0,66	0,66	0,66	0,73	0,79	0,72	0,81	0,72	0,73
NC_034485.1	0,79	0,69	0,71	0,66	0,81	0,69	0,69	0,63	0,79	0,81	0,69	0,79	0,79	0,69	0,88	0,69	0,66	0,67	0,66	0,66	0,65	0,80	0,69	0,81	0,69	0,81	0,81
NC_034515.1	0,66	0,86	0,81	0,88	0,66	0,88	0,87	0,73	0,68	0,66	0,80	0,68	0,68	0,82	0,66	0,88	0,66	0,65	0,63	0,63	0,63	0,68	0,88	0,66	0,81	0,66	0,66
NC_034517.1	0,81	0,71	0,71	0,69	0,83	0,71	0,70	0,69	0,86	0,84	0,68	0,87	0,87	0,69	0,77	0,70	0,69	0,70	0,64	0,64	0,63	0,84	0,70	0,84	0,68	0,84	0,83
NC_034519.1	0,67	0,82	0,91	0,80	0,69	0,84	0,82	0,79	0,71	0,70	0,92	0,71	0,71	0,93	0,68	0,82	0,68	0,66	0,66	0,66	0,65	0,69	0,82	0,70	0,92	0,70	0,69
NC_034556.1	0,79	0,71	0,71	0,66	0,82	0,68	0,68	0,68	0,89	0,82	0,66	0,85	0,85	0,66	0,77	0,68	0,72	0,69	0,65	0,65	0,64	0,87	0,68	0,82	0,66	0,82	0,82
NC_034560.1	0,77	0,68	0,72	0,67	0,79	0,71	0,70	0,66	0,79	0,80	0,71	0,77	0,77	0,69	0,85	0,67	0,71	0,68	0,67	0,67	0,66	0,78	0,67	0,80	0,71	0,80	0,79
NC_034564.1	0,62	0,68	0,65	0,63	0,66	0,66	0,66	0,66	0,68	0,65	0,65	0,67	0,67	0,65	0,60	0,61	0,74	0,75	0,68	0,68	0,68	0,66	0,61	0,66	0,64	0,66	0,66
NC_038529.1	0,65	0,75	0,84	0,76	0,68	0,78	0,77	0,72	0,67	0,68	0,81	0,68	0,68	0,81	0,65	0,73	0,66	0,61	0,62	0,62	0,63	0,67	0,73	0,67	0,80	0,67	0,68
NC_043068.1	0,76	0,68	0,71	0,66	0,77	0,72	0,71	0,66	0,80	0,77	0,72	0,75	0,75	0,71	0,83	0,66	0,72	0,69	0,66	0,66	0,65	0,79	0,66	0,77	0,72	0,77	0,77
NC_043175.1	0,72	0,67	0,69	0,66	0,77	0,70	0,69	0,63	0,75	0,77	0,66	0,72	0,72	0,65	0,77	0,66	0,70	0,65	0,66	0,66	0,66	0,74	0,66	0,77	0,66	0,77	0,77
NC_043407.1	0,65	0,86	0,75	0,86	0,66	0,80	0,80	0,70	0,68	0,66	0,76	0,70	0,70	0,76	0,67	0,84	0,64	0,66	0,62	0,62	0,62	0,69	0,84	0,66	0,75	0,66	0,66
NC_055147.1	0,72	0,67	0,69	0,62	0,75	0,66	0,66	0,67	0,80	0,75	0,70	0,75	0,75	0,68	0,77	0,64	0,69	0,66	0,64	0,64	0,63	0,79	0,64	0,75	0,70	0,75	0,75
NC_055632.1	0,66	0,66	0,66	0,63	0,68	0,68	0,69	0,68	0,68	0,68	0,65	0,67	0,67	0,65	0,67	0,66	0,76	0,70	0,70	0,70	0,69	0,68	0,66	0,68	0,66	0,68	0,68
NC_055636.1	0,67	0,83	0,91	0,81	0,71	0,87	0,84	0,79	0,70	0,71	0,87	0,71	0,71	0,89	0,68	0,81	0,71	0,66	0,65	0,65	0,64	0,69	0,81	0,71	0,87	0,71	0,71
NC_078485.1	0,62	0,62	0,66	0,61	0,64	0,65	0,65	0,62	0,62	0,64	0,62	0,62	0,62	0,66	0,62	0,62	0,67	0,70	1,00	1,00	0,98	0,62	0,62	0,64	0,63	0,64	0,64

	KX845680.1	KY594712.1	KY662269.1	KY662275.1	MG717393.1	KY978757.1	MN183135.1	MN850093.1	MN850096.1	MN639737.1	MN639740.1	MT024592.1	MT514291.1	MT514292.1	MT514294.1	MT514295.1	MT514296.1	MT514297.1	MT514298.1	MN258157.1	MN832779.1	MN832782.1	MT648514.1	OM030302.1	OM030305.1	OM030311.1	OK422869.1
Mamanguape_virus_EM_725	0,78	0,66	0,67	0,70	0,64	0,67	0,66	0,67	0,67	0,63	0,63	0,64	0,63	0,64	0,64	0,66	0,66	0,67	0,66	0,67	0,64	0,64	0,69	0,78	0,69	0,64	0,71
Mamanguape_virus_EM_711	0,78	0,67	0,71	0,74	0,66	0,67	0,66	0,66	0,66	0,66	0,66	0,67	0,66	0,67	0,67	0,66	0,66	0,67	0,66	0,66	0,67	0,67	0,70	0,78	0,70	0,66	0,70
Mamanguape_virus_EM_708	0,73	0,64	0,67	0,69	0,62	0,64	0,63	0,63	0,63	0,63	0,63	0,64	0,63	0,64	0,64	0,62	0,63	0,64	0,62	0,63	0,64	0,64	0,67	0,73	0,66	0,64	0,68
OR684449.1	0,79	0,67	0,70	0,73	0,65	0,67	0,67	0,66	0,66	0,66	0,66	0,66	0,66	0,66	0,66	0,66	0,67	0,68	0,66	0,66	0,66	0,66	0,69	0,77	0,70	0,66	0,70
MG663536.1	0,75	0,68	0,77	0,78	0,64	0,67	0,66	0,66	0,66	0,68	0,68	0,66	0,68	0,67	0,66	0,65	0,66	0,67	0,65	0,66	0,65	0,66	0,65	0,73	0,68	0,68	0,69
JN037851.1	0,76	0,63	0,79	0,75	0,64	0,61	0,64	0,67	0,67	0,63	0,63	0,64	0,64	0,63	0,64	0,66	0,64	0,66	0,66	0,67	0,64	0,64	0,69	0,73	0,62	0,64	0,64
KT316176.1	0,72	0,67	0,78	0,79	0,62	0,65	0,66	0,65	0,65	0,62	0,62	0,62	0,62	0,62	0,62	0,65	0,66	0,66	0,65	0,65	0,62	0,62	0,66	0,71	0,65	0,65	0,66
JQ287716.2	0,79	0,69	0,76	0,77	0,67	0,67	0,68	0,70	0,70	0,66	0,66	0,67	0,66	0,65	0,67	0,68	0,68	0,69	0,68	0,70	0,66	0,67	0,69	0,79	0,71	0,66	0,72
OP122967.1	0,76	0,68	0,74	0,77	0,70	0,67	0,68	0,68	0,68	0,66	0,66	0,66	0,66	0,66	0,66	0,67	0,68	0,70	0,67	0,68	0,66	0,66	0,65	0,72	0,66	0,69	0,70
JX465369.1	0,80	0,66	0,70	0,71	0,64	0,66	0,62	0,66	0,66	0,62	0,62	0,63	0,61	0,62	0,63	0,61	0,62	0,63	0,61	0,66	0,64	0,63	0,68	0,76	0,67	0,64	0,67
Mamanguape_virus_EM_709	0,78	0,67	0,70	0,73	0,64	0,67	0,67	0,66	0,66	0,65	0,65	0,66	0,65	0,66	0,66	0,66	0,67	0,68	0,66	0,66	0,66	0,66	0,69	0,77	0,70	0,65	0,70
Mamanguape_virus_EM_707	0,79	0,67	0,69	0,72	0,65	0,67	0,67	0,66	0,66	0,66	0,66	0,66	0,66	0,66	0,66	0,66	0,67	0,68	0,66	0,66	0,66	0,66	0,69	0,77	0,70	0,66	0,70
Hantavirus_HantaV1_KX442773_1	0,77	0,66	0,70	0,73	0,64	0,66	0,65	0,65	0,65	0,65	0,65	0,66	0,65	0,66	0,66	0,64	0,65	0,66	0,64	0,65	0,66	0,66	0,69	0,77	0,69	0,65	0,69
Hantavirus_HantaV1_KX442793_1	0,79	0,65	0,70	0,73	0,64	0,65	0,65	0,64	0,64	0,64	0,64	0,64	0,64	0,64	0,64	0,64	0,65	0,66	0,64	0,64	0,64	0,64	0,69	0,77	0,68	0,65	0,70
Hantavirus_HantaV1_KX442794_1	0,79	0,65	0,70	0,73	0,64	0,65	0,65	0,64	0,64	0,64	0,64	0,64	0,64	0,64	0,64	0,64	0,65	0,66	0,64	0,64	0,64	0,64	0,69	0,77	0,68	0,65	0,70
Hantavirus_HantaV2_KX442772_1	0,78	0,67	0,70	0,73	0,64	0,65	0,66	0,65	0,65	0,64	0,64	0,65	0,64	0,65	0,65	0,65	0,66	0,67	0,65	0,65	0,65	0,65	0,68	0,75	0,70	0,64	0,68
Hantavirus_HantaV2_KX442771_1	0,77	0,67	0,69	0,72	0,64	0,65	0,65	0,64	0,64	0,64	0,64	0,64	0,64	0,64	0,64	0,64	0,65	0,66	0,64	0,64	0,64	0,64	0,66	0,74	0,69	0,64	0,67
Hantavirus_HantaV3_KX442770_1	0,76	0,69	0,69	0,68	0,67	0,68	0,64	0,69	0,69	0,64	0,64	0,64	0,64	0,66	0,64	0,64	0,64	0,65	0,64	0,69	0,64	0,64	0,69	0,77	0,67	0,66	0,69
EF405801.1	0,66	0,67	0,69	0,69	0,80	0,66	0,87	0,81	0,82	0,98	0,98	0,99	0,98	0,94	0,99	0,86	0,87	0,86	0,86	0,81	0,97	0,99	0,60	0,68	0,67	0,68	0,66
NC_005217.1	0,66	0,67	0,69	0,67	0,95	0,66	0,80	0,86	0,86	0,80	0,80	0,78	0,81	0,79	0,78	0,80	0,80	0,81	0,80	0,86	0,77	0,78	0,60	0,67	0,66	0,67	0,67
NC_005226.1	0,66	0,67	0,68	0,67	0,78	0,66	0,97	0,82	0,82	0,83	0,83	0,85	0,84	0,84	0,85	1,00	0,98	0,96	1,00	0,82	0,85	0,85	0,61	0,68	0,66	0,66	0,67
NC_006435.1	0,68	1,00	0,64	0,65	0,70	0,98	0,69	0,68	0,68	0,66	0,66	0,67	0,66	0,66	0,67	0,67	0,69	0,69	0,67	0,68	0,67	0,67	0,61	0,68	0,94	0,79	0,80
NC_055170.1	0,63	0,66	0,62	0,62	0,65	0,65	0,66	0,66	0,66	0,63	0,63	0,64	0,62	0,63	0,64	0,66	0,66	0,66	0,66	0,66	0,64	0,64	0,59	0,65	0,63	0,65	0,62
AF291704.5	0,68	0,68	0,68	0,68	0,87	0,68	0,82	0,99	0,98	0,79	0,79	0,80	0,80	0,78	0,80	0,81	0,82	0,82	0,81	0,99	0,78	0,80	0,61	0,67	0,66	0,67	0,66
AY526217.1	0,65	0,66	0,66	0,66	0,77	0,65	0,86	0,77	0,78	0,95	0,95	0,97	0,94	0,93	0,97	0,84	0,86	0,84	0,84	0,77	0,99	0,97	0,58	0,66	0,66	0,68	0,66
DQ371906.1	0,68	0,96	0,66	0,66	0,70	0,93	0,70	0,66	0,66	0,66	0,66	0,68	0,66	0,66	0,68	0,69	0,70	0,68	0,69	0,66	0,68	0,68	0,61	0,69	0,96	0,78	0,80
DQ825770.1	0,82	0,67	0,74	0,73	0,66	0,65	0,62	0,66	0,66	0,64	0,64	0,65	0,63	0,64	0,65	0,63	0,62	0,63	0,63	0,66	0,65	0,65	0,69	0,78	0,66	0,62	0,66
DQ989237.1	0,67	0,92	0,63	0,62	0,68	0,92	0,66	0,66	0,66	0,64	0,64	0,66	0,64	0,65	0,66	0,66	0,66	0,66	0,66	0,66	0,66	0,66	0,61	0,67	0,98	0,75	0,80
EF397003.1	0,70	0,66	0,67	0,66	0,87	0,66	0,79	0,88	0,88	0,77	0,77	0,77	0,77	0,76	0,77	0,80	0,79	0,81	0,80	0,88	0,77	0,77	0,61	0,71	0,66	0,68	0,67
EF646763.1	0,67	0,70	0,68	0,69	0,79	0,68	0,88	0,80	0,80	0,85	0,85	0,87	0,85	0,86	0,87	0,89	0,88	0,90	0,89	0,80	0,87	0,87	0,62	0,69	0,69	0,69	0,69
EU788002.1	0,67	0,64	0,67	0,66	0,85	0,63	0,79	0,89	0,88	0,80	0,80	0,78	0,79	0,80	0,78	0,81	0,79	0,82	0,81	0,89	0,78	0,78	0,61	0,66	0,62	0,66	0,64
GU140096.1	0,68	0,99	0,65	0,65	0,69	0,97	0,69	0,67	0,67	0,67	0,67	0,68	0,67	0,67	0,68	0,67	0,69	0,68	0,67	0,67	0,68	0,68	0,61	0,68	0,95	0,78	0,81
FJ858378.1	0,72	0,69	0,71	0,70	0,85	0,68	0,81	0,87	0,87	0,79	0,79	0,81	0,80	0,79	0,81	0,81	0,81	0,82	0,81	0,87	0,81	0,81	0,63	0,71	0,70	0,68	0,68
GQ244526.1	0,72	0,70	0,70	0,68	0,84	0,69	0,79	0,87	0,87	0,78	0,78	0,79	0,79	0,78	0,79	0,78	0,79	0,79	0,78	0,87	0,79	0,79	0,62	0,71	0,69	0,67	0,66
HM015220.1	0,66	0,64	0,65	0,66	0,72	0,63	0,76	0,73	0,73	0,74	0,74	0,76	0,75	0,72	0,76	0,75	0,76	0,77	0,75	0,73	0,75	0,76	0,58	0,68	0,63	0,63	0,69
JQ026206.1	0,69	0,81	0,68	0,67	0,70	0,80	0,69	0,66	0,66	0,66	0,66	0,68	0,66	0,67	0,68	0,70	0,69	0,68	0,70	0,66	0,68	0,68	0,61	0,71	0,82	0,78	0,97
JQ083393.1	0,68	1,00	0,64	0,65	0,70	0,98	0,69	0,68	0,68	0,66	0,66	0,67	0,66	0,66	0,67	0,67	0,69	0,69	0,67	0,68	0,67	0,67	0,61	0,68	0,94	0,79	0,80
JN831945.1	0,65	0,67	0,68	0,68	0,79	0,66	0,87	0,80	0,81	0,98	0,98	1,00	0,98	0,95	1,00	0,85	0,87	0,85	0,85	0,80	0,98	1,00	0,59	0,67	0,67	0,68	0,66
JX853574.1	0,66	0,82	0,70	0,69	0,71	0,81	0,70	0,68	0,68	0,66	0,66	0,67	0,66	0,66	0,67	0,71	0,70	0,70	0,71	0,68	0,67	0,67	0,62	0,71	0,82	0,75	0,86
JX879770.1	0,66	0,82	0,70	0,69	0,71	0,81	0,70	0,68	0,68	0,66	0,66	0,67	0,66	0,66	0,67	0,71	0,70	0,70	0,71	0,68	0,67	0,67	0,62	0,71	0,82	0,75	0,86
JX028271.1	0,67	0,67	0,68	0,68	0,79	0,66	0,88	0,79	0,79	0,92	0,92	0,94	0,93	0,91	0,94	0,87	0,88	0,88	0,87	0,79	0,94	0,94	0,61	0,70	0,66	0,66	0,68

KF705679.1	0,66	0,76	0,64	0,64	0,66	0,75	0,66	0,66	0,66	0,66	0,66	0,66	0,66	0,66	0,66	0,65	0,66	0,65	0,65	0,66	0,66	0,66	0,63	0,66	0,75	0,81	0,76
KF537001.1	0,66	0,67	0,69	0,67	0,95	0,66	0,80	0,86	0,86	0,80	0,80	0,78	0,81	0,79	0,78	0,80	0,80	0,81	0,80	0,86	0,77	0,78	0,60	0,67	0,66	0,67	0,67
KM102249.1	0,74	0,68	0,81	0,81	0,66	0,66	0,68	0,66	0,66	0,66	0,66	0,68	0,67	0,67	0,68	0,68	0,68	0,70	0,68	0,66	0,68	0,68	0,65	0,74	0,69	0,65	0,69
KR072623.1	0,79	0,63	0,67	0,67	0,64	0,61	0,63	0,63	0,63	0,61	0,61	0,63	0,61	0,62	0,63	0,65	0,63	0,64	0,65	0,63	0,64	0,63	0,71	0,74	0,66	0,64	0,68
KM361055.1	0,74	0,64	0,65	0,67	0,63	0,63	0,64	0,61	0,61	0,62	0,62	0,62	0,63	0,62	0,62	0,62	0,64	0,64	0,62	0,61	0,63	0,62	0,87	0,67	0,64	0,66	0,61
KM361056.1	0,74	0,64	0,65	0,67	0,63	0,63	0,64	0,61	0,61	0,62	0,62	0,62	0,63	0,62	0,62	0,62	0,64	0,64	0,62	0,61	0,63	0,62	0,87	0,67	0,64	0,66	0,61
KM361061.1	0,72	0,63	0,65	0,67	0,62	0,62	0,63	0,60	0,60	0,63	0,63	0,63	0,62	0,63	0,63	0,61	0,63	0,63	0,61	0,60	0,64	0,63	0,86	0,66	0,63	0,65	0,61
KT885041.1	0,68	0,81	0,67	0,67	0,71	0,82	0,69	0,67	0,67	0,66	0,66	0,67	0,66	0,66	0,67	0,68	0,69	0,68	0,68	0,67	0,67	0,67	0,61	0,71	0,82	0,77	0,98
KT885044.1	0,66	0,67	0,69	0,67	0,95	0,66	0,80	0,86	0,86	0,80	0,80	0,78	0,81	0,79	0,78	0,80	0,80	0,81	0,80	0,86	0,77	0,78	0,60	0,67	0,66	0,67	0,67
KT885047.1	0,68	0,99	0,64	0,64	0,70	0,97	0,68	0,68	0,68	0,66	0,66	0,67	0,66	0,66	0,67	0,67	0,68	0,68	0,67	0,68	0,67	0,67	0,61	0,68	0,94	0,79	0,79
KT885050.1	0,66	0,67	0,69	0,69	0,80	0,66	0,87	0,81	0,82	0,98	0,98	0,99	0,98	0,94	0,99	0,86	0,87	0,86	0,86	0,81	0,97	0,99	0,60	0,68	0,67	0,68	0,66
KT934965.1	0,68	0,99	0,64	0,64	0,70	0,97	0,68	0,68	0,68	0,66	0,66	0,67	0,66	0,66	0,67	0,67	0,68	0,68	0,67	0,68	0,67	0,67	0,61	0,68	0,94	0,79	0,79
KU215675.1	0,68	0,99	0,65	0,65	0,69	0,97	0,69	0,67	0,67	0,67	0,67	0,68	0,67	0,67	0,68	0,67	0,69	0,68	0,67	0,67	0,68	0,68	0,61	0,68	0,95	0,78	0,81
KX845680.1	1,00	0,68	0,69	0,71	0,68	0,66	0,66	0,68	0,68	0,66	0,66	0,65	0,66	0,66	0,65	0,66	0,66	0,67	0,66	0,68	0,66	0,65	0,73	0,81	0,68	0,67	0,67
KY594712.1	0,68	1,00	0,64	0,65	0,70	0,98	0,69	0,68	0,68	0,66	0,66	0,67	0,66	0,66	0,67	0,67	0,69	0,69	0,67	0,68	0,67	0,67	0,61	0,68	0,94	0,79	0,80
KY662269.1	0,69	0,64	1,00	0,95	0,69	0,62	0,67	0,68	0,68	0,66	0,66	0,68	0,67	0,66	0,68	0,68	0,67	0,68	0,68	0,68	0,67	0,68	0,66	0,74	0,65	0,66	0,66
KY662275.1	0,71	0,65	0,95	1,00	0,69	0,63	0,69	0,68	0,68	0,66	0,66	0,68	0,67	0,66	0,68	0,67	0,69	0,70	0,67	0,68	0,67	0,68	0,65	0,72	0,63	0,67	0,66
MG717393.1	0,68	0,70	0,69	0,69	1,00	0,69	0,80	0,87	0,87	0,79	0,79	0,79	0,80	0,78	0,79	0,78	0,80	0,81	0,78	0,87	0,78	0,79	0,61	0,69	0,69	0,69	0,70
KY978757.1	0,66	0,98	0,62	0,63	0,69	1,00	0,67	0,68	0,68	0,65	0,65	0,66	0,65	0,65	0,66	0,66	0,67	0,67	0,66	0,68	0,66	0,66	0,60	0,68	0,93	0,77	0,81
MN183135.1	0,66	0,69	0,67	0,69	0,80	0,67	1,00	0,82	0,82	0,85	0,85	0,87	0,86	0,85	0,87	0,97	0,99	0,96	0,97	0,82	0,87	0,87	0,59	0,66	0,67	0,67	0,68
MN850093.1	0,68	0,68	0,68	0,68	0,87	0,68	0,82	1,00	0,99	0,79	0,79	0,80	0,80	0,78	0,80	0,82	0,82	0,83	0,82	1,00	0,78	0,80	0,61	0,67	0,66	0,67	0,66
MN850096.1	0,68	0,68	0,68	0,68	0,87	0,68	0,82	0,99	1,00	0,80	0,80	0,81	0,81	0,79	0,81	0,82	0,82	0,83	0,82	0,99	0,79	0,81	0,61	0,67	0,66	0,67	0,66
MN639737.1	0,66	0,66	0,66	0,66	0,79	0,65	0,85	0,79	0,80	1,00	1,00	0,98	0,99	0,97	0,98	0,83	0,85	0,83	0,83	0,79	0,96	0,98	0,59	0,66	0,66	0,68	0,65
MN639740.1	0,66	0,66	0,66	0,66	0,79	0,65	0,85	0,79	0,80	1,00	1,00	0,98	0,99	0,97	0,98	0,83	0,85	0,83	0,83	0,79	0,96	0,98	0,59	0,66	0,66	0,68	0,65
MT024592.1	0,65	0,67	0,68	0,68	0,79	0,66	0,87	0,80	0,81	0,98	0,98	1,00	0,98	0,95	1,00	0,85	0,87	0,85	0,85	0,80	0,98	1,00	0,59	0,67	0,67	0,68	0,66
MT514291.1	0,66	0,66	0,67	0,67	0,80	0,65	0,86	0,80	0,81	0,99	0,99	0,98	1,00	0,96	0,98	0,84	0,86	0,84	0,84	0,80	0,95	0,98	0,60	0,66	0,66	0,68	0,65
MT514292.1	0,66	0,66	0,66	0,66	0,78	0,65	0,85	0,78	0,79	0,97	0,97	0,95	0,96	1,00	0,95	0,84	0,85	0,83	0,84	0,78	0,94	0,95	0,59	0,66	0,66	0,68	0,65
MT514294.1	0,65	0,67	0,68	0,68	0,79	0,66	0,87	0,80	0,81	0,98	0,98	1,00	0,98	0,95	1,00	0,85	0,87	0,85	0,85	0,80	0,98	1,00	0,59	0,67	0,67	0,68	0,66
MT514295.1	0,66	0,67	0,68	0,67	0,78	0,66	0,97	0,82	0,82	0,83	0,83	0,85	0,84	0,84	0,85	1,00	0,98	0,96	1,00	0,82	0,85	0,85	0,61	0,68	0,66	0,66	0,67
MT514296.1	0,66	0,69	0,67	0,69	0,80	0,67	0,99	0,82	0,82	0,85	0,85	0,87	0,86	0,85	0,87	0,98	1,00	0,97	0,98	0,82	0,87	0,87	0,59	0,66	0,67	0,67	0,68
MT514297.1	0,67	0,69	0,68	0,70	0,81	0,67	0,96	0,83	0,83	0,83	0,83	0,85	0,84	0,83	0,85	0,96	0,97	1,00	0,96	0,83	0,85	0,85	0,61	0,67	0,66	0,66	0,67
MT514298.1	0,66	0,67	0,68	0,67	0,78	0,66	0,97	0,82	0,82	0,83	0,83	0,85	0,84	0,84	0,85	1,00	0,98	0,96	1,00	0,82	0,85	0,85	0,61	0,68	0,66	0,66	0,67
MN258157.1	0,68	0,68	0,68	0,68	0,87	0,68	0,82	1,00	0,99	0,79	0,79	0,80	0,80	0,78	0,80	0,82	0,82	0,83	0,82	1,00	0,78	0,80	0,61	0,67	0,66	0,67	0,66
MN832779.1	0,66	0,67	0,67	0,67	0,78	0,66	0,87	0,78	0,79	0,96	0,96	0,98	0,95	0,94	0,98	0,85	0,87	0,85	0,85	0,78	1,00	0,98	0,59	0,67	0,67	0,69	0,66
MN832782.1	0,65	0,67	0,68	0,68	0,79	0,66	0,87	0,80	0,81	0,98	0,98	1,00	0,98	0,95	1,00	0,85	0,87	0,85	0,85	0,80	0,98	1,00	0,59	0,67	0,67	0,68	0,66
MT648514.1	0,73	0,61	0,66	0,65	0,61	0,60	0,59	0,61	0,61	0,59	0,59	0,59	0,60	0,59	0,59	0,61	0,59	0,61	0,61	0,61	0,59	0,59	1,00	0,69	0,62	0,63	0,61
OM030302.1	0,81	0,68	0,74	0,72	0,69	0,68	0,66	0,67	0,67	0,66	0,66	0,67	0,66	0,66	0,67	0,68	0,66	0,67	0,68	0,67	0,67	0,67	0,69	1,00	0,68	0,65	0,70
OM030305.1	0,68	0,94	0,65	0,63	0,69	0,93	0,67	0,66	0,66	0,66	0,66	0,67	0,66	0,66	0,67	0,66	0,67	0,66	0,66	0,66	0,67	0,67	0,62	0,68	1,00	0,77	0,82
OM030311.1	0,67	0,79	0,66	0,67	0,69	0,77	0,67	0,67	0,67	0,68	0,68	0,68	0,68	0,68	0,68	0,66	0,67	0,66	0,66	0,67	0,69	0,68	0,63	0,65	0,77	1,00	0,78
OK422869.1	0,67	0,80	0,66	0,66	0,70	0,81	0,68	0,66	0,66	0,65	0,65	0,66	0,65	0,65	0,66	0,67	0,68	0,67	0,67	0,66	0,66	0,66	0,61	0,70	0,82	0,78	1,00
MZ504241.1	0,68	0,82	0,69	0,70	0,71	0,81	0,69	0,67	0,67	0,65	0,65	0,66	0,65	0,66	0,66	0,70	0,69	0,69	0,70	0,67	0,66	0,66	0,61	0,70	0,82	0,75	0,86
OQ092243.1	0,65	0,66	0,66	0,66	0,85	0,66	0,80	0,93	0,93	0,78	0,78	0,79	0,77	0,77	0,79	0,78	0,80	0,81	0,78	0,93	0,77	0,79	0,59	0,66	0,64	0,66	0,65
OR148904.1	0,69	0,69	0,69	0,67	0,87	0,69	0,82	0,87	0,86	0,81	0,81	0,80	0,82	0,81	0,80	0,82	0,82	0,82	0,82	0,87	0,79	0,80	0,62	0,71	0,68	0,67	0,70
OR365538.1	0,56	0,55	0,54	0,53	0,71	0,55	0,64	0,71	0,71	0,61	0,61	0,62	0,62	0,61	0,62	0,64	0,64	0,65	0,64	0,71	0,62	0,62	0,48	0,57	0,54	0,54	0,55
NC_005235.1	0,68	0,81	0,67	0,67	0,71	0,82	0,69	0,67	0,67	0,66	0,66	0,67	0,66	0,66	0,67	0,68	0,69	0,68	0,68	0,67	0,67	0,67	0,61	0,71	0,82	0,77	0,99
NC_034401.1	0,77	0,69	0,75	0,77	0,65	0,68	0,68	0,66	0,66	0,67	0,67	0,68	0,68	0,66	0,68	0,66	0,68	0,69	0,66	0,66	0,67	0,68	0,67	0,75	0,66	0,66	0,68
NC_034399.1	0,68	0,77	0,66	0,68	0,66	0,76	0,69	0,69	0,69	0,70	0,70	0,70	0,70	0,69	0,70	0,68	0,69	0,68	0,68	0,69	0,69	0,70	0,61	0,66	0,75	0,79	0,74

NC_034402.1	0,66	0,70	0,64	0,65	0,69	0,70	0,67	0,69	0,69	0,66	0,66	0,66	0,65	0,66	0,66	0,68	0,67	0,67	0,68	0,69	0,66	0,66	0,61	0,67	0,71	0,69	0,71
NC_034403.1	0,66	0,71	0,69	0,69	0,87	0,71	0,82	0,86	0,86	0,82	0,82	0,83	0,81	0,81	0,83	0,82	0,82	0,82	0,82	0,86	0,82	0,83	0,61	0,69	0,72	0,70	0,71
NC_034407.1	0,71	0,72	0,70	0,69	0,68	0,71	0,71	0,68	0,68	0,72	0,72	0,72	0,72	0,72	0,72	0,70	0,71	0,70	0,70	0,68	0,73	0,72	0,62	0,69	0,71	0,78	0,71
NC_034467.1	0,70	0,73	0,67	0,69	0,82	0,73	0,82	0,81	0,81	0,79	0,79	0,80	0,80	0,79	0,80	0,80	0,82	0,82	0,80	0,81	0,82	0,80	0,62	0,71	0,72	0,71	0,72
NC_034485.1	0,67	0,81	0,67	0,66	0,70	0,81	0,69	0,70	0,70	0,68	0,68	0,69	0,68	0,68	0,69	0,69	0,69	0,67	0,69	0,70	0,69	0,69	0,68	0,70	0,81	0,87	0,81
NC_034515.1	0,70	0,66	0,68	0,67	0,87	0,65	0,82	0,87	0,87	0,80	0,80	0,80	0,81	0,80	0,80	0,81	0,82	0,82	0,81	0,87	0,80	0,80	0,60	0,69	0,66	0,66	0,67
NC_034517.1	0,71	0,84	0,68	0,70	0,73	0,82	0,69	0,69	0,69	0,68	0,68	0,68	0,68	0,68	0,68	0,69	0,69	0,69	0,69	0,69	0,68	0,68	0,62	0,71	0,82	0,79	0,84
NC_034519.1	0,69	0,70	0,67	0,68	0,83	0,68	0,89	0,83	0,84	0,90	0,90	0,92	0,91	0,90	0,92	0,88	0,89	0,90	0,88	0,83	0,92	0,92	0,61	0,71	0,68	0,70	0,68
NC_034556.1	0,71	0,82	0,67	0,66	0,69	0,82	0,69	0,69	0,69	0,66	0,66	0,66	0,66	0,66	0,66	0,71	0,69	0,69	0,71	0,69	0,66	0,66	0,64	0,71	0,80	0,80	0,88
NC_034560.1	0,71	0,80	0,71	0,72	0,70	0,78	0,70	0,71	0,71	0,71	0,71	0,71	0,71	0,70	0,71	0,69	0,70	0,70	0,69	0,71	0,70	0,71	0,66	0,70	0,78	0,83	0,77
NC_034564.1	0,78	0,65	0,68	0,69	0,63	0,63	0,64	0,66	0,66	0,64	0,64	0,65	0,63	0,64	0,65	0,64	0,65	0,66	0,64	0,66	0,65	0,65	0,65	0,74	0,63	0,62	0,66
NC_038529.1	0,64	0,68	0,67	0,69	0,74	0,66	0,81	0,77	0,77	0,80	0,80	0,81	0,79	0,79	0,81	0,79	0,81	0,81	0,79	0,77	0,79	0,81	0,59	0,65	0,66	0,66	0,66
NC_043068.1	0,68	0,77	0,72	0,72	0,67	0,75	0,69	0,68	0,68	0,71	0,71	0,72	0,71	0,70	0,72	0,69	0,69	0,68	0,69	0,68	0,71	0,72	0,65	0,69	0,77	0,84	0,80
NC_043175.1	0,67	0,77	0,69	0,71	0,70	0,75	0,66	0,67	0,67	0,66	0,66	0,66	0,66	0,66	0,66	0,66	0,66	0,66	0,66	0,67	0,67	0,66	0,61	0,69	0,74	0,79	0,73
NC_043407.1	0,70	0,66	0,69	0,67	0,83	0,66	0,79	0,87	0,86	0,77	0,77	0,76	0,76	0,78	0,76	0,80	0,80	0,79	0,80	0,87	0,76	0,76	0,61	0,68	0,66	0,71	0,68
NC_055147.1	0,69	0,75	0,65	0,66	0,67	0,73	0,68	0,66	0,66	0,69	0,69	0,70	0,69	0,67	0,70	0,66	0,68	0,66	0,66	0,66	0,68	0,70	0,61	0,68	0,74	0,78	0,79
NC_055632.1	0,74	0,68	0,73	0,75	0,67	0,67	0,65	0,66	0,66	0,65	0,65	0,65	0,66	0,64	0,65	0,64	0,65	0,66	0,64	0,66	0,64	0,65	0,69	0,72	0,67	0,71	0,69
NC_055636.1	0,69	0,71	0,67	0,68	0,83	0,69	0,89	0,85	0,85	0,85	0,85	0,87	0,86	0,85	0,87	0,89	0,89	0,89	0,89	0,85	0,87	0,87	0,63	0,71	0,69	0,68	0,68
NC_078485.1	0,74	0,64	0,65	0,67	0,63	0,63	0,64	0,61	0,61	0,62	0,62	0,62	0,63	0,62	0,62	0,62	0,64	0,64	0,62	0,61	0,63	0,62	0,87	0,67	0,64	0,66	0,61

	MZ504241.1	OQ092243.1	OR148904.1	OR365538.1	NC_005235.1	NC_034401.1	NC_034399.1	NC_034402.1	NC_034403.1	NC_034407.1	NC_034467.1	NC_034485.1	NC_034515.1	NC_034517.1	NC_034519.1	NC_034556.1	NC_034560.1	NC_034564.1	NC_038529.1	NC_043068.1	NC_043175.1	NC_043407.1	NC_055147.1	NC_055632.1	NC_055636.1	NC_078485.1
Mamanguape_virus_EM_725	0,71	0,64	0,69	0,50	0,72	0,76	0,68	0,66	0,66	0,64	0,68	0,68	0,67	0,71	0,67	0,70	0,70	0,73	0,62	0,69	0,63	0,66	0,64	0,72	0,68	0,66
Mamanguape_virus_EM_711	0,71	0,64	0,68	0,52	0,71	0,78	0,70	0,66	0,66	0,68	0,70	0,68	0,67	0,71	0,68	0,69	0,71	0,74	0,66	0,69	0,66	0,65	0,66	0,74	0,69	0,69
Mamanguape_virus_EM_708	0,67	0,60	0,64	0,52	0,67	0,72	0,68	0,62	0,63	0,64	0,66	0,66	0,64	0,68	0,64	0,67	0,67	0,70	0,63	0,67	0,62	0,62	0,64	0,71	0,65	0,66
OR684449.1	0,71	0,64	0,67	0,52	0,71	0,78	0,71	0,66	0,66	0,67	0,69	0,67	0,66	0,71	0,67	0,69	0,70	0,74	0,66	0,68	0,65	0,65	0,66	0,73	0,68	0,69
MG663536.1	0,70	0,65	0,67	0,52	0,70	0,79	0,68	0,67	0,65	0,70	0,65	0,69	0,65	0,70	0,66	0,72	0,71	0,76	0,64	0,70	0,66	0,66	0,70	0,78	0,67	0,68
JN037851.1	0,65	0,64	0,66	0,54	0,63	0,71	0,66	0,66	0,61	0,67	0,63	0,66	0,65	0,66	0,64	0,68	0,66	0,72	0,62	0,68	0,69	0,66	0,66	0,70	0,66	0,64
KT316176.1	0,68	0,62	0,63	0,51	0,65	0,75	0,69	0,68	0,61	0,70	0,65	0,68	0,62	0,66	0,65	0,73	0,71	0,71	0,64	0,72	0,68	0,62	0,69	0,75	0,67	0,68
JQ287716.2	0,74	0,68	0,68	0,54	0,73	0,78	0,71	0,69	0,66	0,67	0,69	0,67	0,68	0,72	0,70	0,71	0,70	0,78	0,66	0,71	0,69	0,66	0,68	0,75	0,70	0,73
OP122967.1	0,72	0,66	0,68	0,58	0,71	0,78	0,74	0,66	0,64	0,70	0,70	0,69	0,68	0,72	0,69	0,71	0,74	0,69	0,67	0,71	0,70	0,66	0,67	0,77	0,69	0,65
JX465369.1	0,70	0,66	0,62	0,55	0,68	0,75	0,66	0,70	0,61	0,67	0,64	0,65	0,62	0,68	0,64	0,74	0,66	0,77	0,62	0,66	0,67	0,65	0,64	0,73	0,66	0,70
Mamanguape_virus_EM_709	0,70	0,64	0,67	0,52	0,70	0,78	0,70	0,65	0,65	0,67	0,69	0,67	0,66	0,70	0,67	0,69	0,70	0,74	0,65	0,68	0,64	0,65	0,65	0,73	0,68	0,68
Mamanguape_virus_EM_707	0,71	0,64	0,67	0,52	0,71	0,78	0,71	0,66	0,66	0,67	0,69	0,67	0,66	0,71	0,67	0,69	0,70	0,74	0,66	0,68	0,65	0,65	0,66	0,72	0,68	0,69
Hantavirus_HantaV1_KX442773_1	0,70	0,63	0,67	0,50	0,70	0,77	0,69	0,64	0,65	0,67	0,69	0,67	0,66	0,70	0,67	0,68	0,70	0,74	0,65	0,68	0,65	0,64	0,64	0,73	0,68	0,68
Hantavirus_HantaV1_KX442793_1	0,70	0,62	0,65	0,51	0,70	0,78	0,69	0,65	0,64	0,67	0,67	0,66	0,64	0,70	0,65	0,69	0,69	0,74	0,64	0,67	0,65	0,65	0,65	0,73	0,66	0,68
Hantavirus_HantaV1_KX442794_1	0,70	0,62	0,65	0,51	0,70	0,78	0,69	0,65	0,64	0,67	0,67	0,66	0,64	0,70	0,65	0,69	0,69	0,74	0,64	0,67	0,65	0,65	0,65	0,73	0,66	0,68
Hantavirus_HantaV2_KX442772_1	0,70	0,63	0,66	0,50	0,69	0,78	0,70	0,64	0,64	0,66	0,68	0,66	0,65	0,70	0,66	0,67	0,69	0,73	0,64	0,67	0,65	0,64	0,64	0,72	0,67	0,68
Hantavirus_HantaV2_KX442771_1	0,70	0,62	0,65	0,50	0,68	0,78	0,69	0,64	0,64	0,65	0,67	0,65	0,64	0,70	0,65	0,66	0,68	0,72	0,64	0,66	0,64	0,64	0,64	0,71	0,67	0,67
Hantavirus_HantaV3_KX442770_1	0,71	0,66	0,69	0,56	0,70	0,77	0,68	0,67	0,67	0,68	0,69	0,69	0,67	0,71	0,69	0,72	0,70	0,74	0,64	0,69	0,66	0,68	0,66	0,74	0,69	0,68
EF405801.1	0,66	0,78	0,81	0,63	0,67	0,69	0,70	0,66	0,82	0,72	0,81	0,69	0,81	0,68	0,92	0,66	0,71	0,64	0,80	0,72	0,66	0,75	0,70	0,66	0,87	0,63
NC_005217.1	0,71	0,86	0,89	0,70	0,68	0,64	0,66	0,68	0,85	0,67	0,79	0,69	0,88	0,70	0,82	0,68	0,67	0,61	0,73	0,66	0,66	0,84	0,64	0,66	0,81	0,62
NC_005226.1	0,70	0,78	0,82	0,64	0,68	0,66	0,68	0,68	0,82	0,70	0,80	0,69	0,81	0,69	0,88	0,71	0,69	0,64	0,79	0,69	0,66	0,80	0,66	0,64	0,89	0,62
NC_006435.1	0,82	0,66	0,69	0,55	0,81	0,69	0,77	0,70	0,71	0,72	0,73	0,81	0,66	0,84	0,70	0,82	0,80	0,65	0,68	0,77	0,77	0,66	0,75	0,68	0,71	0,64

NC_055170.1	0,64	0,63	0,64	0,53	0,63	0,63	0,68	0,69	0,66	0,67	0,66	0,66	0,63	0,66	0,66	0,66	0,70	0,65	0,65	0,69	0,69	0,66	0,65	0,60	0,67	0,60
AF291704.5	0,67	0,93	0,87	0,72	0,67	0,66	0,69	0,69	0,85	0,68	0,81	0,70	0,87	0,69	0,83	0,69	0,71	0,66	0,77	0,68	0,66	0,87	0,66	0,66	0,84	0,61
AY526217.1	0,66	0,77	0,78	0,61	0,66	0,66	0,68	0,66	0,82	0,72	0,81	0,68	0,79	0,67	0,91	0,66	0,69	0,64	0,78	0,71	0,66	0,75	0,67	0,63	0,86	0,62
DQ371906.1	0,83	0,64	0,67	0,54	0,81	0,68	0,77	0,70	0,74	0,72	0,72	0,80	0,66	0,82	0,70	0,81	0,79	0,65	0,67	0,77	0,77	0,67	0,76	0,67	0,71	0,64
DQ825770.1	0,69	0,65	0,66	0,56	0,66	0,75	0,68	0,70	0,65	0,72	0,66	0,65	0,65	0,71	0,65	0,70	0,68	0,86	0,63	0,66	0,66	0,67	0,66	0,69	0,66	0,69
DQ989237.1	0,81	0,63	0,67	0,53	0,81	0,65	0,73	0,71	0,71	0,71	0,71	0,79	0,66	0,81	0,67	0,79	0,77	0,62	0,65	0,76	0,72	0,65	0,72	0,66	0,67	0,62
EF397003.1	0,71	0,86	0,85	0,81	0,68	0,66	0,66	0,71	0,82	0,69	0,81	0,69	0,86	0,71	0,82	0,71	0,68	0,68	0,75	0,68	0,67	0,86	0,67	0,66	0,83	0,62
EF646763.1	0,71	0,77	0,78	0,65	0,70	0,68	0,69	0,67	0,81	0,71	0,84	0,71	0,81	0,71	0,91	0,71	0,72	0,65	0,84	0,71	0,69	0,75	0,69	0,66	0,91	0,66
EU788002.1	0,66	0,87	0,86	0,70	0,65	0,61	0,65	0,66	0,83	0,68	0,77	0,66	0,88	0,69	0,80	0,66	0,67	0,63	0,76	0,66	0,66	0,86	0,62	0,63	0,81	0,61
GU140096.1	0,82	0,65	0,70	0,54	0,82	0,69	0,78	0,70	0,71	0,73	0,73	0,81	0,66	0,83	0,69	0,82	0,79	0,66	0,68	0,77	0,77	0,66	0,75	0,68	0,71	0,64
FJ858378.1	0,67	0,83	0,84	0,71	0,69	0,68	0,68	0,66	0,84	0,72	0,82	0,69	0,88	0,71	0,84	0,68	0,71	0,66	0,78	0,72	0,70	0,80	0,66	0,68	0,87	0,65
GQ244526.1	0,66	0,84	0,85	0,71	0,67	0,67	0,68	0,67	0,82	0,72	0,82	0,69	0,87	0,70	0,82	0,68	0,70	0,66	0,77	0,71	0,69	0,80	0,66	0,69	0,84	0,65
HM015220.1	0,69	0,71	0,71	0,58	0,70	0,67	0,64	0,65	0,73	0,66	0,76	0,63	0,73	0,69	0,79	0,68	0,66	0,66	0,72	0,66	0,63	0,70	0,67	0,68	0,79	0,62
JQ026206.1	0,87	0,65	0,69	0,55	0,98	0,68	0,74	0,71	0,71	0,72	0,71	0,79	0,68	0,86	0,71	0,89	0,79	0,68	0,67	0,80	0,75	0,68	0,80	0,68	0,70	0,62
JQ083393.1	0,82	0,66	0,69	0,55	0,81	0,69	0,77	0,70	0,71	0,72	0,73	0,81	0,66	0,84	0,70	0,82	0,80	0,65	0,68	0,77	0,77	0,66	0,75	0,68	0,71	0,64
JN831945.1	0,66	0,79	0,80	0,62	0,67	0,68	0,70	0,66	0,83	0,72	0,80	0,69	0,80	0,68	0,92	0,66	0,71	0,65	0,81	0,72	0,66	0,76	0,70	0,65	0,87	0,62
JX853574.1	0,98	0,67	0,68	0,58	0,87	0,68	0,75	0,74	0,71	0,69	0,72	0,79	0,68	0,87	0,71	0,85	0,77	0,67	0,68	0,75	0,72	0,70	0,75	0,67	0,71	0,62
JX879770.1	0,98	0,67	0,68	0,58	0,87	0,68	0,75	0,74	0,71	0,69	0,72	0,79	0,68	0,87	0,71	0,85	0,77	0,67	0,68	0,75	0,72	0,70	0,75	0,67	0,71	0,62
JX028271.1	0,66	0,77	0,83	0,63	0,69	0,69	0,68	0,66	0,81	0,72	0,83	0,69	0,82	0,69	0,93	0,66	0,69	0,65	0,81	0,71	0,65	0,76	0,68	0,65	0,89	0,66
KF705679.1	0,72	0,65	0,69	0,51	0,75	0,62	0,77	0,71	0,71	0,73	0,71	0,88	0,66	0,77	0,68	0,77	0,85	0,60	0,65	0,83	0,77	0,67	0,77	0,67	0,68	0,62
KF537001.1	0,71	0,86	0,89	0,70	0,68	0,64	0,66	0,68	0,85	0,67	0,79	0,69	0,88	0,70	0,82	0,68	0,67	0,61	0,73	0,66	0,66	0,84	0,64	0,66	0,81	0,62
KM102249.1	0,71	0,64	0,66	0,55	0,70	0,78	0,67	0,67	0,66	0,71	0,66	0,66	0,66	0,69	0,68	0,72	0,71	0,74	0,66	0,72	0,70	0,64	0,69	0,76	0,71	0,67
KR072623.1	0,68	0,61	0,65	0,51	0,69	0,73	0,65	0,66	0,64	0,63	0,66	0,67	0,65	0,70	0,66	0,69	0,68	0,75	0,61	0,69	0,65	0,66	0,66	0,70	0,66	0,70
KM361055.1	0,63	0,60	0,63	0,50	0,62	0,72	0,62	0,62	0,62	0,69	0,66	0,66	0,63	0,64	0,66	0,65	0,67	0,68	0,62	0,66	0,66	0,62	0,64	0,70	0,65	1,00
KM361056.1	0,63	0,60	0,63	0,50	0,62	0,72	0,62	0,62	0,62	0,69	0,66	0,66	0,63	0,64	0,66	0,65	0,67	0,68	0,62	0,66	0,66	0,62	0,64	0,70	0,65	1,00
KM361061.1	0,64	0,61	0,62	0,49	0,61	0,70	0,63	0,63	0,62	0,68	0,66	0,65	0,63	0,63	0,65	0,64	0,66	0,68	0,63	0,65	0,66	0,62	0,63	0,69	0,64	0,98
KT885041.1	0,86	0,66	0,71	0,56	0,98	0,69	0,73	0,71	0,72	0,71	0,73	0,80	0,68	0,84	0,69	0,87	0,78	0,66	0,67	0,79	0,74	0,69	0,79	0,68	0,69	0,62
KT885044.1	0,71	0,86	0,89	0,70	0,68	0,64	0,66	0,68	0,85	0,67	0,79	0,69	0,88	0,70	0,82	0,68	0,67	0,61	0,73	0,66	0,66	0,84	0,64	0,66	0,81	0,62
KT885047.1	0,83	0,66	0,68	0,55	0,80	0,68	0,77	0,70	0,71	0,72	0,72	0,81	0,66	0,84	0,70	0,82	0,80	0,66	0,67	0,77	0,77	0,66	0,75	0,68	0,71	0,64
KT885050.1	0,66	0,78	0,81	0,63	0,67	0,69	0,70	0,66	0,82	0,72	0,81	0,69	0,81	0,68	0,92	0,66	0,71	0,64	0,80	0,72	0,66	0,75	0,70	0,66	0,87	0,63
KT934965.1	0,83	0,66	0,68	0,55	0,80	0,68	0,77	0,70	0,71	0,72	0,72	0,81	0,66	0,84	0,70	0,82	0,80	0,66	0,67	0,77	0,77	0,66	0,75	0,68	0,71	0,64
KU215675.1	0,82	0,65	0,70	0,54	0,82	0,69	0,78	0,70	0,71	0,73	0,73	0,81	0,66	0,83	0,69	0,82	0,79	0,66	0,68	0,77	0,77	0,66	0,75	0,68	0,71	0,64
KX845680.1	0,68	0,65	0,69	0,56	0,68	0,77	0,68	0,66	0,66	0,71	0,70	0,67	0,70	0,71	0,69	0,71	0,71	0,78	0,64	0,68	0,67	0,70	0,69	0,74	0,69	0,74
KY594712.1	0,82	0,66	0,69	0,55	0,81	0,69	0,77	0,70	0,71	0,72	0,73	0,81	0,66	0,84	0,70	0,82	0,80	0,65	0,68	0,77	0,77	0,66	0,75	0,68	0,71	0,64
KY662269.1	0,69	0,66	0,69	0,54	0,67	0,75	0,66	0,64	0,69	0,70	0,67	0,67	0,68	0,68	0,67	0,67	0,71	0,68	0,67	0,72	0,69	0,69	0,65	0,73	0,67	0,65
KY662275.1	0,70	0,66	0,67	0,53	0,67	0,77	0,68	0,65	0,69	0,69	0,69	0,66	0,67	0,70	0,68	0,66	0,72	0,69	0,69	0,72	0,71	0,67	0,66	0,75	0,68	0,67
MG717393.1	0,71	0,85	0,87	0,71	0,71	0,65	0,66	0,69	0,87	0,68	0,82	0,70	0,87	0,73	0,83	0,69	0,70	0,63	0,74	0,67	0,70	0,83	0,67	0,67	0,83	0,63
KY978757.1	0,81	0,66	0,69	0,55	0,82	0,68	0,76	0,70	0,71	0,71	0,73	0,81	0,65	0,82	0,68	0,82	0,78	0,63	0,66	0,75	0,75	0,66	0,73	0,67	0,69	0,63
MN183135.1	0,69	0,80	0,82	0,64	0,69	0,68	0,69	0,67	0,82	0,71	0,82	0,69	0,82	0,69	0,89	0,69	0,70	0,64	0,81	0,69	0,66	0,79	0,68	0,65	0,89	0,64
MN850093.1	0,67	0,93	0,87	0,71	0,67	0,66	0,69	0,69	0,86	0,68	0,81	0,70	0,87	0,69	0,83	0,69	0,71	0,66	0,77	0,68	0,67	0,87	0,66	0,66	0,85	0,61
MN850096.1	0,67	0,93	0,86	0,71	0,67	0,66	0,69	0,69	0,86	0,68	0,81	0,70	0,87	0,69	0,84	0,69	0,71	0,66	0,77	0,68	0,67	0,86	0,66	0,66	0,85	0,61
MN639737.1	0,65	0,78	0,81	0,61	0,66	0,67	0,70	0,66	0,82	0,72	0,79	0,68	0,80	0,68	0,90	0,66	0,71	0,64	0,80	0,71	0,66	0,77	0,69	0,65	0,85	0,62
MN639740.1	0,65	0,78	0,81	0,61	0,66	0,67	0,70	0,66	0,82	0,72	0,79	0,68	0,80	0,68	0,90	0,66	0,71	0,64	0,80	0,71	0,66	0,77	0,69	0,65	0,85	0,62
MT024592.1	0,66	0,79	0,80	0,62	0,67	0,68	0,70	0,66	0,83	0,72	0,80	0,69	0,80	0,68	0,92	0,66	0,71	0,65	0,81	0,72	0,66	0,76	0,70	0,65	0,87	0,62
MT514291.1	0,65	0,77	0,82	0,62	0,66	0,68	0,70	0,65	0,81	0,72	0,80	0,68	0,81	0,68	0,91	0,66	0,71	0,63	0,79	0,71	0,66	0,76	0,69	0,66	0,86	0,63
MT514292.1	0,66	0,77	0,81	0,61	0,66	0,66	0,69	0,66	0,81	0,72	0,79	0,68	0,80	0,68	0,90	0,66	0,70	0,64	0,79	0,70	0,66	0,78	0,67	0,64	0,85	0,62
MT514294.1	0,66	0,79	0,80	0,62	0,67	0,68	0,70	0,66	0,83	0,72	0,80	0,69	0,80	0,68	0,92	0,66	0,71	0,65	0,81	0,72	0,66	0,76	0,70	0,65	0,87	0,62

MT514295.1	0,70	0,78	0,82	0,64	0,68	0,66	0,68	0,68	0,82	0,70	0,80	0,69	0,81	0,69	0,88	0,71	0,69	0,64	0,79	0,69	0,66	0,80	0,66	0,64	0,89	0,62
MT514296.1	0,69	0,80	0,82	0,64	0,69	0,68	0,69	0,67	0,82	0,71	0,82	0,69	0,82	0,69	0,89	0,69	0,70	0,65	0,81	0,69	0,66	0,80	0,68	0,65	0,89	0,64
MT514297.1	0,69	0,81	0,82	0,65	0,68	0,69	0,68	0,67	0,82	0,70	0,82	0,67	0,82	0,69	0,90	0,69	0,70	0,66	0,81	0,68	0,66	0,79	0,66	0,66	0,89	0,64
MT514298.1	0,70	0,78	0,82	0,64	0,68	0,66	0,68	0,68	0,82	0,70	0,80	0,69	0,81	0,69	0,88	0,71	0,69	0,64	0,79	0,69	0,66	0,80	0,66	0,64	0,89	0,62
MN258157.1	0,67	0,93	0,87	0,71	0,67	0,66	0,69	0,69	0,86	0,68	0,81	0,70	0,87	0,69	0,83	0,69	0,71	0,66	0,77	0,68	0,67	0,87	0,66	0,66	0,85	0,61
MN832779.1	0,66	0,77	0,79	0,62	0,67	0,67	0,69	0,66	0,82	0,73	0,82	0,69	0,80	0,68	0,92	0,66	0,70	0,65	0,79	0,71	0,67	0,76	0,68	0,64	0,87	0,63
MN832782.1	0,66	0,79	0,80	0,62	0,67	0,68	0,70	0,66	0,83	0,72	0,80	0,69	0,80	0,68	0,92	0,66	0,71	0,65	0,81	0,72	0,66	0,76	0,70	0,65	0,87	0,62
MT648514.1	0,61	0,59	0,62	0,48	0,61	0,67	0,61	0,61	0,61	0,62	0,62	0,68	0,60	0,62	0,61	0,64	0,66	0,65	0,59	0,65	0,61	0,61	0,61	0,69	0,63	0,87
OM030302.1	0,70	0,66	0,71	0,57	0,71	0,75	0,66	0,67	0,69	0,69	0,71	0,70	0,69	0,71	0,71	0,71	0,70	0,74	0,65	0,69	0,69	0,68	0,68	0,72	0,71	0,67
OM030305.1	0,82	0,64	0,68	0,54	0,82	0,66	0,75	0,71	0,72	0,71	0,72	0,81	0,66	0,82	0,68	0,80	0,78	0,63	0,66	0,77	0,74	0,66	0,74	0,67	0,69	0,64
OM030311.1	0,75	0,66	0,67	0,54	0,77	0,66	0,79	0,69	0,70	0,78	0,71	0,87	0,66	0,79	0,70	0,80	0,83	0,62	0,66	0,84	0,79	0,71	0,78	0,71	0,68	0,66
OK422869.1	0,86	0,65	0,70	0,55	0,99	0,68	0,74	0,71	0,71	0,71	0,72	0,81	0,67	0,84	0,68	0,88	0,77	0,66	0,66	0,80	0,73	0,68	0,79	0,69	0,68	0,61
MZ504241.1	1,00	0,66	0,67	0,57	0,87	0,69	0,75	0,75	0,71	0,69	0,71	0,78	0,67	0,88	0,70	0,86	0,77	0,68	0,67	0,76	0,74	0,69	0,75	0,67	0,70	0,63
OQ092243.1	0,66	1,00	0,84	0,70	0,66	0,63	0,66	0,67	0,83	0,66	0,77	0,68	0,85	0,66	0,81	0,68	0,68	0,65	0,75	0,66	0,65	0,86	0,64	0,65	0,79	0,60
OR148904.1	0,67	0,84	1,00	0,67	0,71	0,66	0,70	0,70	0,87	0,71	0,82	0,73	0,90	0,70	0,82	0,69	0,70	0,65	0,76	0,70	0,66	0,87	0,67	0,69	0,82	0,63
OR365538.1	0,57	0,70	0,67	1,00	0,56	0,54	0,53	0,57	0,66	0,58	0,65	0,55	0,69	0,57	0,65	0,57	0,55	0,55	0,61	0,55	0,54	0,68	0,55	0,52	0,66	0,50
NC_005235.1	0,87	0,66	0,71	0,56	1,00	0,69	0,73	0,71	0,72	0,71	0,73	0,80	0,68	0,85	0,69	0,87	0,78	0,67	0,67	0,79	0,74	0,69	0,78	0,68	0,69	0,62
NC_034401.1	0,69	0,63	0,66	0,54	0,69	1,00	0,70	0,66	0,66	0,71	0,68	0,65	0,65	0,67	0,69	0,71	0,69	0,73	0,65	0,70	0,67	0,64	0,70	0,80	0,71	0,72
NC_034399.1	0,75	0,66	0,70	0,53	0,73	0,70	1,00	0,75	0,71	0,77	0,71	0,80	0,68	0,76	0,69	0,77	0,81	0,66	0,69	0,82	0,78	0,65	0,78	0,69	0,71	0,62
NC_034402.1	0,75	0,67	0,70	0,57	0,71	0,66	0,75	1,00	0,71	0,76	0,71	0,75	0,65	0,71	0,67	0,76	0,77	0,69	0,70	0,75	0,73	0,70	0,74	0,64	0,68	0,62
NC_034403.1	0,71	0,83	0,87	0,66	0,72	0,66	0,71	0,71	1,00	0,71	0,81	0,74	0,86	0,73	0,82	0,69	0,74	0,66	0,79	0,73	0,71	0,83	0,70	0,66	0,85	0,62
NC_034407.1	0,69	0,66	0,71	0,58	0,71	0,71	0,77	0,76	0,71	1,00	0,74	0,74	0,71	0,73	0,71	0,71	0,77	0,69	0,71	0,76	0,74	0,70	0,77	0,68	0,71	0,69
NC_034467.1	0,71	0,77	0,82	0,65	0,73	0,68	0,71	0,71	0,81	0,74	1,00	0,74	0,82	0,74	0,86	0,71	0,73	0,66	0,87	0,71	0,70	0,76	0,71	0,66	0,85	0,66
NC_034485.1	0,78	0,68	0,73	0,55	0,80	0,65	0,80	0,75	0,74	0,74	0,74	1,00	0,68	0,82	0,71	0,81	0,87	0,63	0,66	0,87	0,77	0,72	0,77	0,72	0,71	0,66
NC_034515.1	0,67	0,85	0,90	0,69	0,68	0,65	0,68	0,65	0,86	0,71	0,82	0,68	1,00	0,71	0,85	0,65	0,68	0,65	0,77	0,68	0,66	0,84	0,66	0,67	0,85	0,63
NC_034517.1	0,88	0,66	0,70	0,57	0,85	0,67	0,76	0,71	0,73	0,73	0,74	0,82	0,71	1,00	0,71	0,83	0,82	0,69	0,70	0,80	0,77	0,70	0,76	0,69	0,71	0,64
NC_034519.1	0,70	0,81	0,82	0,65	0,69	0,69	0,69	0,67	0,82	0,71	0,86	0,71	0,85	0,71	1,00	0,71	0,72	0,66	0,82	0,71	0,67	0,78	0,69	0,67	0,92	0,66
NC_034556.1	0,86	0,68	0,69	0,57	0,87	0,71	0,77	0,76	0,69	0,71	0,71	0,81	0,65	0,83	0,71	1,00	0,81	0,68	0,67	0,80	0,77	0,69	0,78	0,70	0,70	0,65
NC_034560.1	0,77	0,68	0,70	0,55	0,78	0,69	0,81	0,77	0,74	0,77	0,73	0,87	0,68	0,82	0,72	0,81	1,00	0,66	0,71	0,92	0,82	0,70	0,81	0,70	0,72	0,67
NC_034564.1	0,68	0,65	0,65	0,55	0,67	0,73	0,66	0,69	0,66	0,69	0,66	0,63	0,65	0,69	0,66	0,68	0,66	1,00	0,64	0,65	0,64	0,67	0,66	0,69	0,67	0,68
NC_038529.1	0,67	0,75	0,76	0,61	0,67	0,65	0,69	0,70	0,79	0,71	0,87	0,66	0,77	0,70	0,82	0,67	0,71	0,64	1,00	0,69	0,66	0,71	0,68	0,63	0,82	0,62
NC_043068.1	0,76	0,66	0,70	0,55	0,79	0,70	0,82	0,75	0,73	0,76	0,71	0,87	0,68	0,80	0,71	0,80	0,92	0,65	0,69	1,00	0,82	0,70	0,81	0,71	0,71	0,66
NC_043175.1	0,74	0,65	0,66	0,54	0,74	0,67	0,78	0,73	0,71	0,74	0,70	0,77	0,66	0,77	0,67	0,77	0,82	0,64	0,66	0,82	1,00	0,69	0,78	0,66	0,70	0,66
NC_043407.1	0,69	0,86	0,87	0,68	0,69	0,64	0,65	0,70	0,83	0,70	0,76	0,72	0,84	0,70	0,78	0,69	0,70	0,67	0,71	0,70	0,69	1,00	0,66	0,64	0,79	0,62
NC_055147.1	0,75	0,64	0,67	0,55	0,78	0,70	0,78	0,74	0,70	0,77	0,71	0,77	0,66	0,76	0,69	0,78	0,81	0,66	0,68	0,81	0,78	0,66	1,00	0,70	0,70	0,64
NC_055632.1	0,67	0,65	0,69	0,52	0,68	0,80	0,69	0,64	0,66	0,68	0,66	0,72	0,67	0,69	0,67	0,70	0,70	0,69	0,63	0,71	0,66	0,64	0,70	1,00	0,67	0,70
NC_055636.1	0,70	0,79	0,82	0,66	0,69	0,71	0,71	0,68	0,85	0,71	0,85	0,71	0,85	0,71	0,92	0,70	0,72	0,67	0,82	0,71	0,70	0,79	0,70	0,67	1,00	0,65
NC_078485.1	0,63	0,60	0,63	0,50	0,62	0,72	0,62	0,62	0,62	0,69	0,66	0,66	0,63	0,64	0,66	0,65	0,67	0,68	0,62	0,66	0,66	0,62	0,64	0,70	0,65	1,00
